# Antioxidant Therapy in Inflammatory Bowel Diseases: How Far Have We Come and How Close Are We?

**DOI:** 10.3390/antiox13111369

**Published:** 2024-11-08

**Authors:** Lylian Ellen Militão dos Santos Xavier, Thays Cristhyna Guimaraes Reis, Amylly Sanuelly da Paz Martins, Juliana Célia de Farias Santos, Nassib Bezerra Bueno, Marília Oliveira Fonseca Goulart, Fabiana Andréa Moura

**Affiliations:** 1 Postgraduate Degree in Nutrition (PPGNUT), Federal University of Alagoas (UFAL), Maceió 57072-970, AL, Brazil; lylian.santos@fanut.ufal.br (L.E.M.d.S.X.); nassib.bueno@fanut.ufal.br (N.B.B.); 2 Faculty of Nutrition (FANUT), Federal University of Alagoas (UFAL), Maceió 57072-970, AL, Brazil; thays.reis@fanut.ufal.br; 3 Postgraduate Studies at the Northeast Biotechnology Network (RENORBIO), Federal University of Alagoas (UFAL), Maceió 57072-970, AL, Brazil; amylly.martins@iqb.ufal.br; 4 Postgraduate Degree in Medical Sciences (PPGCM/UFAL), Federal University of Alagoas (UFAL), Maceió 57072-970, AL, Brazil; juliana.santos@fanut.ufal.br; 5 Institute of Chemistry and Biotechnology (IQB/UFAL), Federal University of Alagoas (UFAL), Maceió 57072-970, AL, Brazil

**Keywords:** oxidative stress, biomarkers, complementary and alternative medicine, ulcerative colitis, Crohn’s disease

## Abstract

Inflammatory bowel diseases (IBD) pose a growing public health challenge with unclear etiology and limited efficacy of traditional pharmacological treatments. Alternative therapies, particularly antioxidants, have gained scientific interest. This systematic review analyzed studies from MEDLINE, Cochrane, Web of Science, EMBASE, and Scopus using keywords like “Inflammatory Bowel Diseases” and “Antioxidants.” Initially, 925 publications were identified, and after applying inclusion/exclusion criteria—covering studies from July 2015 to June 2024 using murine models or clinical trials in humans and evaluating natural or synthetic substances affecting oxidative stress markers—368 articles were included. This comprised 344 animal studies and 24 human studies. The most investigated antioxidants were polyphenols and active compounds from medicinal plants (n = 242; 70.3%). The review found a strong link between oxidative stress and inflammation in IBD, especially in studies on nuclear factor kappa B and nuclear factor erythroid 2-related factor 2 pathways. However, it remains unclear whether inflammation or oxidative stress occurs first in IBD. Lipid peroxidation was the most studied oxidative damage, followed by DNA damage. Protein damage was rarely investigated. The relationship between antioxidants and the gut microbiota was examined in 103 animal studies. Human studies evaluating oxidative stress markers were scarce, reflecting a major research gap in IBD treatment. PROSPERO registration: CDR42022335357 and CRD42022304540.

## 1. Introduction

The etiology of inflammatory bowel disease (IBD), which is mostly represented by Crohn’s disease (CD) and ulcerative colitis (UC), is largely unknown. IBD is regarded as a global public health concern. It is believed to be caused by changes in the immune system, gut microbiota, and external environment in addition to an individual’s genetic vulnerability [1]. Its incidence stabilized in the 21st century, although it is still a major epidemiological concern in economically developed nations like those in North America and Europe. Conversely, the incidence and prevalence of UC and CD have been rising gradually in developing countries, approaching Western nation rates with each passing decade [2]. 

Treatment is directly impacted, as previously said, by the absence of specific pharmacological targets in the etiology of IBD. Among the drugs used today are the following: (i) Aminosalicylates, of which sulfasalazine is the most well-known, which has been used for treating IBD for 80 years. Its mechanisms of action include changes in the metabolism of arachidonic acid, the removal of reactive oxygen species (ROS), effects on white blood cells, and a decrease in the production of cytokines [3]. (ii) Corticosteroids, which are used to induce remission in patients with moderate to severe inflammation or who are not responsive to mesalazine. They work by interacting with pro-inflammatory transcription factors such as activator protein 1 (AP-1) and nuclear factor kappa B (NF-κB), which inhibits the transcription of some inflammatory genes and directly reduces cytokines like interleukin—IL-1α, IL-1ß, and IL-8 [4]; (iii) immunosuppressive therapy, which inhibits antigen-induced lymphokine secretion by binding to Ca^2+^ calmodulin-dependent protein phosphatase calcineurin [5]; and (iv) biological treatments, such as integrin antagonists and tumor necrosis factor-alpha (TNF-α) inhibitors, which were introduced in the late 1990s and are directly linked to improved quality of life for individuals with inflammatory bowel disease [6].

Many side effects continue despite the different pharmacological alternatives that make up the therapeutic pyramid for individuals with IBD. These include abdominal pain, nausea, vomiting, anorexia, headache, hemolysis, pancreatitis, and worsening diarrhea (aminosalicylates) [7]; hyperglycemia, hyperlipidemia, Cushingoid appearance, and metabolic fatty liver disease (corticosteroids) [8]; myelotoxicity, gastrointestinal intolerance, hepatotoxicity, malignancy, pancreatitis, fever, and skin rash (immunomodulators) [9]; uveitis, vasculitis, psoriasis, sarcoidosis, and even the paradoxical effects of inducing or exacerbating conditions intended to be treated (biological therapy) [10].

Due to the numerous side effects and the fact that many patients do not respond to traditional treatment, often requiring surgical procedures, several alternative therapies are being researched as adjuncts to standard IBD medication. Among these, the use of substances with antioxidant properties stands out. Reactive oxygen and nitrogen species (RONS) and the inflammatory process are directly linked to oxidative imbalance, which is closely linked to the onset and progression of inflammatory bowel disease (IBD). This is because oxidative and nitrosative stress not only affects the inflammatory intestinal mucosa but also penetrates deeper intestinal wall layers and is reflected in the body’s circulation [11].

In 2015, our research group published a systematic review of 134 articles evaluating the action of oral antioxidant therapy on oxidative stress markers in IBD, with 130 studies on murine models and only 4 on humans [12]. 

Despite these findings, several issues remain unresolved, the most important of which is: Should patients with IBD receive antioxidant therapy? Consequently, this update’s goal is to analyze the antioxidant and anti-inflammatory properties of antioxidant therapies that have been studied for IBD in both human and murine models.

## 2. Materials and Methods

### 2.1. Search Strategy and Selection of Studies

The search was conducted from July 2015 to June 2024 across the following databases: MEDLINE (via PubMed), Cochrane Controlled Register of Trials (CENTRAL), Web of Science, EMBASE, and Scopus. The following keywords were used: Inflammatory Bowel Diseases; Antioxidants; Antioxidant Therapy; Crohn’s Disease; Ulcerative Colitis; Biomarkers; Oxidative Stress. Boolean operators “OR” and “AND” were applied, with the complete search strategy, including the following terms: “inflammatory bowel disease” AND “antioxidant” AND (“therapy” OR “treatment”). All retrieved records had their titles and abstracts screened. Subsequently, two independent researchers reviewed the titles to remove duplicate records. Articles cited by the authors of the found studies were also included.

### 2.2. Eligibility of Clinical Research

Inclusion Criteria:

(i) Studies with rodents (rats or mice, due to biological similarity to humans); experimental IBD induced by drugs or genetic modification (knockout animals); acute or chronic IBD models, published from July 2015, in English, Spanish, or Portuguese.
(ii) Studies with patients diagnosed with CD or UC.
(iii) Use of natural or synthetic substances/compounds, or food/plants with oral/gavage antioxidant action isolated or combined with traditional therapies.
(iv) The antioxidant action was determined when the studied compounds exhibited one of the following actions:
  - Action on ROS/RNS: such as nitrite, nitric oxide, and others.
  - Effect on RONS synthesis: influence on activity, protein expression, or gene expression of inducible nitric oxide synthase (iNOS), cyclooxygenase 2 (COX2), nicotinamide adenine dinucleotide phosphate (NADPH)-oxidase (NOX), lipoxygenase (LOX), myeloperoxidase (MPO), NF-κB or IκBα (nuclear factor of kappa light polypeptide gene enhancer in B-cells inhibitor, alpha), and nuclear factor erythroid 2-related factor 2 (Nrf2).
  - Effect on oxidative damage: damage to cell membranes (thiobarbituric acid reactive substances—TBARS, malondialdehyde—MDA, 4-hydroxynonenal—HNE, isoprostanes, and others), protein damage (protein carbonylation, advanced glycation end-products—AGE, and others), and DNA damage (8-oxo-2′-deoxyguanosine, 8-oxoguanine, and others).
  - Action on antioxidant defense: enzymatic (superoxide dismutase—SOD, catalase—CAT, glutathione peroxidase—GPx, glutathione reductase—GR) and non-enzymatic (reduced glutathione—GSH and GSH/GSSG ratio) endogenous antioxidant defense and total antioxidant status (TAS) or total antioxidant capacity (TAC).
  - Effect on intestinal microbiota modulation.

Exclusion Criteria:

(i) Studies utilizing a combination of antioxidant substances, except plants or foods, or medicaments + antioxidant.

Classification of Antioxidant Substances:

The studies were categorized into the following therapeutic topics:

(i) Hormones
(ii) Synthetic or hemisynthetic compounds.
(iii) Chemical products derived from non-plant sources.
(iv) Polyphenols and other active compounds from medicinal plants.
(v) Functional foods and antioxidant nutrients.
(vi) Probiotics.
(vii) Others.

Record: 

This systematic review was registered in the International Prospective Register of Systematic Reviews (PROSPERO) nº CDR42022335357 and CRD42022304540.

### 2.3. Data Extraction

The following data were extracted from the studies: supplement presentation; doses; duration of supplementation; route of administration; treatment groups; effects on oxidative stress biomarkers; and action on the intestinal microbiota. Studies with multiple supplementation doses were allocated according to the highest dose.

### 2.4. Risk of Bias and Quality of Evidence Assessment

#### 2.4.1. Animal Studies

The Systematic Review Centre for Laboratory Animal Experimentation (SYRCLE) tool was used to assess the risk of bias. This tool evaluates ten domains: random sequence generation, baseline characteristics, allocation concealment, random housing, blinding of caregivers and researchers, random outcome assessment, blinding of outcome assessors, incomplete outcome data, selective outcome reporting, and other sources of bias. Each category was rated as having low risk, high risk, or unclear risk of bias using the software RevMan 5.4 (The Nordic Cochrane Centre, The Cochrane Collaboration, Denmark).

#### 2.4.2. Randomized Clinical Trials (RCTs)

The risk of bias in randomized clinical trials (RCTs) was evaluated using the Cochrane risk of bias tool, which assesses six domains: random sequence generation, allocation concealment, blinding of participants and personnel, blinding of outcome assessment, incomplete outcomes data (intention-to-treat or per-protocol analysis), and selective reporting. Studies that did not present a registered clinical protocol were classified as high-risk in the “selective outcome report” domain.

#### 2.4.3. Non-Randomized Controlled Studies

For non-randomized controlled studies, the ROBINS-I (Reduction of Bias In Non-randomized Studies of Interventions) tool was used (available at https://www.riskofbias.info/, accessed on 20 July 2024), evaluating seven domains: confounding, participant selection, classification of interventions, deviations from intended interventions, missing data, outcome measurement, and selection of the reported result.

## 3. Results and Discussion

**Figure 1:**
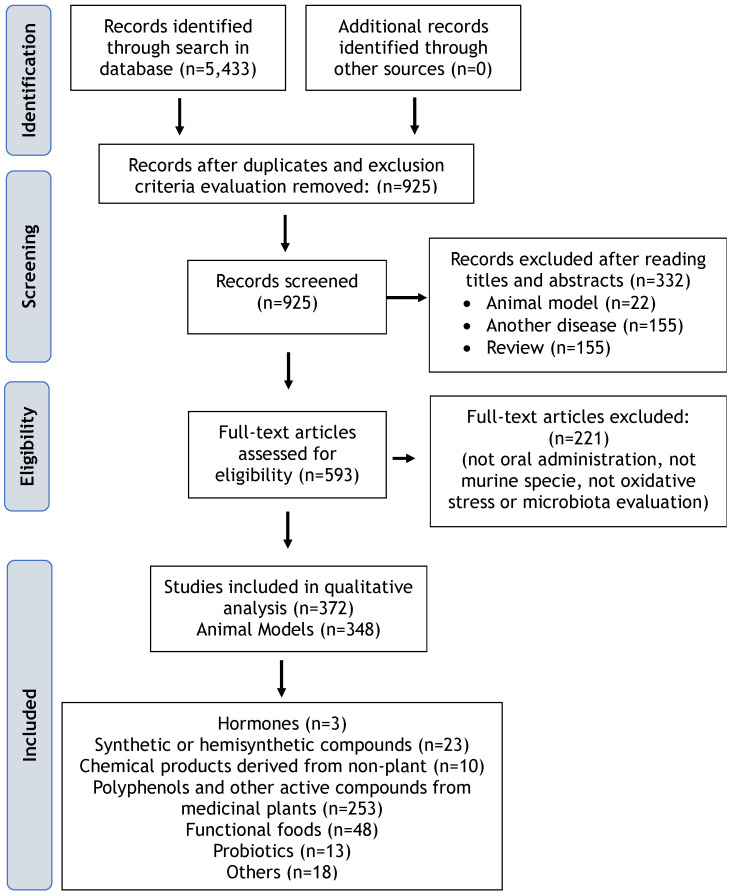
Flowchart with the main results of the database search.

Initially, 925 studies were identified after duplicate removal and exclusion criteria evaluation. Following title and abstract screening and full-text reading, 369 studies were included in this review, comprising 344 (93.4%) animal model studies and 24 (6.5%) clinical studies involving humans (Figure 1). The most investigated category of antioxidants in animals was “polyphenols and other active compounds from medicinal plants” (n = 242; 70.3%), followed by functional foods, antioxidant nutrients, probiotics, among others (n = 67; 19.4%), and hormones, synthetic or hemisynthetic compounds, and chemical products derived from non-plant sources (n = 36; 10.4%).

In human studies, the most investigated category of antioxidants was “functional foods, antioxidant nutrients” (n = 12; 50%), followed by “polyphenols and other active compounds from medicinal plants” (n = 11; 45.8%), and “others” (n = 1; 4.1%).

### 3.1. Risk of Bias

The elements of allocation concealment, randomization of treatment allocation, blinding of caregivers and researchers, randomized outcome assessment, and blinding of outcome assessors are fundamental in scientific research. They help ensure the comparability of experimental groups and prevent bias from influencing results. In our systematic review of animal studies, as shown in Supplementary Figure S1A, these elements were identified as having a high risk of bias due to a lack of information on whether and how these procedures were implemented, which compromises the validity and reliability of the study results.

The analysis of bias risk in randomized clinical trials is provided in Supplementary Figure S1B. Most categories exhibit a low risk of bias, with only a small proportion classified as uncertain risk. In most cases, the necessary information to assess risk was available in the analyzed categories. However, studies that lacked a registered clinical protocol scored high in the “selective outcome reporting” category.

For non-randomized clinical trials, while some categories of bias—such as outcome blinding—are better controlled, others, such as random sequence generation and selective reporting, present concerning levels of risk. These issues could undermine the integrity of the trial results (see Supplementary Figure S1B).

### 3.2. Oxidative Stress and Inflammation in IBD: Which Comes First?

Given that oxidative stress plays a crucial role in the pathogenesis of IBD, various antioxidant therapeutic strategies are being explored, including reactive species removal, increased synthesis of antioxidant enzymes, and inhibition of abnormal redox signaling for reactions. Even though oxidative stress is considered a potential pathogenic and critical factor in the initiation, progression, and severity of IBD by actively participating in chronic mucosal inflammation, the underlying mechanisms remain far from being fully elucidated. Based on laboratory reports and clinical trials, a growing number of antioxidants, such as RONS generation inhibitors, hormones, synthetic substances, polyphenols, plant extracts, functional foods, micronutrients, and probiotics, are being investigated as potential therapeutic strategies aimed at minimizing oxidative stress in IBD (Table 1 and Table S1) [13,14,15]. 

However, understanding the sequence of events—whether oxidative stress or inflammation occurs first—is crucial for the development of effective treatments. In IBD, as in other diseases, redox imbalance is a mechanism intrinsically related to its etiopathogenesis. At the intestinal level, oxidative stress regulates gene expression directly involved with both innate and adaptive immune responses. One consequence of the interaction between RONS and endobiotics is molecular and tissue damage, impairing molecule and tissue functionality, increasing permeability, and fostering chronic inflammation in the intestinal mucosa [16]. This occurs through the activation of pro-inflammatory pathways such as NF-κB, which acts on pro-inflammatory genes and cytokine and chemokine encoding, and the inactivation of anti-inflammatory mediators like Nrf2, nuclear factor, which induces the biosynthesis of a set of antioxidants and detoxifying enzymes, such as GPx, SOD, and CAT [17].

At the same time, NF-κB and the mitogen-activated protein kinase (MAPK) pathways are activated in response to inflammatory activity, such as the binding of TNF-α to its membrane receptor, TNFR. This activation triggers a cascade of other pro-inflammatory and pro-oxidant enzymes, such as NOX and iNOS, thereby increasing the generation of O_2_^•-^ and NO^•^ [18].

However, recent discoveries have shown that NF-κB activation in specific microenvironments, such as tumors and colitis, can lead to an antioxidant effect by recruiting M2 macrophages, which have anti-inflammatory properties, unlike M1 phenotype macrophages. Additionally, NF-κB activation mediated by ROS does not trigger the classical IL-6 synthesis pathway, further contributing to its anti-inflammatory role [19].

Therefore, it is evident that NF-κB has diverse involvements depending on the environment in which it is activated. This underscores the necessity of investigating this nuclear factor as a therapeutic target when testing new pharmacological or non-pharmacological interventions for the treatment of IBD.

Another strong connection between oxidative stress and inflammation is observed in the dysregulation of the mitochondrial electron transport chain in patients with IBD, which explains the elevated production of O_2_^•-^ in these individuals. Interestingly, this mitochondrial regulation is re-established following the use of TNF-α inhibitors. However, the mechanism underlying this interaction remains unclear and warrants further investigation [20]. 

RONS can disrupt tight junctions (TJ) and adherens junctions (AJ) in the intestinal epithelial intercellular junctions. Chronic inflammatory processes, resulting from epithelial integrity disruption, allow the migration of bacteria from the intestinal lumen to the internal environment, leading to the inflammatory histological changes characteristic of IBD [12,21]. 

With the infiltration of mucosal tissue by activated phagocytic immune cells generating RONS, a shift towards pro-oxidants occurs, disrupting cellular homeostasis and contributing to cellular injury and increased mucosal barrier permeability. This redox imbalance is closely connected to the activation of phagocytic immune cells during inflammatory processes characteristic of IBD. Lipopolysaccharides (LPSs) present in pathogenic intestinal bacteria, which increase during the dysbiosis occurring in IBD, are pathogen-associated molecular patterns (PAMPs) that activate enzymes generating O_2_^•-^ (NADPH oxidase) and NO^•^ (iNOS). Subsequently, this O_2_^•-^ is dismutated into H_2_O_2_, which can react with Fe^2+^ to generate ^•^OH or with Cl^-^ to produce hypochlorous acid (HClO), both of which have high antimicrobial activity [22]. Thus, reactive species are released from phagocytes into the extracellular environment, where they exert their antimicrobial function but can also contribute to tissue damage and increased mucosal barrier permeability [23]. This accelerates and perpetuates ongoing inflammation. Various genetic risk *loci* relevant to oxidative stress associated with IBD have been identified, further implicating oxidative stress in the pathogenesis of IBD [24].

**Figure 2:**
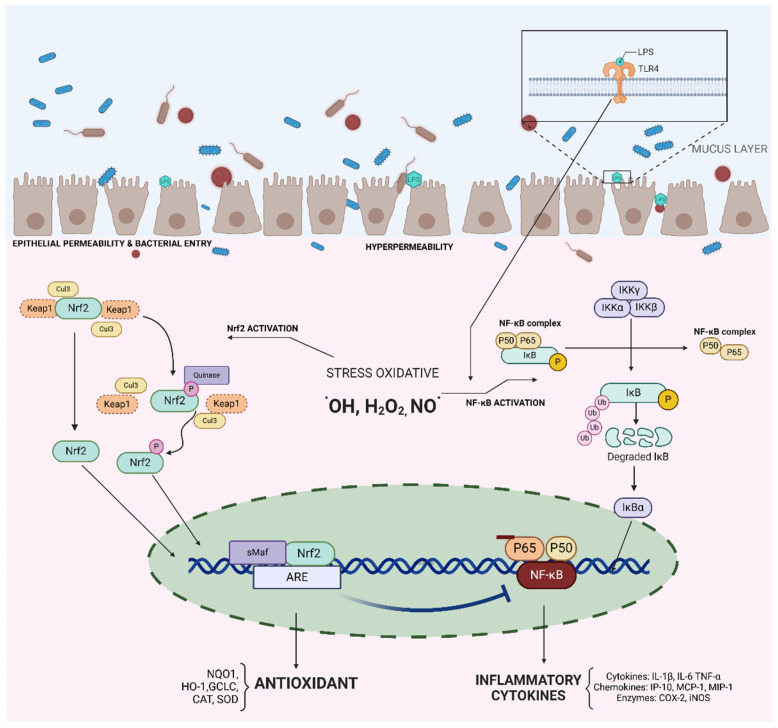
Interaction between nuclear factor erythroid 2 (Nrf2) and nuclear factor kappa B (NF-κB) in the modulation of the oxidative stress and inflammation Legend: Molecular mechanisms involved in the inflammatory and antioxidant response in intestinal epithelial cells, following recognition of bacterial LPS (lipopolysaccharide) by TLR4 (Toll-like receptor 4). TLR4 activation triggers two main pathways: (1) The Nrf2 pathway (nuclear factor erythroid 2-related factor 2), with the activation of the transcription of antioxidant genes, such as HO-1 (heme oxygenase-1), GCLC (glutamate-cysteine ligase catalase), CAT (catalase), and SOD (superoxide dismutase), which combat oxidative stress induced by reactive oxygen species (^•^OH, H_2_O_2_, and NO^•^). (2) NF-κB (nuclear factor kappa B) pathway: activation of the transcription of pro-inflammatory genes, including cytokines (IL-1β, IL-6, and TNF-α), chemokines (IP-10, MCP-1, and MIP-1), and enzymes (COX-2 and iNOS), which amplify the inflammatory response. The figure illustrates the complex interaction between the Nrf2 and NF-κB signaling pathways, which modulate the cellular response to oxidative stress and inflammation in the intestinal epithelium. The balance between these pathways is crucial for maintaining intestinal homeostasis and preventing tissue damage.

Nitro-oxidative stress (increased especially due to O_2_^•-^, H_2_O_2_, ^•^OH, HClO, ^•^NO, OONO^-^, N_2_O_3_, N_2_O_4_, and others) not only directly damages intestinal epithelial cells but also triggers pro-inflammatory pathways sensitive to reactive species in immune cells. The NF-κB and Nrf2 signaling pathways are two key transcription routes controlling numerous biological functions responding to oxidative stress and inflammation (Figure 2). NF-κB influences pro-inflammatory genes and cytokines and chemokines encoding, while Nrf2 induces antioxidants and detoxifying enzymes [25]. 

NF-κB is implicated in the pathogenesis of IBD due to dysregulation in its upstream signaling pathways. Immune receptors stimulating NF-κB, like nucleotide-binding oligomerization domain containing 2 (NOD2), and negative regulatory genes, such as IL-12 and IL-23, are found in the inflamed colonic tissues of IBD patients [15]. Conversely, Nrf2 downregulates pro-inflammatory responses and mucosal compromise through an antioxidant mechanism. Antioxidant-induced transcription can protect against the accumulation of overproduced reactive species. In oxidative stress, Nrf2 has been reported as a suppressor of protein kinase C and a diminisher of oxidase activation, increasing GSH levels [1]. 

**Figure 3:**
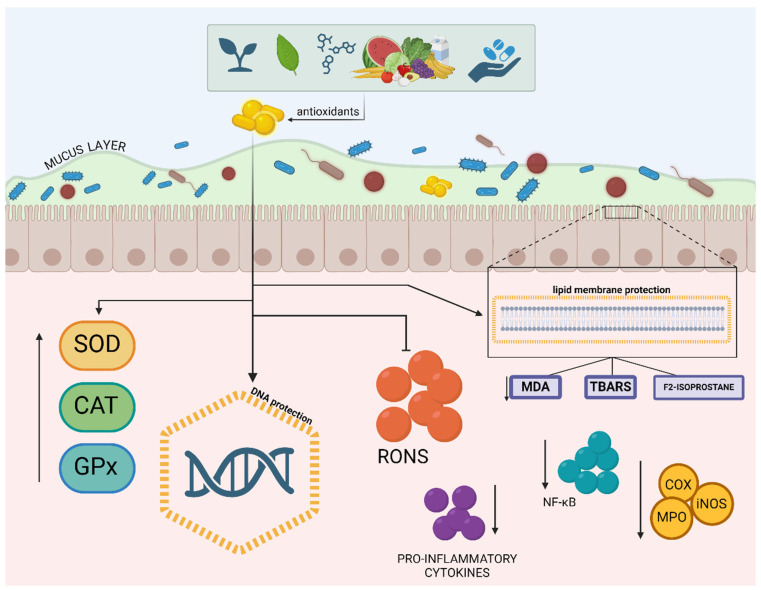
Mechanisms of action of antioxidants in the modulation of the intestinal inflammatory response. Legend: Mechanisms of antioxidant protection in the intestinal epithelium. Dietary and endogenously produced antioxidants neutralize reactive oxygen and nitrogen species (RONS), protecting deoxyribonucleic acid (DNA), membrane lipids (reducing malondialdehyde [351], thiobarbituric acid reactive substances [TBARS], and F-2 isoprostane), and mucus, increasing the production of the enzymes superoxide dismutase (SOD), catalase (CAT), and glutathione peroxidase (GPx), which also combat oxidative stress. Antioxidants attenuate effects of nuclear factor kappa B (NF-κB), reducing the production of pro-inflammatory cytokines and the expression of enzymes such as cyclooxygenase (COX), inducible nitric oxide synthase (iNOS), and myeloperoxidase (MPO), which can lead to tissue damage if left unchecked; ↑ (increased); ↓ (decreased).

In this study, we observed that some compounds had decreased NF-κB (n = 188) and increased Nrf2 expression (n = 55), as seen in Figure 3. However, in most studies, these markers are evaluated in isolation, making it challenging to interpret the interrelationship and impact of one on the other. This represents a significantly higher number than reported in the 2015 review, where only a few studies assessed these markers.

**Table 1:** General characteristics of the studies in animals and results: summary of the main characteristics of the included studies and presentation of the results of the studies, including the effects of the interventions on oxidative stress biomarkers.

Author	Compound	Antioxidant Action														
		↓RONS		↓ RONS Synthesis				↓ RONS Damage			Improved Antioxidant Defense					
		NO	RONS	iNOS	COX2 LPO NOX	MPO	NF-κB/ Iκ-Bα	MDA LP	PTN	DNA	SOD	CAT	GPx	GR GSH GST	Nrf2	TAC/ TAS
**Hormones**																
[26]	Melatonin															**X**
[27]	Dehydroepiandrosterone (DHEA)				**X**											
[28]	Obestatin					**X**		**X**			**X**	**X**				**X**
**Synthetic compounds**																
[29]	GL-V9 synthetic flavonoid			**X**		**X**		**X**			**X**			**X**		**X**
[30]	Glucose-lysine MRPs	**X**				**X**	**X**				**X**	**X**	**X**	**X**		**X**
[31]	ZnO nanoparticles (ZnONP) and ZnO microparticles (ZnOMP)		**X**			**X**								**X**	**X**	
[32]	Chromium-D-phenylalanine complex (Cr(D-phe)				**X**	**X**								**X**		
[33]	Hydrogen-rich water (HRW)										**X**	**X**		**X**		
[34]	P-chloro-phenylselene cholesterol (PCS)					**X**					**X**	**X**				
[35]	Selenium nanoparticles (ULP-SeNPs)			**X**	**X**	**X**	**X**						**X**	**X**		
[36]	Hydroxyproline			**X**		**X**	**X**	**X**			**X**	**X**		**X**		
[37]	LL202 (synthetic flavonoid)										**X**	**X**		**X**	**X**	
[38]	RSV imine (IRA), 2-methoxyl-3,6-dihydroxyl-IRA 3,4,5,4-tetramethoxystilbene (C33)														**X**	
[39]	FA-97 (synthetic phenolic compound)		**X**	**X**				**X**							**X**	
[40]	Unconjugated bilirubin (UCB)					**X**	**X**									
[41]	Taurine-loaded chitosan pectin nanoparticles (Tau-CS-PT-nps) and chitosan pectin nanoparticles (CS-PT-nps)					**X**								**X**		
[42]	Turmeric-derived nanoparticles						**X**									
[43]	(R,R)-BD-AcAc2					**X**	**X**	**X**			**X**			**X**		
[44]	Lawesson’s reagent										**X**			**X**		
[45]	Nano-selenium modified with Eucommia ulmoides polysaccharide					**X**		**X**			**X**	**X**	**X**	**X**		**X**
[46]	Galactosylated polymeric nanocargoes	**X**		**X**		**X**		**X**				**X**		**X**		
[47]	Edible nanoparticles similar to exosomes from *Portulaca oleracea* L.					**X**										
[48]	Ferulic acid	**X**		**X**	**X**	**X**										
[49]	Ferulic acid	**X**		**X**			**X**	**X**			**X**	**X**				**X**
[50]	Res-CDF (cross-linked organic cyclodextrin-metal structure encapsulating resveratrol)	**X**				**X**		**X**			**X**	**X**		**X**		
[51]	Lipoic acid and/or N-acetylcysteine		**X**			**X**		**X**						**X**		
**Chemical products derived from sources other than plants**																
[52]	Phycocyanin	**X**				**X**	**X**						**X**			
[53]	Lycopene					**X**	**X**	**X**			**X**	**X**			**X**	
[54]	Melitin				**X**		**X**				**X**			**X**		
[55]	Shrimp peptide (SP)												**X**	**X**		
[56]	Polysaccharopeptide from sanghuang mushroom							**X**								
[57]	Inosine			**X**	**X**	**X**	**X**	**X**			**X**		**X**			**X**
[58]	Sodium butyrate										**X**	**X**			**X**	
[59]	Sea conch peptides hydrolysate					**X**										
[60]	Astaxanthin					**X**					**X**			**X**		
[61]	Astaxanthin						**X**	**X**								
**Polyphenols and other natural active compounds from medicinal plants**																
[62]	*P. argentea* methanolic extract (PAME)							**X**			**X**	**X**				
[63]	*Pfaffia paniculata* extract (Brazilian ginseng)					**X**										
[64]	*American ginseng*														**X**	
[65]	Panaxynol (bioactive component of *American ginseng*)		**X**	**X**	**X**											
[66]	*Phyllanthus niruri* L. spray-dried extract					**X**										
[67]	Turmeric					**X**								**X**		
[68]	Grape pomace extract					**X**					**X**		**X**			
[69]	Grape seed proanthocyanidin extract						**X**									
[70]	Grape seed proanthocyanidin extract							**X**			**X**			**X**		
[71]	Isoliquiritigenin					**X**	**X**									
[72]	Isoquercitrin			**X**	**X**											
[73]	Quercitrin					**X**										
[74]	Apple peel polyphenols (dried apple peel)					**X**	**X**	**X**			**X**				**X**	
[75]	Concentrated apple extract (CAE)			**X**	**X**						**X**					
[76]	Luteolin			**X**				**X**			**X**		**X**		**X**	
[77]	Luteolin							**X**			**X**					**X**
[78]	Fisetin			**X**	**X**	**X**	**X**							**X**		
[79]	*Myrtus communis* hydroalcoholic extract or essential oil					**X**										
[80]	*Myrtus communis* subspecies communis extract	**X**				**X**		**X**						**X**		
[81]	Epicatechin	**X**				**X**	**X**	**X**			**X**			**X**		
[82]	Methyl gallate				**X**									**X**		
[83]	Ethanolic extract (etohe) and hexane phase (hexp) from the leaves of *Combretum duarteanum* (Cd)				**X**	**X**					**X**					
[84]	Carvacrol (5-isopropyl-2-methylphenol)					**X**		**X**			**X**	**X**	**X**			
[85]	Tuber of *Amorphophallus paeoniifolius* (Dennst.) *Nicolson* (Araceae)				**X**	**X**					**X**	**X**		**X**		
[86]	Rosmarinic acid			**X**	**X**		**X**									
[87]	P-Cymene (p-C) and rosmarinic acid (RA)			**X**	**X**	**X**	**X**	**X**			**X**			**X**		
[88]	Rosmarinic acid-loaded nanovesicles					**X**									**X**	
[89]	Moringa seed extract (*Moringa oleiferalam*)	**X**		**X**	**X**	**X**									**X**	
[90]	*Phoenix loureiroi Kunth* methanolic extracts					**X**						**X**		**X**		**X**
[91]	*Pongamia pinnata* (Karanja)	**X**				**X**		**X**			**X**	**X**		**X**		
[92]	*Averrhoa bilimbi* L. extract	**X**		**X**	**X**						**X**			**X**		
[93]	*Olea europaea leaf* extract			**X**	**X**											
[94]	Morusin										**X**	**X**				
[95]	Soy isoflavones						**X**				**X**		**X**			**X**
[96]	Geniposide			**X**	**X**	**X**	**X**									
[97]	Geniposide					**X**	**X**									
[98]	Geniposide						**X**	**X**						**X**	**X**	
[99]	Hyperoside (hyp)				**X**		**X**				**X**				**X**	
[100]	*Ocimum gratissimum leaves* polyphenol-rich extract	**X**			**X**	**X**					**X**	**X**		**X**		
[101]	*Ocimum gratissimum Linn*.					**X**								**X**		
[102]	*Sesbania grandiflora*	**X**				**X**		**X**			**X**			**X**		
[103]	Oligonol			**X**	**X**										**X**	**X**
[104]	Thymol					**X**	**X**									
[105]	Thymol	**X**			**X**	**X**	**X**									
[106]	Brazilian Berry (*Myrciaria jaboticaba*) peel aqueous extract			**X**		**X**					**X**	**X**	**X**	**X**		
[107]	Epigallocatechin gallate (EGCG)					**X**										
[108]	(-)-Epigallocatechin-3-gallate							**X**								
[109]	Epigallocatechin gallate (EGCG)						**X**									
[110]	*Carum copticum* L. Extract					**X**								**X**		
[111]	Hesperidin										**X**			**X**	**X**	
[112]	Hesperidin (HMC)						**X**							**X**		**X**
[113]	Hesperidin	**X**									**X**					**X**
[114]	Mother tincture (MT) from fresh, young, nonwoody *Thuja occidentalis* L.													**X**		
[115]	Phloretin						**X**									
[116]	Phloretin										**X**			**X**		
[117]	Curcumin-galactomannoside			**X**	**X**	**X**					**X**	**X**	**X**			
[118]	Curcumin						**X**									
[119]	Curcumin in hydroxyethyl starch microspheres					**X**										
[120]	Curcumin					**X**										
[121]	Honey polyphenols	**X**				**X**	**X**				**X**			**X**		**X**
[122]	*Glochidion ellipticum Wight* extracts			**X**	**X**		**X**				**X**	**X**				
[123]	Galangin						**X**							**X**		
[124]	Galangin					**X**										
[125]	Taxifolin						**X**				**X**					
[126]	*Flos lonicerae*					**X**	**X**	**X**			**X**					
[127]	Oroxindin						**X**									
[128]	Polyphenolic maqui extract (*Aristotelia chilensis*)			**X**	**X**										**X**	
[129]	Polyphenolic maqui extract (*Aristotelia chilensis*)						**X**									
[130]	Acacetin			**X**	**X**											
[131]	Flavonoid composition rich *P. Subpeltata Ortega*				**X**	**X**					**X**	**X**		**X**		
[132]	Juglone (JUG)						**X**						**X**		**X**	**X**
[133]	Hydroxytyrosol (HYT) from olive leaves extract (OLE)			**X**	**X**		**X**	**X**								
[134]	Lingonberry (LB)			**X**	**X**	**X**										
[135]	*Caragana sinica* extract					**X**					**X**	**X**		**X**		
[136]	*Quercus brantii* (QB) extract	**X**				**X**										
[137]	*T. Occidentalis leaf* extract (ato)	**X**				**X**	**X**			**X**	**X**			**X**		
[138]	*Dilodendron bipinnatum Radlk*. extract				**X**	**X**								**X**		
[139]	*Copaifera malmei leaf* infusion extract (iecm)					**X**		**X**						**X**		
[140]	Kaempferol (kae)						**X**									
[141]	6-Paradol from seeds of *Aframomum melegueta*					**X**		**X**						**X**		
[142]	*Maesa lanceolata* hydroethanolic extract	**X**									**X**			**X**		
[143]	*Garcinia mangostana* and *α-mangostin* extract	**X**	**X**			**X**		**X**			**X**					
[144]	*Garcinia pedunculata bark* (AEGP) aqueous extract				**X**						**X**	**X**		**X**		
[145]	Troxerutin				**X**	**X**					**X**	**X**	**X**		**X**	
[146]	*P. Lentiscus leaf* aqueous extract										**X**	**X**		**X**		
[147]	Wogonin			**X**	**X**	**X**	**X**								**X**	
[148]	Plant polyphenols (gallic acid, proanthocyanidin, ellagic acid, and tannic acid)														**X**	
[149]	Gallic acid										**X**	**X**		**X**	**X**	
[150]	Gallic acid						**X**									
[151]	Gallic acid					**X**										
[152]	Arum maculatum							**X**								
[153]	Coumaric acid and syringic acid														**X**	
[154]	Syringic acid			**X**			**X**									
[155]	Syringic acid	**X**		**X**	**X**											
[156]	Apigenin				**X**	**X**		**X**						**X**		
[157]	Safranal						**X**									
[158]	Resveratrol			**X**												
[159]	Resveratrol in polysaccharide-zein nanoparticles from Mesona chinensis					**X**										
[160]	Oxiresveratrol (OXY)			**X**	**X**	**X**										
[161]	Ligustroside										**X**					
[162]	Isoimperatorin					**X**		**X**								
[163]	Forsythia suspensa polyphenols			**X**												
[164]	m *Callicarpa nudiflora Hook* flavonoids						**X**									
[165]	*Calliandra haematocephala* extracts	**X**						**X**			**X**					
[166]	Diosmin						**X**	**X**			**X**			**X**	**X**	
[167]	*Ziziphus jujuba Mill* polyphenol extracts			**X**	**X**	**X**										
[168]	Geraniol			**X**	**X**	**X**	**X**				**X**			**X**		
[169]	*Terminalia catappa Linn*			**X**		**X**								**X**		
[170]	*L. Dentata* or *L. Stoechas*			**X**	**X**	**X**								**X**		
[171]	Demethyleneberberine—DMB (component from *Cortex Phellodendri Chinensis*)						**X**							**X**		
[172]	*Raphanus sativus* L. *seeds* aqueous extract			**X**		**X**	**X**			**X**						
[173]	Myrrh	**X**									**X**	**X**				
[174]	Gegen qinlian							**X**								
[175]	Ursolic acid					**X**	**X**	**X**			**X**					
[176]	*Lion’s Mane Medicinal Mushroom* and *Hericium erinaceus* (Agaricomycetes) ethanol extract	**X**				**X**					**X**					
[177]	Polysaccharide from cultured mycelium of *Hericium erinaceus*						**X**				**X**					
[178]	Polysaccharides from the edible mushroom *Hericium erinaceus*							**X**			**X**	**X**				
[179]	*Amphipterygium adstringens* extract										**X**		**X**			
[180]	Mangiferin					**X**		**X**			**X**	**X**		**X**		
[181]	*Portulaca oleracea* L.	**X**				**X**	**X**				**X**					
[182]	*Portulaca oleracea* L.					**X**		**X**			**X**	**X**		**X**		
[183]	Andrographolide				**X**	**X**	**X**									
[184]	*Carpolobia lutea G. Don* (Polygalaceae)					**X**					**X**			**X**		
[185]	*Lagerstroemia speciosa*										**X**	**X**		**X**		
[186]	*Atractylodes macrocephala* and *Taraxacum herba* extracts			**X**		**X**	**X**									
[187]	*Aronia berry*		**X**										**X**			
[188]	*Aronia berry* extract	**X**														
[189]	*Aronia melanocarpa* (Michx.) Elliott.	**X**		**X**		**X**	**X**								**X**	
[190]	Decursin and decursinol				**X**											
[191]	*Perilla frutescens* extract (PE)						**X**								**X**	
[192]	Glyceollins from daidzein in soybean	**X**		**X**	**X**		**X**			**X**						
[193]	Catalpol		**X**								**X**			**X**		
[194]	D-limonene			**X**	**X**		**X**				**X**			**X**		
[195]	Liriodendrin					**X**	**X**	**X**			**X**		**X**			
[196]	Yuzu (*Citrus junos Tanaka*)						**X**									
[197]	*Veronica polita*	**X**		**X**	**X**		**X**	**X**								
[198]	*Ziziphus spina-christi* fruit extract			**X**	**X**	**X**	**X**				**X**	**X**	**X**	**X**	**X**	
[199]	Red raspberries				**X**			**X**								
[200]	Plumieride					**X**					**X**	**X**		**X**		
[201]	*Ipomoea asarifolia* aqueous extract			**X**		**X**	**X**	**X**						**X**		
[202]	Osthole					**X**		**X**			**X**	**X**	**X**	**X**		
[203]	*Polygonum cuspidatum Siebold & Zucc* root Extract					**X**	**X**	**X**			**X**	**X**	**X**			
[204]	*Alpinia officinarum*				**X**	**X**	**X**				**X**	**X**	**X**	**X**		
[205]	Magnolol					**X**	**X**									
[206]	Magnolol contained in a butyrate-derived polymer nanoplatform					**X**										
[207]	*Trichilia catigua* ethyl-acetate fraction					**X**										
[208]	4-methylesculetin					**X**								**X**		
[209]	4-methylesculetin					**X**									**X**	
[210]	Fargesin	**X**			**X**	**X**	**X**									
[211]	Sinomenine			**X**							**X**				**X**	
[212]	Sinomenine	**X**		**X**	**X**	**X**	**X**	**X**			**X**	**X**	**X**		**X**	
[213]	Stevioside			**X**	**X**	**X**	**X**				**X**	**X**		**X**		
[214]	Sesamin										**X**			**X**	**X**	
[215]	*Terminalia arjuna* hydroalcoholic extract	**X**				**X**					**X**	**X**		**X**		
[216]	*Persea americana Mill*. Avocado extract			**X**	**X**	**X**	**X**									
[217]	Quercetin aglycone	**X**				**X**		**X**					**X**	**X**		
[218]	2-O-β-d-Glucopyranosyl-l-ascorbic acid, an ascorbic acid derivative isolated from the fruits of *Lycium Barbarum* L.			**X**	**X**											
[219]	Apocynin			**X**	**X**										**X**	
[220]	Apocynin					**X**		**X**			**X**	**X**		**X**		
[221]	Crocin										**X**			**X**	**X**	
[222]	Crocin	**X**										**X**				
[223]	Crocina	**X**					**X**									
[224]	Salidroside (salt)										**X**	**X**	**X**			
[225]	Freeze-dried fruit powder of *Actinidia arguta*					**X**					**X**			**X**		
[226]	*Tagetes erecta* L. flowers hydroalcoholic extract					**X**					**X**	**X**		**X**		
[227]	Daidzein					**X**	**X**									
[228]	*Ajuga chamaepitys* (L.) *Schreber* subsp. *Chia* (Schreber)					**X**										
[229]	*Sorbus domestica*				**X**	**X**		**X**		**X**	**X**	**X**				
[230]	*Bryophyllum pinnatum* (Lamarck) leaf extract			**X**	**X**	**X**	**X**							**X**		
[231]	*Piper umbellatum* L. (Piperaceae)	**X**				**X**		**X**			**X**	**X**		**X**		
[232]	Myristicin					**X**	**X**				**X**	**X**	**X**			
[233]	*Bruguiera gymnorrhiza leaves*			**X**	**X**		**X**	**X**								
[234]	Arjunarishta	**X**				**X**		**X**			**X**			**X**		
[235]	*Antrocaryon micraster*					**X**						**X**		**X**		
[236]	Piperine	**X**		**X**	**X**						**X**			**X**		
[237]	Puerarin	**X**		**X**	**X**	**X**	**X**				**X**	**X**		**X**	**X**	
[238]	*Rumex japonicus Houtt.*				**X**											
[239]	*Gloeostereum incarnatum*	**X**					**X**				**X**	**X**			**X**	
[240]	Sinapic acid					**X**					**X**	**X**	**X**	**X**		
[241]	Nerolidol			**X**	**X**	**X**					**X**	**X**			**X**	
[242]	Nerolidol (NRD)					**X**		**X**			**X**	**X**		**X**		
[243]	*Jasonia glutinosa* (L.) DC. extract	**X**		**X**	**X**	**X**										
[244]	*Sanhuang shu’ai*					**X**	**X**	**X**								
[245]	*Flammuliana velutipes* polysaccharide	**X**				**X**	**X**				**X**					
[246]	of the *Syringa oblata Lindl* Iridoid glycosides				**X**		**X**									
[247]	Mucilage Garden cress													**X**		
[248]	*Otostegia fruticosa leaves* crude extract											**X**		**X**		
[249]	*Dracocephalum kotschyi* methanol extract							**X**			**X**		**X**			
[250]	Fruit of *Rosa odorata doce var.gigantea*	**X**			**X**		**X**				**X**				**X**	
[251]	*Artemisia argyi* extract			**X**	**X**		**X**								**X**	
[252]	*Inula viscosa* ethanolic extract						**X**	**X**					**X**	**X**	**X**	
[253]	Cepharanthine						**X**	**X**						**X**	**X**	
[254]	*Saposhnikovia divaricata*				**X**		**X**									
[255]	*Trigonellafoenum-graecum* L. seeds aqueous extract							**X**			**X**	**X**		**X**		
[256]	Quinic acid	**X**		**X**			**X**	**X**			**X**	**X**				**X**
[257]	*Echinacea purpurea* extract												**X**			
[258]	*Echinacea purpurea* polysaccharide					**X**		**X**			**X**					**X**
[259]	Daphnetin							**X**			**X**					
[260]	Oxyberberin					**X**	**X**	**X**			**X**				**X**	
[261]	Curculigoside (CUR), from *Curculigo orchioides Gaertn*					**X**		**X**			**X**	**X**		**X**	**X**	
[262]	*Scrophularia striata Boiss* aqueous and hydroalcoholic Extracts					**X**		**X**								
[263]	*Picralima nitida seeds* crude alkaloidal extract	**X**				**X**		**X**						**X**		
[264]	*Mesua assamica* (King&Prain) *kosterm. Bark* ethanolic extract	**X**				**X**	**X**	**X**			**X**			**X**	**X**	
[265]	Higenamine					**X**	**X**									
[266]	Carboxymethyl Poria Polysaccharides					**X**					**X**					
[267]	1,8-cineol (eucaliptol)			**X**	**X**	**X**	**X**				**X**	**X**			**X**	
[268]	*Polygonatum Cyrtonema Hua* Oligosaccharides							**X**			**X**		**X**		**X**	
[269]	Polysaccharide from the fermented mycelium of *Inonotus obliquus*	**X**				**X**		**X**			**X**					
[270]	*Pinus eldarica* aqueous and hydroalcoholic extracts					**X**		**X**								
[271]	*Terminalia chebula* ethyl acetate extract						**X**	**X**			**X**	**X**		**X**		
[272]	*Ecklonia cava* extract			**X**	**X**											
[273]	*Salvia verbenaca* extract			**X**		**X**								**X**		
[274]	*Commiphora leptophloeos* extract			**X**	**X**		**X**	**X**								
[275]	*Sagittaria sagittifolia* L. polysaccharides					**X**	**X**	**X**			**X**					
[276]	*Tetrastigma hemsleyanum* root extract							**X**			**X**					
[277]	Gilaburu (*Viburnum opulus* L.) fruit extract	**X**				**X**		**X**						**X**		
[278]	Acidic polysaccharide from *Selaginella uncinata (Desv.) Spring*					**X**		**X**			**X**	**X**				
[279]	*Lizhong decoction*							**X**			**X**		**X**	**X**	**X**	
[280]	(-)-Syringaresinol				**X**											
[281]	Aegeline	**X**		**X**	**X**	**X**										
[282]	Fraxetin			**X**	**X**	**X**	**X**									
[283]	Honeysuckle			**X**	**X**											
[284]	*Passiflora edulis*			**X**												
[285]	Jinxiang garlic (*Allium sativum* L.)						**X**									
[286]	Cinnamaldehyde and hesperetin	**X**				**X**	**X**				**X**			**X**		
[287]	*Anacardium occidentale* L.	**X**		**X**		**X**	**X**									
[288]	Loganic acid	**X**					**X**	**X**			**X**			**X**	**X**	
[289]	Four sanshools of *Zanthoxylum fruit*							**X**			**X**	**X**		**X**		**X**
[290]	*Sea buckthorn*				**X**	**X**		**X**			**X**	**X**				**X**
**Functional foods and nutrients**																
[291]	Oat β-glucan					**X**		**X**								
[292]	Blueberry				**X**	**X**	**X**	**X**			**X**	**X**				
[293]	Riboflavin					**X**				**X**				**X**		
[294]	Goat whey			**X**			**X**									
[295]	Garlic oil					**X**		**X**			**X**			**X**		
[296]	Red bean			**X**		**X**								**X**		
[297]	Lecithin						**X**				**X**	**X**				
[298]	Coenzyme Q10				**X**	**X**						**X**		**X**		
[299]	Coenzyme Q10							**X**			**X**	**X**		**X**	**X**	
[300]	Honey			**X**							**X**			**X**		
[301]	Flaxseed extract				**X**	**X**		**X**			**X**	**X**	**X**	**X**		**X**
[302]	Β-glucans from *Lentinus edodes*	**X**		**X**		**X**		**X**								
[303]	Selenium				**X**	**X**										
[304]	Selenocysteine and selenocystine		**X**								**X**		**X**			
[305]	Selenium in biogenic nanoparticles							**X**			**X**		**X**			**X**
[306]	Camellia oil					**X**					**X**			**X**		
[307]	Walnut extract										**X**		**X**			
[308]	*Aqueous cinnamon* extract	**X**			**X**	**X**										**X**
[309]	Cinnamon (*Cinnamomum japonicum*) subcritical water extract					**X**										
[310]	Tocotrienol (alpha-tocopherol)	**X**			**X**	**X**	**X**	**X**								
[311]	Omega 3				**X**			**X**					**X**	**X**		
[312]	Eicosapentaenoic acid (EPA)						**X**	**X**			**X**			**X**		**X**
[313]	Noni juice-fortified yogurt		**X**													**X**
[314]	Isolated from fish skin gelatin hydrolysate (fsghf3)					**X**	**X**	**X**					**X**	**X**	**X**	
[315]	Mannoglucan (*Chinese yam.*)						**X**									
[316]	Alpha-tocopherylquinone														**X**	
[317]	Β-carotene						**X**									
[318]	Momordica charantia							**X**			**X**	**X**		**X**		
[319]	Virgin coconut oil	**X**				**X**										
[320]	Pumpkin polysaccharides						**X**									
[321]	Fermented yogurt					**X**										
**Probiotics**																
[322]	NTU 101; *L. rhamnosus* BCRC 16000; *L. paracasei* subsp. *paracasei* BCRC 14023										**X**	**X**	**X**	**X**		**X**
[323]	*Lactobacillus plantarum* (CAU1054 OR CAU1055, OR CAU1064) OR *Lactobacillus salivarius* CAU1301			**X**	**X**											
[324]	MegaSporeBiotic TM (MSB) probiotic capsules and MegaMucosa TM (MM) powder											**X**				**X**
[325]	*Bifidobacterium bifidum* ATCC 29521						**X**				**X**	**X**	**X**			
[326]	*Lactobacillus acidophilus* XY27				**X**	**X**	**X**	**X**			**X**	**X**				
[327]	Minas Frescal probiotic cheese containing *L. lactis*			**X**		**X**										
[328]	Probiotic yeast *Saccharomyces boulardii* (s. Boulardii)					**X**	**X**				**X**	**X**		**X**		
[329]	*Lactobacillus acidophilus* KDSL 1.0901, *Lactobacillus helveticus* KDSL 1.8701, *Lactobacillus plantarum* KDSL 1.0318, and mixed lactobacilli					**X**										
[330]	*Lactobacillus gasseri* 4M13					**X**					**X**	**X**	**X**			
[331]	*L. pentosus* A14-6 and *L. pentosus* CMY46				**X**											
[332]	*Lactobacillus acidophilus* C4										**X**	**X**		**X**		
[333]	*Exopolysaccharide Ropy Bifidobacterium pseudocatenulatum* Bi-OTA128							**X**			**X**	**X**		**X**		
**Others**																
[334]	Insect (cockroach) *Periplaneta americana*							**X**							**X**	
[335]	MicroRNAs					**X**		**X**			**X**	**X**		**X**	**X**	
[336]	Insect (cockroach) *Periplaneta americana*				**X**	**X**		**X**			**X**			**X**	**X**	
[337]	*Aspergillus awamori*	**X**				**X**					**X**		**X**		**X**	
[338]	Maggot extracts														**X**	
[339]	Meroterpene algae 11-hydroxy-1′-O—methylamadione			**X**	**X**	**X**										
[340]	Arthrospira (Spirulina) platensis	**X**				**X**										
[341]	hydroalcoholic extracts (HA) of cyanobacterium *Spirulina platensis*					**X**										
[342]	Chinese propolis					**X**		**X**			**X**			**X**		
[343]	*Saccharina japonica*							**X**			**X**					
[344]	*Aphanizomenon flos-aquae*	**X**		**X**	**X**	**X**	**X**									
[345]	Melanin from *Sepia pharaonis ink*						**X**	**X**		**X**	**X**					
[346]	Tuna bioactive peptides (TBP)										**X**		**X**			
[347]	Turtle peptide							**X**			**X**					**X**
[348]	Oxylipin-containing lyophilized biomass from a microalga			**X**	**X**		**X**								**X**	
[349]	Fermented Mekabu aqueous solution by *Lactobacillus plantarum Sanriku-SU7*		**X**													

Legend: CAT = catalase; COX2 = cyclooxigenase type 2; DNA = deoxyribonucleic acid; ERON = reactive oxygen and nitrogen species; GPx = glutathione peroxidase; GR = glutathione reductase; GSH = glutathione; GST = glutathione S-transferase; iNOS = inducible nitric oxide synthase; Iκ-Bα = nuclear factor of kappa light polypeptide gene enhancer in B-cells inhibitor, alpha; LP = lipid peroxidation; LPO = lipoxygenase; MDA = malondialdehyde; MPO = myeloperoxidase; NF-Κb = nuclear factor kappa-light-chain-enhancer of activated B cells; NO = nitric oxide; NOX = nicotinamide adenine dinucleotide phosphate-oxidase; Nrf2 = nuclear factor erythroid 2; PTN = protein; RONS = reactive oxygen and nitrogen species; SOD = superoxide dismutase; TAC = total antioxidant capacity.

However, as reported in the previous review [12], all studies found were in animal models primarily focusing on tissue expression, indicating a lack of human studies to corroborate findings from murine models.

Nrf2 is considered a critical factor in maintaining redox equilibrium, essential for expressing various antioxidant enzymes, providing direct protective action, and inducing cellular damage repair and tissue regeneration [17]. Thus, interest in modulating Nrf2 has been growing in recent years to maintain and restore human health [350].

Nevertheless, comprehending oxidative stress is not a straightforward task. The involvement of various molecules and cellular pathways, each responding to different stimuli depending on the tissue or compartment analyzed, complicates the evaluation of the antioxidant therapy. Therefore, it is essential to assess the specific targets of each tested compound. Only by understanding these targets can we safely prescribe combinations of different substances that act on distinct targets, thereby potentially enhancing therapeutic efficacy, whether used alone or in conjunction with combined therapy.

### 3.3. Action on Reactive Oxygen and Nitrogen Species and Their Generation

It is well known that RONS are produced both endogenously (mitochondria, peroxisomes, NOX, lipoxygenases, cytochrome P450, XO, COX, NOS, MPO, inflammatory cytokines, among others) and exogenously (ultraviolet light, radiation, chemotherapy, toxins, microorganisms, etc.). Low levels of these species impair various physiological functions, leading to issues in proliferative responses, immune system dysfunction, and defects in vasodilation. These alterations can result in conditions such as chronic granulomatous disease and certain autoimmune diseases [352].

In homeostasis, RONS are controlled by the enzymatic and non-enzymatic antioxidant defense system (eustress), which includes defenses naturally produced by the body, such as SOD, CAT, GPx, GR, thioredoxin (Trx), and peroxiredoxins. Additionally, certain metals, such as zinc and selenium, play crucial roles in antioxidant enzyme function, and natural chelators, including thiols, help neutralize metal ions that catalyze oxidative reactions. Dietary antioxidants like polyphenols, carotenoids, flavonoids, coenzyme Q10, vitamins C (ascorbic acid), and E (α-tocopherol) also contribute to this system [353]. Micromolecular antioxidants such as amino acids (e.g., cysteine, methionine, and glutamine) and other small molecules (e.g., uric acid and bilirubin) also play crucial roles in mitigating oxidative stress [354,355]. In a balanced state, redox signaling is essential. Under physiologic range (eustress or “good stress”), the reactive species generated play a critical role in microbial control by inhibiting the overgrowth of microbial populations, thus reducing microbial attacks. These reactive species aid in the programmed destruction of altered cells, contribute to embryogenesis, facilitate muscle exercise, and are crucial for the nervous system by enabling nerve impulse transmission between neurons [356].

However, in harmful situations (distress or “bad stress”), excessive release of RONS that overwhelms these defense mechanisms plays a crucial role in the development of diseases, aging, and carcinogenesis. This occurs when these antioxidant defenses fail [357]. Despite the ongoing discussion by Bienertova-Vasku and Scheringer (2020) [358] about the effectiveness of these concepts (eustress and distress) and suggesting that both are forms of oxidative stress to varying degrees, the concepts introduced by Selye in 1975 remain widely accepted and used [356].

Among the various animal models included in this review, oxidative stress/redox imbalance was present in both acute [30,35,36,48,78,89,91,92,96,215,221,311,359], and chronic situations [52,97,106,128,139], regardless of the colitis-inducing agent, whether acetic acid [117,202,249,301,303,306], dextran sodium sulfate [73,201,240,314,360], 2,4,6-trinitrobenzene sulfonic acid (TNBS) [136,183,207,236,361], among others. This indicates that oxidative stress needs to be controlled to minimize the effects of the disease, regardless of the phase, type, and severity of IBD.

The groups of “polyphenols and other active natural compounds derived from plants” (n = 242; 70.3%) were the most investigated classifications of antioxidants in animal models in this review. It was observed that the antioxidant effects of polyphenols involved the reduction of RONS concentration and synthesis, control of oxidative damage, improvement of antioxidant defenses, and effects on the intestinal microbiota, suggesting their broad action in controlling redox imbalance [67,117,118,362]. 

Among the compounds tested in animal models that demonstrated scavenging radical activity, particular attention should be given to those that act on reactive nitrogen species (RNS), reducing NO^•^ levels (n = 46; 13.3%). Noteworthy compounds include zinc oxide nanoparticles (ZnONPs) and zinc oxide microparticles (ZnOMPs) [31], galactosylated polymeric nanocarriers [46], phycocyanin [52], aphanizomenon flos-aquae algae [344], ferulic acid [48], *Myrtus communis* subspecies extract [80], epicatechin [81], and *Moringa oleifera* Lam seed extract [89], among others.

Antioxidant vegetal natural compounds, isolated or in mixtures, such as geraniol [168], *Vaccinium corymbosum* [292], *Ziziphus spina-christi* fruit extract [198], *Polygonum cuspidatum* root extract [203], and *Alpinia officinarum* [204], demonstrated a wide range of actions, including reduction of RONS, decreased synthesis of reactive species (inhibiting NOX and iNOS), and improved antioxidant defense by increase in levels of SOD, CAT, GPX, and GSH.

Some antioxidants’ mechanisms of action, which are based on this review, can be seen in Figure 2. It is important to note that reducing oxidative stress is crucial in situations where this stress is harmful or in cases of distress, such as in cardiovascular and inflammatory diseases. However, careful attention is needed when the treatment goal is to enhance oxidative stress, as is the case with antimicrobial actions or cancer treatment. Thus, it is essential to accurately assess when reducing RONS generation is a therapeutic target in a given clinical scenario.

### 3.4. Action on Antioxidant Defenses

Several studies confirmed reduced antioxidant defenses in both drug-induced colitis animal models [28,45,87,138,175,200,212,234,300,363] and in humans [363,364,365,366,367,368,369,370,371,372,373]. Among the 218 animal studies evaluating the action of compounds on antioxidant defenses, once again, the group of “polyphenols and other natural active compounds derived from plants” was the group studied that proved to be the most efficient in improving antioxidant defense (n = 151; 43.8%), with notable compounds, including gallic acid [149], *Ziziphus na-christi* fruit extract [198], plumieride [200], *Alpinia officinarum* [204], stevioside [213], sesamin [214], myristicin [232], sinapic acid [240], Lizhong decoction (a classical Chinese herbal) [279], and sinomenine [212], which showed beneficial effects on three or more biomarkers of antioxidant defenses. According to these findings, the markers SOD, CAT, and GSH were the most impacted by the antioxidants tested in the murine models of induced colitis.

In humans, as shown in Table 2 and Table 3, compounds such as omega-3 [363,364], pycnogenol [373], curcumin and piperine [366], spiruline [367], zinc aspartate [372], *Urtica dioica* leaf extract [368], resveratrol [369], and saffron [371] had effects on various antioxidant defenses, such as improvement in the antioxidant activity of CAT [363], a significant increase in the activity of SOD in whole blood, and an improvement in the antioxidant status of individuals [365]. TAS or TAC was also analyzed and increased with the use of vitamin D [374], spiruline [367], *Nigella sativa* [375], zingiber [376], *Pistacia lentisco* [377], and resveratrol [369]. However, it is essential to emphasize that these findings are challenging to compare due to the significant diversity in the methods used to evaluate them, which limits the ability to interpret the results collectively [378].

Several studies with induced colitis included in the systematic review (n = 55; 15.9%) observed a positive effect of the analyzed compounds, increasing Nrf2 activity, suggesting that the tested compounds could protect against the accumulation of overproduced reactive species. Notable compounds include hesperidin [111], troxerutin [145], luteolin [76], crocin [221], and American ginseng [64], among others.

### 3.5. Effect on Oxidative Damage

#### 3.5.1. Lipid Peroxidation (LP)

Inflammation, a hallmark of IBD, is closely linked to the generation of metabolites such as RONS, which contribute to nitro-oxidative damage present in the peripheral blood of IBD patients. These biomarker alterations occur as early as the onset of the disease, even in mild cases, and intensify as the disease progresses [388], particularly with the onset of severe complications, such as extraintestinal [389,390] manifestations (irreversible oxidative and inflammatory damage in the colon, liver, and kidney) and colorectal cancer (CRC) [17]. 

LP has emerged as a critical biological process resulting from nitro-oxidative stress as well as intestinal inflammation. Malondialdehyde (MDA) is one of the final products of lipid peroxidation and is the most measured metabolite in experimental and clinical research, especially in the form of thiobarbituric acid reactive substances (TBARS), evaluated in intestinal tissue. 

High levels of MDA have been detected in the intestinal tissues of IBD patients, indicating increased LP and, consequently, oxidative/inflammatory damage. In this context, MDA quantification can serve as an indicator of the degree of stress and damage associated with IBD and provide useful information for assessing the severity of the disease and the efficacy of antioxidant therapeutic interventions [391,392]. However, TBARS, a traditional method for evaluating LP, has some limitations, such as a lack of specificity, as they can react with other compounds, leading to a superestimation of oxidative damage. TBARS are highly unstable and easily degradable, making comparison between different studies difficult. Another limitation is the intake of foods rich in polyunsaturated fatty acids (PUFAs), which can influence TBARS levels regardless of inflammatory activity.

Another promising biomarker of lipid membrane damage is F2-isoprostane, a metabolite of prostaglandin F2α (PGF2α), generated non-enzymatically by the peroxidation of PUFAs. Its urinary and plasma concentrations are increased in IBD patients and directly correlate with disease severity and inflammatory activity. Studies point to the superiority of F2-isoprostane over TBARS as a biomarker of oxidative damage due to its greater specificity and stability [393,394,395]. 

In this review, we found 69 studies (20%) reporting a decrease in damage caused by RONS. All these articles measured MDA or TBARS and observed positive effects of various compounds in minimizing lipid peroxidation. Substances such as carvacrol (5-isopropyl-2-methylphenol) [84] and omega 3 [311], among others like garlic oil [295], oat β-glucan [291], mangiferin [180], *Veronica polita* [197], red raspberries [199], and osthole [202], were particularly effective in reducing lipid damage.

In human studies, 11 studies (45.8%) reported natural products present in so-called medicinal plants and their chemical compounds that reduced LP as indicated by decreased levels of MDA levels: zingiber [376], resveratrol [369], saffron [371], and oxidized LDL, using *Pistacia lentiscus* [377].

Isoprostane F2α type III (iPF2α-III or 15-F2t IsoP) can be measured in biological fluids and tissues. iPF2α-III is one of several urinary markers studied as potential predictors of IBD activity. Urinary excretion of iPF2α-III has been correlated with clinical relapse and inflammation in CD patients [396]. A study investigating urinary iPF2α-III concentrations as an index of LP in CD patients compared to healthy controls and tested whether LP correlates with clinical relapse and inflammation in patients showed elevated urinary iPF2α-III concentrations in CD patients. Those with clinical relapse had the highest levels, while patients in clinical remission had levels similar to controls, suggesting that increased LP is associated with clinical relapse [397].

The combination of vitamin E (800 IU) and vitamin C (1000 mg) daily demonstrated a similar baseline effect and significantly reduced plasma F2-isoprostane levels after 4 weeks of vitamin supplementation compared to the placebo group. The plasma LP product was significantly higher in the vitamin group at baseline. However, the change after 4 weeks was significantly more favorable in the vitamin group compared to the placebo group [379].

#### 3.5.2. Protein

As explained previously, the presence of RONS disrupts various proteins that constitute the mucosal epithelial barrier. These species can compromise TJ and AJ, which form the intercellular junctions of the intestinal epithelium, along with desmosomes. Epithelial TJs are known to form a barrier against allergens, toxins, and pathogens. The dephosphorylation of adherent proteins results in the breakdown of intercellular junctions, leading to barrier dysfunction and consequently increased intestinal permeability [12,21].

Carbonylated proteins, formed on various amino acid residues, such as arginine, histidine, lysine, proline, threonine, and cysteine, are the most widely used biomarkers for measuring protein oxidation. Due to their formation on multiple amino acid residues, carbonylation is more extensive and detectable compared to alterations on single amino acids, making it a critical marker for assessing oxidative damage in proteins [398]. Despite its importance, in this review, carbonylated proteins were not analyzed by the included studies, highlighting an unexplored gap in the research.

#### 3.5.3. DNA

RONS, such as H_2_O_2_, O_2_^-•^, NO^•^, and free oxygen radicals, can cause DNA damage in IBD through various mechanisms, contributing to inflammation progression, tissue injury, and complications associated with these conditions. RONS can induce structural alterations and conformational changes in DNA, which impair the integrity of the genetic material [399]. These species can cause lipid peroxidation, leading to disruptions in the cell membrane, which further exacerbate oxidative stress. Additionally, RONS decrease the efficiency of DNA polymerase and DNA repair enzymes by causing oxidative damage to proteins and genes involved in these processes. This can impede DNA replication and repair mechanisms (apoptosis and necrosis), leading to accumulated mutations. RONS also activate cytoplasmic and nuclear signal transduction pathways, stimulating cell proliferation [400]. Additionally, RONS can oxidize nitrogenous bases, producing metabolites like 8-hydroxy-2-deoxyguanosine (8-OHdG), a widely evaluated biomarker for detecting DNA strand breaks [401,402,403].

Compounds such as melanin from *Sepia pharaonis* ink [345], riboflavin [293], glyceollin [192], aqueous extract of *Raphanus sativus* L. seeds [172], and *Sorbus domestica* [229] were identified in this review as antioxidants with protective effects on DNA. However, all these compounds were tested exclusively in animal models, with no human studies available, highlighting a significant gap in the research on antioxidants in human IBD. 

### 3.6. Effect on Gut Microbiota

The role of the gut microbiota, comprising bacteria, viruses, fungi, and protozoa, in IBD has been the focus of extensive research over the years. However, defining and diagnosing dysbiosis remains a challenge, making the evaluation of the therapeutic properties of various substances an unresolved area of investigation [404]. 

The relationship between gut microbiota and oxidative stress is bidirectional. As discussed, the generation of reactive species without adequate neutralization, particularly ^•^OH, NO^•^, and ONOO^-^, causes significant damage to macromolecules, especially lipid membranes. This leads to the destruction of epithelial tissue and increased intestinal permeability, allowing bacteria and bacterial products, such as lipopolysaccharides (LPS) produced by Gram-negative bacteria, to penetrate the previously sterile layer of the intestine (lamina propria), further exacerbating inflammation and oxidative stress [405]. 

The entry of bacteria activates immune cells such as neutrophils and natural killer lymphocytes and increases the generation of pro-inflammatory cytokines like TNF-α, IL-22, IL-6, and IL-17, which perpetuate the oxidative cascade through NF-κB activation. Additionally, LPS from intestinal bacteria is recognized by toll-like receptor type 4 (TLR4), which, through myeloid differentiation factor 88 (MyD88), activates NF-κB and stimulates the mitochondrial electron transport chain. As a consequence, there is increased generation of O_2_^-•^, reduced antioxidant defense, and thus, an enhanced redox imbalance [406].

In this review, 29.9% (n = 103) of the studies (Table 4) evaluated the action of antioxidants on the intestinal microbiota in experimental models. One study evaluated the use of the hormone melatonin [26] in animals with DSS-induced colitis. The authors identified through intestinal microbiota taxonomy that the feces of these animals contained seven *phyla*, with Bacteroidetes (58.9%) being the most abundant in the diseased group, followed by Firmicutes (31.5%) and Proteobacteria (8.0%). In contrast, Firmicutes were the most abundant phyla in the melatonin-treated group (49.5%), followed by Bacteroidetes (41.6%) and Proteobacteria (7.5%). 

Similarly, Zhang and collaborators (2020) tested *Flammulina velutipes* polysaccharide (FVP) in preventing DSS-induced colitis and observed higher concentrations of Firmicutes, Bacteroidetes, and Proteobacteria in the diseased animals, which were restored to levels like healthy animals after FVP treatment.

Several studies included in this review demonstrated the effect of other active natural compounds from medicinal plants on the intestinal microbiota [70,116,148,258,269,306,329,407,408,409,410]. According to Xuan et al. (2020), it was observed that short-chain fatty acids (SCFAs) decreased after DSS-induced colitis, and treatment with galangin reversed these changes, increasing acetate and butyrate levels, which serve as essential fuel for colonocytes, thereby improving intestinal health. Additionally, galangin enriched specific bacterial populations that promoted SCFA production, such as *Butyricimonas* spp., mediated by its effects on intestinal microbiota remodeling.

**Table 4:** General characteristics of animal studies that investigated action on the intestinal microbiota.

Action on the Intestinal Microbiota		
Author	Components	Dose/Time
**Hormones**		
[26]	Melatonin	0.2 mg/L melatonin in water 7 days
**Synthetic compounds**		
[31]	ZnO nanoparticles (ZnONP) and ZnO microparticles (ZnOMP)	0.5, 5, and 50 mg/kg ZnONPs; 50 mg/kg of ZnOMPs 7 days
[43]	(R,R)-BD-AcAc2	Standard rodent food mixed with 4% (R,R)-BD-AcAc2 24 days
[45]	Nano-selenium modified with Eucommia ulmoides polysaccharide	200 µL 5 days
[47]	Edible nanoparticles similar to exosomes from *Portulaca oleracea* L.	20 mg/µL 5 days
**Chemical products derived from sources other than plants**		
[57]	Inosine	100 and 800 mg/kg 7 days
**Polyphenols and other natural active compounds from medicinal plants**		
[70]	Grape seed proanthocyanidin extract	50 mg/kg 21 days
[74]	Apple peel polyphenols (dried apple peel)	200 and 400 mg/kg 10 days
[87]	p-Cymene (p-C) and rosmarinic acid (RA)	25, 50, 100, and 200 mg/kg 48, 24, and 1 h before TNBS administration and 24 h after induction of inflammation.
[94]	Morusin	12, 5, 25, or 50 mg/kg 5 days
[106]	Brazilian Berry (*Myrciaria jaboticaba*) Peel (EJP) aqueous extract	Short-term EJP (weeks 6 and 7) OR Long-term EJP (weeks 2 to 7)
[115]	Phloretin	25, 50, and 100 mg/kg 7 days
[116]	Phloretin	60 mg/kg 10 days for colitis 17 days for microbiota
[118]	Curcumin	50 or 150 mg kg 7 days
[119]	Curcumin in hydroxyethyl starch microspheres	6.8 mg/kg 7 days
[121]	Honey polyphenols	10.5 mg/kg 7 days
[124]	Galangin	15 mg/kg 7 days
[125]	Taxifolin	100 mg/kg 14 days
[130]	Acacetin	50 and 150 mg/kg 9 days
[132]	Juglone (JUG)	0.04 mg/mL juglone 17 days
[140]	Kaempferol (Kae)	50 mg/kg/day 14 days
[148]	Plant polyphenols (gallic acid, proanthocyanidin, ellagic acid, and tannic acid)	100 mg/kg of each polyphenol 6 days
[158]	Resveratrol	100 mg/kg 9 days
[159]	Resveratrol in polysaccharide-zein nanoparticles from *Mesona chinensis*	10 mg/kg 14 days
[161]	Ligustroside	1, 2, and 4 mg/kg 7 days
[163]	Forsythia suspensa polyphenols	200, 400, and 600 mg/kg 7 days
[164]	Flavonoids from Callicarpa nudiflora Hook	400 mg/kg 17 days
[166]	Diosmin	100 and 200 mg/kg 7 days
[167]	Polyphenol extracts from *Ziziphus jujuba Mill*	200 mg/mL 7 days
[178]	Polysaccharides from the edible mushroom *Hericium erinaceus*	200, 300, and 400 mg/kg 7 days
[182]	*Portulaca oleracea* L.	400 and 800 mg/kg 8 days
[189]	*Aronia melanocarpa* (Michx.) Elliott.	100, 300, and 600 mg/kg 21 days
[199]	Red raspberries	6 g/kg 6 weeks
[201]	*Ipomoea asarifolia* aqueous extract	25, 50, and 100 mg/kg 3 days
[206]	Magnolol contained in a butyrate-derived polymer nanoplatform	5 mg/kg 2, 4, 12, and 24 h
[215]	*Terminalia arjuna* hydroalcoholic extract	125, 250, and 500 mg/kg 28 days
[217]	Quercetin aglycone (QUE)	QUE: 0.21%, QMQ: 0.36% 14 days
[218]	2-O-β-d-Glucopyranosyl-l-ascorbic acid, an ascorbic acid derivative isolated from the fruits of *Lycium Barbarum* L.	300 mg/kg 8 days
[233]	*Bruguiera gymnorrhiza leaves*	25, 50, and 100 mg/kg 7 days
[234]	Arjunarishta	1.8, 0.9, and 0.45 mL/kg 28 days
[235]	*Antrocaryon micraster*	30, 100, and 300 mg/kg 3 days
[237]	Puerarin	10 or 50 mg/kg 7 days
[238]	*Rumex japonicus Houtt*.	100 mg/kg 14 days
[240]	Sinapic acid	10 or 50 mg/kg 7 days
[244]	*Sanhuang shu’ai*	0.8 or 1.6 g/kg 7 days
[245]	*Flammuliana velutipes* Polysaccharide	50, 100, and 200 mg/kg 14 days
[253]	Cepharanthine	10 mg/kg 7 days
[254]	*Saposhnikovia divaricata*	50, 100, and 200 mg/kg 9 days
[258]	*Echinacea purpurea* polysaccharide	200 mg/kg 21 days
[266]	Carboxymethyl Poria polysaccharides	300 mg/kg/day
[268]	*Polygonatum Cyrtonema Hua* oligosaccharides	0.5, 2, and 5 mg/kg 5 days
[269]	Polysaccharide from the fermented mycelium of *Inonotus obliquus*	100, 200, and 400 mg/kg 7 days
[271]	*Terminalia chebula* ethyl acetate extract	100 and 200 mg/kg 7 days
[272]	*Ecklonia cava* extract	50, 100, 200, mg/kg 21 days
[275]	*Sagittaria sagittifolia* L. polysaccharides	100, 200, and 400 mg/kg 14 days
[276]	*Tetrastigma hemsleyanum* root extract	100 and 500 mg/kg 7 days
[278]	*Selaginella uncinata (Desv.) Spring* acidic polysaccharide	50 and 100 mg/kg 14 days
[282]	Fraxetin	10, 30, and 60 mg/kg 25 days
[283]	Honeysuckle	Extract added to the feed at 0.15, 0.75, and 1.5 g/kg 15 days
[284]	*Passiflora edulis*	8 mg/mL in drinking water 5 days
[289]	Four sanshools of *Zanthoxylum fruit*	2.5 mg/kg 7 days
[290]	*Sea buckthorn*	200 mg/kg 21 days
[341]	*Cyanobacterium Spirulina platensis* Hydroalcoholic extracts	100 or 200 mg/kg 15 days
[362]	Curcumin or resveratrol	Curcumin: 50 mg/kg or resveratrol: 80 mg/kg 7 days
[411]	Casein-quaternary chitosan complexes induced the soft assembly of egg white peptide and curcumin	15 mg/kg 7 days
[412]	Pc-Fe nanozyme (procyanidin and free iron)	100 mg/kg 7 days
[413]	Saffron	20 mg/kg 10 days
[360]	Crocetin	10 ou 40 mg/kg/dia 21 days
[414]	*Hydroxysaffor yellow A*	3 ou 6 mg/mL 7 days
[408]	Phlorizin	20, 40, and 80 mg/kg 7 days
[415]	Bay Laurel (*Laurus nobilis* L.)	Basal diet supplemented with 1 to 3% laurel 7 days
[416]	Okanin (*Coreopsis tinctoria Nutt*)	10 mg/kg 7 days
[417]	Alhagi honey Polysaccharide	200 and 400 mg/kg 7 days
[418]	Rhein (*Rheum rhabarbarum*)	50 mg/kg and 100 mg/kg 15 days
[419]	Schisandrin	20, 40, and 80 mg/kg 3 days before and 7 days after
[420]	*Hypericum sampsonii Hance*	3, 6, and 12 mg/kg 8 days
**Functional foods and nutrients**		
[285]	Jinxiang garlic (*Allium sativum* L.)	200 or 400 mg/kg/day 14 days
[305]	Selenium in biogenic nanoparticles	800 ng/kg 5 days
[306]	Camellia oil	2 mL/kg 20 days
[309]	Cinnamon (*Cinnamomum japonicum*) subcritical water extract	100, 300, or 500 mg/kg 21 days
[315]	Mannoglucan (*Chinese yam*)	300 mg/kg per day 7 days
[317]	β-carotene	50 mg/kg 7 days
[69]	Pumpkin polysaccharides	50 and 100 mg/kg 7 days
[421]	Quinoa	907 g/kg 7 days
[422]	α-tocopherol (αT) and tocopherol-rich γ-tocopheres (γTmT)	0.05% in the diet 21 days
[423]	Ornithine α-ketoglutarate	0.5%, 1.0%, and 1.5% 21 days
[424]	Sichuan pepper powder	Diet supplemented with 5% HJ powder 7 days
[425]	Ficus carica	150 and 300 mg/kg 35 days
**Probiotics**		
[325]	*Bifidobacterium bifidum* ATCC 29521	2 × 108 CFU/day 27 days
[329]	*Lactobacillus acidophilus* KDSL 1.0901, *Lactobacillus helveticus* KDSL 1.8701, *Lactobacillus plantarum* KDSL 1.0318, and mixed lactobacilli	1 × 109 CFU mL^−1^ 21 days
[330]	*Lactobacillus gasseri* 4M13	750 mg kg 14 days
[331]	*L. pentosus* A14-6 and *L. pentosus* CMY46	*L. pentosus* A14-6, *L. pentosus* CMY46, *L. pentosus* A14-6 plus XOS, and *L. pentosus* CMY46 plus GOS. 1 × 109 CFU/200 μL/day. 7 days
[332]	*Lactobacillus acidophilus* C4	1 × 109 CFU/mL 7 days
[333]	Exopolysaccharide Ropy *Bifidobacterium pseudocatenulatum* Bi-OTA128	2 × 109 CFU in 0.2 mL of saline 21 days
[426]	*Lactobacillus brevis* Bmb6	109 UFC in 100 µL of PBS 14 days
**Others**		
[334]	Insect (cockroach) *Periplaneta americana*	200 mg/kg and 100 mg/kg 7 days
[343]	*Saccharina japonica*	1, 2, and 4 g/kg 14 days
[345]	Melanin from *Sepia pharaonis ink*	75, 150, and 300 mg/kg 9 days
[346]	Tuna bioactive peptides	200 and 500 mg/kg 7 days
[347]	Turtle peptide	500 mg/kg 7 days
[427]	*Sargassum horneri*	100 mg/kg 4 semanas

Legend: CFU = colony-forming unit; GOS = galactooligosaccharides; TNBS = 2,4,6-trinitrobenzenesulfonic acid; XOS = xylooligosaccharides.

## 4. Conclusions and Perspectives

Compared to our group’s last review in 2015, there has been a notable increase in clinical trials assessing oxidative stress markers in human subjects. Despite remaining questions—such as the appropriateness of antioxidant therapy during the active phase, the molecular action of antioxidants in humans versus animal models, the optimal duration for supplementation, and the most suitable antioxidants for CD or UC—important advances have been made in understanding antioxidant therapy. Key findings include: (1) antioxidant action primarily enhances defenses such as SOD and TAS/TAC, protecting against lipid membrane oxidative damage; and (2) antioxidants appear to be safe and effective for nonhospitalized IBD patients, improving both oxidative stress and inflammation markers.

A critical challenge that remains is the absence of population reference values for oxidative stress markers, which hampers the interpretation of clinical results in human studies. Without established reference points for normality, it is difficult to determine whether antioxidant therapy successfully normalizes or brings these markers closer to normal levels following treatment.

Future research must prioritize testing antioxidant substances isolated or combined with pharmacological treatment in human subjects. While discovering new therapeutic substances is vital, the significant discrepancy between the number of substances tested in animal models and those tested in humans must be addressed. Promoting clinical research with rigorous methodological designs is crucial. This includes detailed sample preparation protocols, ensuring adequate sample sizes, and repeating experiments to verify reproducibility. In fact, it is essential to thoroughly evaluate the advantages and disadvantages of different analytical methods used for assessing nitro-oxidative stress and antioxidant efficacy and mainly consider chemical properties, sensitivity, specificity, and the context in which each method performs best. Furthermore, incorporating supplemental materials such as detailed protocols, technical videos, and instructional content in publications or laboratory reports would significantly contribute to advancing the quality and transparency of antioxidant research.

This responsibility lies with public and private research centers and scientific journals. Only through comprehensive, high-quality data can we conduct meta-analyses that will lead to recommendations by international societies, ensuring the prescription of these substances, alone or along with conventional therapies. Thus, despite the significant progress made in the past 9 years, we are still far from reaching a definitive answer regarding the prescription of antioxidants for individuals with IBD. More rigorous and well-structured clinical trials are necessary to establish clear guidelines and determine the real impact of antioxidant therapy in these patients.

## Figures and Tables

**Table 2:** General characteristics of the studies: summary of the main characteristics of the included studies.

Author	Ibd	Study	Intervention	Dose and Time of Intervention	Group Subjects, n and Age
**Functional foods and nutrients**					
[364]	UC mild or moderate phase	Blind randomized clinical trial	Omega-3	4.3 g (4800 mg—4 capsules of 1200 mg per day) 8 weeks	70 elderly Placebo: n = 35; age = 69.7 Intervention: n = 35; age = 69.7
[374]	UC mild or moderate phase	Randomized crossover clinical trial, placebo control	Omega-3	4.5 g/d (90 mg EPA + 60 mg DHA) 8 weeks	18 adults Placebo: n = 9; age = not informed intervention: n = 9; age = 40
[365]	CD in remission	Double-blind randomized controlled clinical trial	Antioxidant complex (AO) AO complex + omega 3 (n-3)	12 weeks	17 adults Placebo: n = 8; age = 38 AO: n = 8; age = 43 AO + omega 3: n = 9; age = 41
[379]	CD mild phase or remission	Double-blind randomized controlled clinical trial	Vitamin E + vitamin C supplement	Vitamin E (800 IU) Vitamin C (1000 mg) 4 weeks	57 adults Placebo: n = 29; age = 36.5 Intervention: n = 28; age = 38.3
[380]	Active CD	Double-blind randomized controlled clinical trial	Glutamine-enriched diet	Polymeric diet enriched with glutamine (42% of the amino acid composition) 4 weeks	15 children Placebo: n = 8; age = 10.5 Intervention: n = 7; age = 12.2
[381]	UC	Double-blind randomized controlled clinical trial	Intervention 1: ground flaxseed (GF) Intervention 2: Linseed oil (FO)	GF: 30,000 mg/d FO: 10,000 g/d 2 weeks	75 adults Placebo: n = 25; age = 35.2 GF: n = 25; age = 29.9 FO: n = 25; age = 32.2
[382]	UC mild or moderate phase	Randomized, multicenter, double-blind clinical study	Selenium	200 mcg/d 10 weeks	100 adults Placebo: n = 50; age = 37.9 Intervention: n = 50; age = 34.5
[374]	UC mild or moderate phase	Double-blind randomized controlled clinical trial	Vitamin D	Intervention 1: 1000 IU/d (I1) Intervention 2: 2000 IU (I2) 12 weeks	46 adults I1: n = 22; age = 39.7 I2: n = 24; age = 34
[383]	CD	Randomized controlled trial	Fish oil (EPA and DHA)	2.7 g/d 24 weeks	61 adults Placebo: n = 31; age = 40.5 Intervention: n = 31; age = 45.4
[384]	CD or UC	Prospective study	Riboflavin	100 mg/d 3 weeks	70 adults Group 1 (fecal Calprotectin < 200 µg/g): n = 40; age = 44.2 Group 2 (fecal calprotectin > 200 µg/g): n = 30; age = 38.8
[376]	UC mild or moderate phase	Double-blind randomized controlled clinical trial	Zingiber	2000 mg/d 12 weeks	46 adults Placebo: n = 24; age = 39.2 Intervention: n = 22; age = 41.4
[372]	Inactive to moderate CD or UC	Double-blind randomized controlled clinical trial	Zinc aspartate	300 mg 4 weeks	36 adults Placebo: n = 22; age = 38 Intervention: n = 14; age = 42
**Polyphenols and other natural active compounds from medicinal plants**					
[370]	UC mild or moderate phase	Randomized, double-blind, controlled pilot study	Resveratrol	500 mg/d 6 weeks	49 adults Placebo: n = 24; age = 39 Intervention: n = 25; age = 37.7
[369]	UC mild or moderate phase	Randomized, double-blind, controlled pilot study	Resveratrol	500 mg/d 6 weeks	56 adults Placebo: n = 28; age = 38.8 Intervention: n = 28; age = 37.4
[366]	Mild or moderate CD or UC	Double-blind randomized controlled clinical trial	Curcumin (doses of 1000 mg/day) or Curcumin + piperine	Curcumin: 1000 mg/day Piperine: 10 mg/day 12 weeks	Placebo: n = 19; 50.9 Curcumin: n = 20; 46.9 Curcumin + piperine: n = 19; 44.7 years
[385]	UC mild or moderate phase	Double-blind randomized controlled clinical trial	Saffron	100 mg 8 weeks	75 adults Placebo: n = 35; age = 40.97 Intervention: n = 40; age = 40.55
[371]	UC mild or moderate phase	Randomized controlled trial	Saffron	100 mg/d 8 weeks	75 adults Placebo: n = 35; age = 41.0 Intervention: n = 40; age = 40.5
[386]	CD mild or moderate stage	Pilot study	Mastic gum (*Pistacia lentiscus*)	0.37 g 4 weeks	18 adults Placebo: n = 8; age = 31.5 Intervention: n = 10; age = 36.9
[377]	CD or UC in remission	Double-blind randomized controlled clinical trial	*Pistacia lentiscus*	2800 mg/d 12 weeks	60 adults Placebo: n = 27; age = 45 Intervention: n = 33; age = 38.2
[373]	CD in remission	Pilot study	Pycnogenol	2 mg/d 12 weeks	29 teenagers Control: n = 15; age = 13.9 Intervention: n = 14; age = 16.3
[368]	Mild or moderate CD or UC	Double-blind randomized controlled clinical trial	*Urtica dioica leaf* extract	400 mg 12 weeks	59 adults Placebo: n = 29; age = 38.3 Intervention: n = 30; age = 36.6
[375]	UC mild or moderate phase	Double-blind randomized controlled clinical trial	*Nigella sativa*	2000 mg/d 6 weeks	48 adults Placebo: n = 24; age = 39.2 Intervention: n = 24; age = 34.8
[387]	CD or UC in remission	Randomized, single-blind, controlled study	Medicinal mushroom extract based on *Agaricus blazei Murill*	30 mL (twice a day) 3 weeks	50 adults Placebo: n = 25; age = not informed Intervention: n = 25: age = not informed
**Others**					
[367]	UC mild or moderate phase	Double-blind randomized controlled clinical trial	Spirulina	1 g/day (two 500 mg capsules/day) 8 weeks.	73 adults Placebo: n = 37; age = 39.5 Intervention: n = 36; age = 37.8

Legend: CD = Crohn’s disease; UC = ulcerative disease.

**Table 3:** Study results: presentation of the results of the studies, including the effects of the interventions on oxidative stress biomarkers and levels of pro- and anti-inflammatory cytokines.

Author	Intervention	Oxidative Stress Markers					Cytokines	General Effects
		SOD	GSH Complex	TAC	LP	OTHER		
**Functional Foods and Nutrients**								
[364]	Omega-3	↑			↓		↓ TNF-α, IL-2, IL-1α, and IL-1β	Improved serum levels of oxidative, antioxidant, and inflammatory markers, diastolic and systolic pressure, while AGE, MDA, Ox-LDL, catalase, superoxide dismutase, and TNF-α were altered in the control group
[363]	Omega-3	↑			↓	Cat: NS		No significant difference in laboratory indicators, sigmoidoscopy, or histology scores, and no significant difference in plasma MDA, erythrocyte LP, and CAT levels
[365]	Antioxidant complex (AO) AO complex + omega 3 (n-3)	↑				↑ total oxidant status		Increased serum concentrations of vitamin E, vitamin C, and SOD activity. TAS increased significantly after supplementation with AO
[379]	Vitamin E + vitamin C supplement				↓			Significant increase in their plasma levels compared to the placebo group.
[380]	Glutamine-enriched diet		↓					Did not change plasma concentrations of antioxidants
[381]	Intervention 1: ground flaxseed (GF) Intervention 2: linseed oil (FO)						↓ IL-6 and IFN-γ	Significant reduction in fecal calprotectin, Mayo score, ESR, INF-γ, IL-6, waist circumference, DBP, and systolic blood pressure, and a significant increase in TGF-β and IBDQ-9 score.
[382]	Selenium						↓ IL-17 IL-10: NS	Led to remission and improved quality of life. The concentration of IL-17 decreased. IL-10 levels did not show any considerable change between the two groups
[374]	Vitamin D			↑		↓ total oxidant status		Decreased TOS concentration in high doses, and increased IBDQ-9 scores compared to low-dose participants. However, no significant changes were observed in serum TAC or SCCAI scores.
[383]	Fish oil (EPA and DHA)						↓ TNF-α	Increased EPA and DHA incorporation into PBMCs, while reducing arachidonic acid incorporation and IFN production by mitogen-stimulated and lipopolysaccharide-stimulated PBMCs.
[384]	Riboflavin					↑ Free thiols	↓ IL-6, IL-10, IL-2, and TNF-α IL-1β: NS	Reduced oxidative stress and clinical symptoms. Decreased Enterobacteriaceae abundance, despite no evident changes in the fecal microbiome.
[376]	Zingiber			↑	↓			Improved disease severity index and oxidative stress
[372]	Zinc aspartate	NS						No changes were found in the plasma and erythrocyte metallothionein
**Polyphenols and other natural active compounds from medicinal plants**								
[370]	Resveratrol					↓ NF-κB	↓ TNF-α	Inflammatory markers can be reduced through attenuation of NF-kB activity, potentially improving quality of life.
[369]	Resveratrol	↑		↑	↓			Improve quality of life by reducing oxidative stress.
[366]	Curcumin (doses of 1000 mg/day) or curcumin + piperine	↑			↓	MPO, CAT and H2O2: NS	TNF-α, IL-17A, IL-22, and IL-10	Significantly increased SOD levels compared to a placebo group. However, the three-month treatment time did not significantly alter other biomarkers.
[385]	Saffron						↓ TNFα and IL17 ↑ IL10:	Significant decreases in serum TNF, hs-CRP, and IL-10 levels compared to the placebo group, while no significant difference was found in ESR, IL-17, and IBDQ-9 scores.
[371]	Saffron	↑		↑	↓			Reduced disease activity and increased serum levels of TAC, SOD, and GPX.
[386]	Mastic gum (*Pistacia lentiscus*)					↓ TNF-α	↓ IL-6	Significant decreases in CDAI, plasma IL-6, and CRP, and increase in TAP, with no side effects observed.
[377]	*Pistacia lentiscus*				↓	↓ Ox-LDL		Decreased oxLDL/HDL, oxLDL/LDL, and oxLDL/LDL
[373]	Pycnogenol	↑			↓			Serum AOC negatively correlated with disease activity and with CRP and fecal calprotectin
[368]	*Urtica dioica leaf* extract	↑						Significantly reduced hs-CRP levels, increased SOD levels, and increased scores on the IBDQ-9 questionnaire
[375]	*Nigella sativa*			↑	↓	NFκB: NS	TNF-α: NS	No significant differences in serum antioxidant capacity, NF-kB levels, or scores of IBDQ-9.
[387]	Medicinal mushroom extract based on *Agaricus blazei Murill*						↑ IL-1ß, IL-2, IL-4, IL-5, IL-7, IL-10, IL-13, IL-17, and TNF-α ↓ IL-6, IL-8: ↓ IL-12, and IFN-γ	Influenced cytokine levels and weak systemic anti-inflammatory effect
**Others**								
[367]	Spirulina	NS		↑	↓			Serum TAC levels increased, leading to a higher health-related quality of life score, but no significant changes in disease activity score.

Legend: CAT = catalase; IFN-γ = interferon-gamma; IL-1β = interleukin-1 beta; IL-2 = interleukin-2; IL-4 = interleukin-4; IL-6 = interleukin-6; IL-7 = interleukin-7; IL-8 = interleukin-8; IL-10 = interleukin-10; IL-12 = interleukin-12; IL-13 = interleukin-13; IL-17 = interleukin-17; IL-17 = interleukin-17A; MPO = myeloperoxidase; NFκB = nuclear factor kappa-light-chain-enhancer of activated B cells; NS = not significant; Ox-LDL = oxidized LDL; TNF-α = tumor necrosis factor alpha.

## Data Availability

This is an unpublished study that is not undergoing the submission process in any other scientific journal. All data are privately accessible.

## Supplementary Materials

The following supporting information can be downloaded at: https://www.mdpi.com/article/10.3390/antiox13111369/s1, Figure S1A: Risk of bias graph: review authors’ judgements about each risk of bias item presented as percentages across all included animal studies; Figure S1B: Risk of bias graph: review authors’ judgements about each risk of bias item presented as percentages across all included randomized clinical trials studies; Table S1. Dose and time of oral supplementation of the studies in animals.

## List of Abbreviations

5-ASA—5-aminosalicylic acid; ACG—American College of Gastroenterology; AJ—Adherens Junctions; CAM—Complementary and Alternative Medicine; CAT—catalase; CD—Crohn’s disease; CFU—colony-forming unit; COX—cyclooxygenase; CU—ulcerative colitis; DHEA—dehydroepiandrosterone; DNA—deoxyribonucleic acid; GIT—gastrointestinal tract; GOS—galactooligosaccharides; GPx—glutathione peroxidase; GR—glutathione reduced; GSH—reduced glutathione; GSSG—oxidized glutathione; IBD—inflammatory bowel diseases; IL—interleukin; INF-γ—interferon gamma; iNOS—inducible nitric oxide synthase; IκBα—nuclear factor of kappa light polypeptide gene enhancer in B-cells inhibitor, alpha; JAK—Janus kinase inhibitors; LOX—lipoxygenases; MAPK—mitogen-activated protein kinase; MDA—malondialdehyde; MPO—myeloperoxidase; MTX—methotrexate; MZ—mesalazine; NAC—N-acetylcysteine; NF-κB—nuclear factor kappa B; NOD2—nucleotide-binding oligomerization domain containing 2; NOS—inducible nitric oxide synthase isoforms; NOX—nicotinamide adenine dinucleotide phosphate oxidase; Nrf2—nuclear factor erythroid 2-related factor; PUFA—polyunsaturated fatty acids; RNS—reactive nitrogen species; RONS—reactive oxygen and nitrogen species; ROS—reactive oxygen species; SCFA—short-chain fatty acid(s); SNPs—single nucleotide polymorphisms; SOD—superoxide dismutase; SSZ—sulfasalazine; TAC—total antioxidant capacity; TAS—total antioxidant status; TBARS—thiobarbituric acid reactive substances; TJ—tight junctions; Trx—thioredoxin; TNBS—2,4,6-trinitrobenzenesulfonic acid; TNF-α—tumor necrosis factor alpha; TNFR—TNF-α receptor; TPs—thiopurines; XO—xanthine oxidase; XOS—xylooligosaccharides.
