# Supplementary material for: Antioxidant Therapy in Inflammatory Bowel Diseases: How Far Have We Come and How Close Are We?
Source: Antioxidants (Basel). 2024 Nov 8;13(11):1369. doi: 10.3390/antiox13111369 (PMC11590966; doi:10.3390/antiox13111369)
Supplement: Supplementary file 1 [file antioxidants-13-01369-s001.zip › antioxidants-3271551-supplementary.pdf]

Antioxidant Therapy in Inflammatory Bowel Diseases: How Far Have We Come and How Close Are We?

Lylian Ellen Militão dos Santos Xavier [1], Thays Cristhyna Guimaraes Reis [2], Amylly Sanuelly da Paz Martins [3], Juliana Célia de Farias Santos [4], Nassib Bezerra Bueno [1], Marília Oliveira Fonseca Goulart [3,5,*] and Fabiana Andréa Moura [1,4,*]

[1] Postgraduate Degree in Nutrition (PPGNUT), Federal University of Alagoas (UFAL), Maceió 57072-970, AL, Brazil; lylian.santos@fanut.ufal.br (L.E.M.d.S.X.); nassib.bueno@fanut.ufal.br (N.B.B.)
[2] Faculty of Nutrition (FANUT), Federal University of Alagoas (UFAL), Maceió 57072-970, AL, Brazil; thays.reis@fanut.ufal.br
[3] Postgraduate Studies at the Northeast Biotechnology Network (RENORBIO), Federal University of Alagoas (UFAL), Maceió 57072-970, AL, Brazil; amylly.martins@iqb.ufal.br
[4] Postgraduate Degree in Medical Sciences (PPGCM/UFAL), Federal University of Alagoas (UFAL), Maceió 57072-970, AL, Brazil; juliana.santos@fanut.ufal.br
[5] Institute of Chemistry and Biotechnology (IQB/UFAL), Federal University of Alagoas (UFAL), Maceió 57072-970, AL, Brazil
* Correspondence: mofg@qui.ufal.br (M.O.F.G.); fabiana.moura@fanut.ufal.br (F.A.M.)

Figure S1A. Risk of bias graph: review authors' judgements about each risk of bias item presented as percentages across all included animal studies

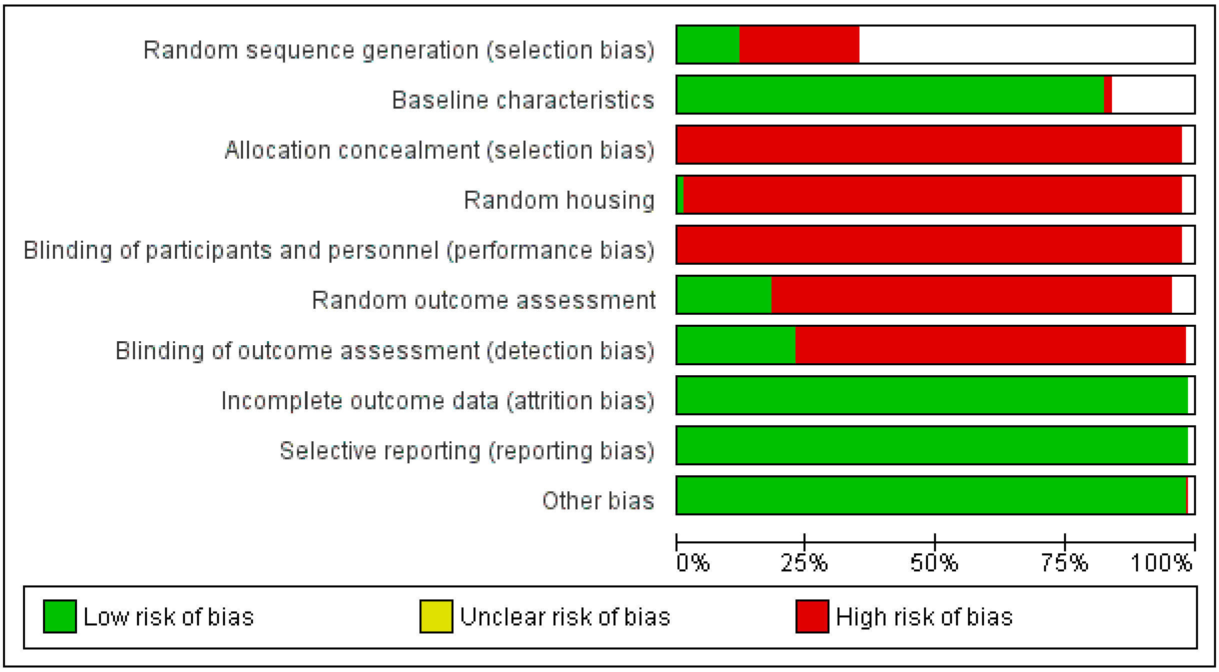

Figure S1B. Risk of bias graph: review authors' judgements about each risk of bias item presented as percentages across all included randomized clinical trials studies

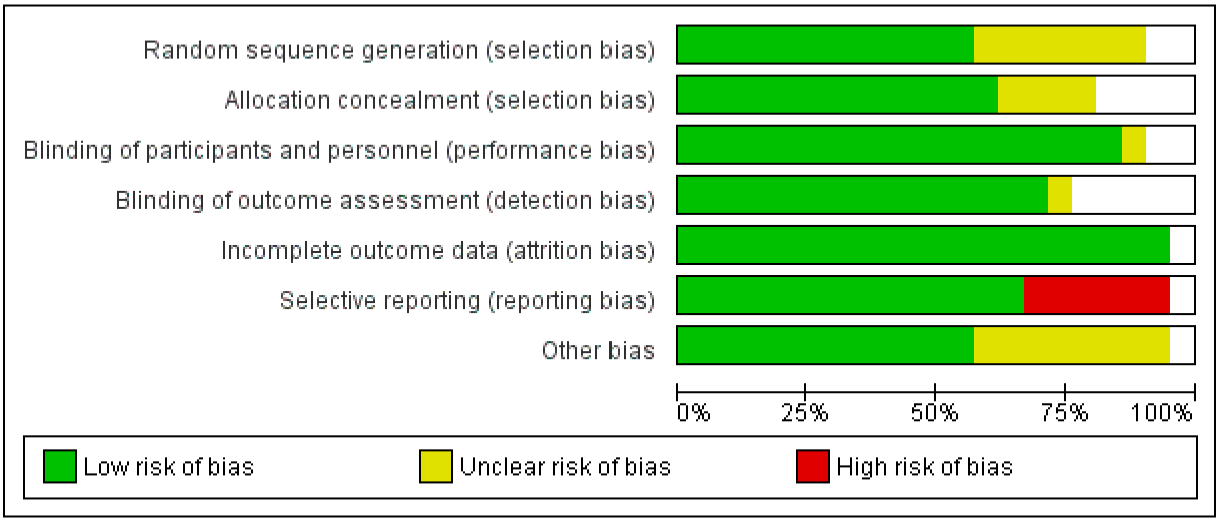

Table S1. Dose and time of oral supplementation of the Studies in animals

Author	Compound	Dose/time
	**Hormones**	
[26]	Melatonin	0.2 mg/L melatonin in water<br>7 days
[27]	Dehydroepiandrosterone (DHEA)	50mg/kg<br>10 days
[28]	Obestatin	16 nmol/kg<br>twice a day for 4 consecutive days
	**Synthetic compounds**	
[29]	GL-V9<br><br>synthetic flavonoid	12.5, 25 or 50 mg/kg 10 days
[30]	Glucose-lysine MRPs	210 or 2100 mg/kg<br>7 days
[31]	ZnO nanoparticles (ZnONP) and ZnO microparticles (ZnOMP)	0.5, 5, 50 mg/kg ZnONPs; 50 mg/kg of ZnOMPs<br>7 days
[32]	Chromium-D-phenylalanine complex (Cr(D-phe)	30, 60 and 90 mg/kg<br>11 days
[33]	Hydrogen-rich water (HRW)	5mL/kg<br>7 days
[34]	P-chloro-phenylselene cholesterol (PCS)	10mL/kg<br>9 days
[35]	Selenium nanoparticles (ULP-SeNPs)	0.8ppm<br>11 days
[36]	Hydroxyproline	1% Hyp (g/100 g)<br>6 days

Author	Compound	Dose/time
[37]	LL202 (synthetic flavonoid)	7.5, 15 and 30 mg/kg<br>3 days
[38]	RSV imine (IRA), 2-methoxyl-3,6-dihydroxyl-IRA 3,4,5,4-tetramethoxystilbene (C33)	200 mg/kg<br>9 days
[39]	FA-97 (synthetic phenolic compound)	2.5, 5 and 10 mg/kg<br>14 days
[40]	Unconjugated bilirubin (UCB)	0.2mL
[41]	Taurine-loaded chitosan pectin nanoparticles (Tau-CS-PT-nps) and chitosan pectin nanoparticles (CS-PT-nps)	taurine (500 mg/kg), pectin (300 mg/kg)<br>6 days
[42]	Turmeric-derived nanoparticles	3 mg/dose<br>7 days
[43]	(R,R)-BD-AcAc2	standard rodent food mixed with 4% (R,R)-BD-AcAc2<br>24 days
[44]	Lawesson's reagent	18.75 μM/kg
[45]	Nano-selenium modified with Eucommia ulmoides polysaccharide	200 μL<br>5 days
[46]	Galactosylated polymeric nanocargoes	5 mg/kg<br>7 days
[47]	Edible nanoparticles similar to exosomes from portulaca oleracea L	20 mg/μL<br>5 days
[48]	Ferulic acid	10, 20 and 40 mg/kg<br>14 days
[49]	Ferulic acid	20, 40 and 60 mg/kg<br>5 days
[50]	Res-CDF<br>(cross-linked organic cyclodextrin-metal structure encapsulating resveratrol)	100 mg/kg/d<br>21 days
[51]	Lipoic acid and/or N-acetylcysteine	LA: 100 mg/kg NAC: 100 mg/kg or LA + NAC: 100 mg/kg/d, each<br>7 days before mild colitis induction
**Chemical products derived from sources other than plants**		
[52]	Phycocyanin	150 mg<br>7 days
[53]	Lycopene	5, 10, 20 mg/kg<br>21 days
[54]	Melitin	40 μg/kg<br>5 days
[55]	Shrimp peptide (SP)	300 and 600 mg/kg<br>22 days
[56]	Polysaccharopeptide from sanghuang mushroom	200mg/kg<br>21 days
[57]	Inosine	100 and 800 mg/kg<br>7 days
[58]	Sodium butyrate	0.066 g/kg<br>7 days
[59]	Sea conch peptides hydrolysate	100, 200 and 400 mg/kg<br>15 days

Author	Compound	Dose/time
[60]	Astaxanthin	20 g/kg<br>14 days
[61]	Astaxanthin	0.04%<br>7 days before DSS administration and 3 days after
**Polyphenols and other natural active compounds from medicinal plants**		
[62]	*P. argentea* methanolic extract (PAME)	50, 100, 200 and 500 mg/kg<br>5 days
[63]	*Pfaffia paniculata* extract (Brazilian ginseng)	25, 50, 100, 200 and 400 mg/kg<br>Preventative protocol: 14 days before induction<br>Curative protocol: 7 days
[64]	*American ginseng*	AG: 75 mg/kg<br>HAG: 75 mg/kg<br>or PA: 1 mg/kg<br>14 days
[65]	Panaxynol (bioactive component of *American ginseng*)	0.01 mg/kg, 0.1 mg/kg, 0.5 mg/kg and 1 mg/kg<br>14 days
[66]	*Phyllanthus niruri L* spray-dried extract	25, 100 and 200 mg/kg<br>3 days before colitis induction + 1 day after
[67]	Turmeric	1, 10 or 100 mg/kg<br>3 days before or 7 days after induction
[68]	Grape pomace extract	semi-synthetic diet enriched with GPE (0.1%, 0.5% and 1%)<br>21 days
[69]	Grape seed proanthocyanidin extract	50 and 100 mg/kg<br>12 days
[70]	Grape seed proanthocyanidin extract	50 mg/kg<br>21 days
[71]	Isoliquiritigenin	30 mg/kg isoLQ (dissolved in 0.5% carboxymethylcellulose)<br>3 days
[72]	Isoquercitrin	1 and 10mg/kg<br>14 days
[73]	Quercitrin	1 and 5 mg/kg<br>10 days
[74]	Apple peel polyphenols (dried apple peel)	200 and 400 mg/kg<br>10 days
[75]	Concentrated apple extract (CAE)	1mL of CAE (4.37mg of polyphenol/day)<br>8 or 14 days
[76]	Luteolin	5, 20, 50 mg/kg<br>7 days
[77]	Luteolin	34.6 mg/kg/day<br>14 days
[78]	Fisetin	5 and 10 mg/kg of body weight<br>8 days
[79]	*Myrtus communis* hydroalcoholic extract or essential oil	MCHE: 50, 100, 200 and 400 mg/kg<br>MCEO: 62.5, 125, 250, and 500 µL/kg<br>4 days

Author	Compound	Dose/time
[80]	*Myrtus communis* subspecies communis extract	150, 1100, 1500 mg/kg<br>3 days
[81]	Epicatechin	200 and 300 mg/kg<br>7 days
[82]	Methyl gallate	100 and 300 mg/kg<br>5 days
[83]	Ethanolic extract (etohe) and hexane phase (hexp) from the leaves of *Combretum duarteanum* (Cd)	ACUTE PHASE: Cd -EtOHE or Cd -HexP (31.25, 62.5, 125, 250 mg/kg)<br>CHRONIC PHASE: Cd -EtOHE or Cd - HexP (31.25, 62.5, 125, 250 mg/kg).<br>48, 24 and 1h before induction and 24h after induction
[84]	Carvacrol (5-isopropyl-2-methylphenol)	25, 50 and 100 mg/kg<br>3 days
[85]	Tuber of *Amorphophallus paeoniifolius* (Dennst.) *Nicolson* (Araceae)	APME or APAE (250 and 500 mg/kg)<br>7 days
[86]	Rosmarinic acid	30 or 60mg/kg<br>7 days
[87]	P-Cymene (p-C) and rosmarinic acid (RA)	25, 50, 100 and 200 mg/kg<br>48, 24 and 1 h before TNBS administration and 24 h after induction of inflammation.
[88]	Rosmarinic acid-loaded nanovesicles	RA-loaded nanovesicles (5, 10 and 20 mg/kg) or free RA (20 mg/kg) during days 1, 3, 5 and 7 of DSS administration
[89]	Moringa seed extract (*Moringa oleiferalam*)	150 mg/kg<br>7 days
[90]	*Phoenix loureiroi Kunth* methanolic extracts	50, 100 and 200 mg/kg<br>Preventative: 11 days<br>Dressing: 14 days
[91]	*Pongamia pinnata* (Karanja)	100 and 200 mg<br>7 days
[92]	*Averrhoa bilimbi L.* extract	50 and 100 mg/kg<br>6 days
[93]	*Olea europaea leaf* extract	0.5, 1 and 10 mg/kg<br>11 days
[94]	Morusin	12.5, 25 or 50 mg/kg<br>5 days
[95]	Soy isoflavones	0.5%<br>7 days
[96]	Geniposide	25 or 50 mg/kg<br>14 days
[97]	Geniposide	50, 100, 200 mg/kg<br>3 days before + 5 days after
[98]	Geniposide	20 and 60 mg/kg<br>14 days
[99]	Hyperoside (hyp)	80 and 120 mg/kg<br>14 days
[100]	*Ocimum gratissimum leaves* polyphenol-rich extract	200, 400 and 800 mg/kg<br>7 days

Author	Compound	Dose/time
[101]	*Ocimum gratissimum Linn.*	50, 100, 200 and 400 mg/kg<br>5 days
[102]	*Sesbania grandiflora*	100 and 200 mg/kg<br>7 days
[103]	Oligonol	10, 50, and 100 mg/kg<br>7 days
[104]	Thymol	10, 30 and 100 mg/day<br>5 days
[105]	Thymol	100m/kg
[106]	Brazilian Berry (*Myrciaria jaboticaba*) peel aqueous extract	Short-term EJP (weeks 6 and 7)<br>OR<br>Long-term EJP (weeks 2 to 7)
[107]	Epigallocatechin gallate (EGCG)	20 and 50 mg/kg<br>7 days
[108]	(-)-Epigallocatechin-3-gallate	3.2 mg/mL<br>10 days
[109]	Epigallocatechin gallate (EGCG)	50 mg/kg/d<br>10 days
[110]	*Carum copticum L.* Extract	100, 200 and 400 mg/kg<br>7 days
[111]	Hesperidin	10, 20 and 40 mg/kg<br>13 days
[112]	Hesperidin (HMC)	10, 30 or 100 mg/kg<br>20 hours (inflammatory parameters)<br>10am (oxidative stress)
[113]	Hesperidin	50mg/kg<br>14 days
[114]	Mother tincture (MT) from fresh, young, nonwoody *Thuja occidentalis L.*	5, 25 and 50 mg MT/kg<br>7 days
[115]	Phloretin	25, 50 and 100 mg/kg<br>7 days
[116]	Phloretin	60mg/kg<br>10 days for colitis<br>17 days for microbiota
[117]	Curcumin-galactomannoside	250 mg/kg<br>21 days
[118]	Curcumin	50 or 150 mg kg<br>7 days
[119]	Curcumin in hydroxyethyl starch microspheres	6.8 mg/kg<br>7 days
[120]	Curcumin	200 mg/kg<br>7 days
[121]	Honey polyphenols	10.5mg/kg<br>7 days
[122]	*Glochidion ellipticum Wight* extracts	200 mg/kg of HexE<br>200 mg/kg DcmE<br>200 mg/kg ButE<br>7 days
[123]	Galangin	40 mg/kg<br>20 days
[124]	Galangin	15 mg/kg<br>7 days

Author	Compound	Dose/time
[125]	Taxifolin	100 mg/kg<br>14 days
[126]	*Flos lonicerae*	Hy-, Lo and Lu of L25, 50, 100 mg/kg<br>10 days
[127]	Oroxindin	12.5, 25, 50 mg/kg<br>10 days
[128]	Polyphenolic maqui extract (*Aristotelia chilensis*)	50 mg/kg/day<br>Curative Group: 4 days after TNBS induction<br>Preventive Group: 7 days before TNBS induction
[129]	Polyphenolic maqui extract (*Aristotelia chilensis*)	50 mg/kg<br>post-treatment: 4 days after TNBS induction<br>pre-treatment: 7 days before TNBS and 4 days after TNBS induction
[130]	Acacetin	50 and 150 mg/kg<br>9 days
[131]	Flavonoid composition rich *P. Subpeltata Ortega*	200 or 400 mg/kg<br>11 days
[132]	Juglone (JUG)	0.04 mg/ml juglone<br>17 days
[133]	Hydroxytyrosol (HYT) from olive leaves extract (OLE)	150 mg/kg<br>7 days
[134]	Lingonberry (LB)	10 or 100 mg/kg<br>9 days
[135]	*Caragana sinica* extract	250 and 500 mg/kg<br>10 days
[136]	*Quercus brantii* (QB) extract	200 and 400 mg/kg<br>6 days
[137]	*T. Occidentalis leaf* extract (ato)	200mg/kg<br>10 days
[138]	*Dilodendron bipinnatum Radlk*. extract	20, 100 and 500 mg/kg<br>3 doses: 48, 24 and 1h before colitis induction
[139]	*Copaifera malmei leaf* infusion extract (iecm)	25, 100 and 400 mg/kg<br>72 h, 48 h, 24 h and 2 h before induction
[140]	Kaempferol (kae)	50 mg/kg/day<br>14 days
[141]	6-Paradol from seeds of *Aframomum melegueta*	50, 100 and 200 mg/kg<br>7 days
[142]	*Maesa lanceolata* hydroethanolic extract	100, 200 and 400 mg/kg<br>8 days
[143]	*Garcinia mangostana* and *α-mangostin* extract	GM: 40, 200, or 1000 mg/kg<br>α-mangostin: 30 mg/kg<br>7 days
[144]	*Garcinia pedunculata bark* (AEGP) aqueous extract	200 and 400mg/kg<br>7 days
[145]	Troxerutin	100 and 200mg/kg<br>10 days

Author	Compound	Dose/time
[146]	*P. Lentiscus leaf* aqueous extract	25, 50 and 100 mg/kg<br>7 days
[147]	Wogonin	30 mg/kg<br>7 days
[148]	Plant polyphenols (gallic acid, proanthocyanidin, ellagic acid, and tannic acid)	100 mg/kg of each polyphenol<br>6 days
[149]	Gallic acid	10 mg/kg<br>7 days
[150]	Gallic acid	20, 40 and 60mg/kg<br>7 days
[151]	Gallic acid	50 and 100 mg/kg<br>7 days
[152]	Arum maculatum	50, 100 and 150 mg/kg<br>14 days
[153]	Coumaric acid and syringic acid	coumaric acid: 100 and 150 mg/kg<br>syringic acid: 10, 25 and 50 mg/kg<br>4 days
[154]	Syringic acid	10, 25 and 50 mg/kg<br>5 days
[155]	Syringic acid	25 mg/kg<br>7 days
[156]	Apigenin	3 mg/kg/day<br>7 days
[157]	Safranal	200 and 500 mg/kg<br>7 days
[158]	Resveratrol	100 mg/kg<br>9 days
[159]	Resveratrol in polysaccharide-zein nanoparticles from Mesona chinensis	10 mg/kg<br>14 days
[160]	Oxiresveratrol (OXY)	2.5 or 5mg/kg<br>7 days
[161]	Ligustroside	1, 2, and 4 mg/kg<br>7 days
[162]	Isoimperatorin	0.1, 1 and 10 mg/kg<br>3 days
[163]	Forsythia suspensa polyphenols	200, 400 and 600 mg/kg<br>7 days
[164]	m *Callicarpa nudiflora Hook* flavonoids	400 mg/kg<br>17 days
[165]	*Calliandra haematocephala* extracts	250, 500 and 750 mg/kg<br>14 days
[166]	Diosmin	100 and 200 mg/kg<br>7 days
[167]	*Ziziphus jujuba Mill* polyphenol extracts	200 mg/mL<br>7 days
[168]	Geraniol	50 and 100 mg/kg<br>12 days
[169]	*Terminalia catappa Linn*	25, 50 and 100 mg/kg<br>7 days
[170]	*L. Dentata* or *l. Stoechas*	L. dentata: 10 and 25 mg/kg<br>or

Author	Compound	Dose/time
		L. stoechas: 10 and 25 mg/kg<br>7 days
[171]	Demethyleneberberine—DMB<br>(component from *Cortex Phellodendri Chinensis*)	150 and 300mg/kg<br>15 days
[172]	*Raphanus sativus L seeds* aqueous extract	10, 40, 70 and 100 mg/kg<br>7 days
[173]	Myrrh	125, 250 or 500 mg/kg<br>7 days
[174]	Gegen qinlian	0.3, 1.5 or 7.5g/kg<br>7 days
[175]	Ursolic acid	20 mg/kg<br>7 days
[176]	*Lion's Mane Medicinal Mushroom* and *Hericium erinaceus* (Agaricomycetes) ethanol extract	250 and 500 mg/kg<br>7 days
[177]	Polysaccharide from cultured mycelium of *Hericium erinaceus*	1.2 and 2.5g/kg<br>11 days
[178]	Polysaccharides from the edible mushroom *Hericium erinaceus*	200, 300 and 400 mg/kg<br>7 days
[179]	*Amphipterygium adstringens* extract	200 mg/kg<br>10 days
[180]	Mangiferin	30 and 60 mg/kg<br>9 days
[181]	*Portulaca oleracea L*	100, 200 and 400 mg/kg<br>7 days
[182]	*Portulaca oleracea L*	400 and 800 mg/kg<br>8 days
[183]	Andrographolide	different doses of AL-1 twice a day (5, 15 and 45 mg/kg)
[184]	*Carpolobia lutea G. Don* (Polygalaceae)	100, 200,<br>400 and 800mg/kg<br>2 days before induction + 7 days
[185]	*Lagerstroemia speciosa*	100 and 200 mg/kg<br>7 days
[186]	*Atractylodes macrocephala* and *Taraxacum herba* extracts	AM: 100 mg/kg<br>TH: 100 mg/kg<br>14 days
[187]	*Aronia berry*	diet supplemented with 4.5% lyophilized "Viking" aronia berry poder<br>35 days
[188]	*Aronia berry* extract	10 or 100 mg/kg<br>10 days
[189]	*Aronia melanocarpa* (Michx.) Elliott.	100, 300 e 600 mg/kg<br>21 days
[190]	Decursin and decursinol	10, 20 and 40 mg/kg<br>7 days
[191]	*Perilla frutescens* extract (PE)	20 or 100 mg/kg<br>7 days before + 14 days after induction

Author	Compound	Dose/time
[192]	Glyceollins from daidzein in soybean	curative and preventive treatment: 4, 10 mg/kg of glycerolin or 100mg/kg
[193]	Catalpol	10 and 80 mg/kg<br>7 days and 14 days
[194]	D-limonene	50 or 100 mg/kg<br>7 days
[195]	Liriodendrin	100mg/kg<br>10 days
[196]	Yuzu (*Citrus junos Tanaka*)	50 g/kg<br>14 days
[197]	*Veronica polita*	200mg/kg<br>7 days
[198]	*Ziziphus spina-christi* fruit extract	100, 200 and 400 mg/kg<br>5 days
[199]	Red raspberries	6 g/kg<br>6 weeks
[200]	Plumieride	10, 30 and 100 mg/kg<br>7 days
[201]	*Ipomoea asarifolia* aqueous extract	25, 50 and 100 mg/kg<br>3 days
[202]	Osthole	50 mg/kg<br>7days
[203]	*Polygonum cuspidatum Siebold & Zucc* root Extract	0.1, 0.2 g and 0.4 g/kg<br>8 days
[204]	*Alpinia officinarum*	200 mg/kg<br>5 days
[205]	Magnolol	25, 50 and 100 mg/kg<br>3 days
[206]	Magnolol contained in a butyrate-derived polymer nanoplatform	5 mg/kg<br>2, 4, 12 and 24 hours
[207]	*Trichilia catigua* ethyl-acetate fraction	200 mg/kg<br>7 days
[208]	4-methylesculetin	5 or 25 mg/kg<br>7 days
[209]	4-methylesculetin	5 and 10 mg/kg<br>96, 72, 48, 24 and 2 h before TNBS administration
[210]	Fargesin	50 mg/kg<br>11 days
[211]	Sinomenine	400 and 100 mg/kg<br>10 days
[212]	Sinomenine	10, 20, 40 mg/kg<br>7 days
[213]	Stevioside	50 and 100mg/kg<br>12 days
[214]	Sesamin	50 and 100 mg/kg<br>9 days
[215]	*Terminalia arjuna* hydroalcoholic extract	125, 250 and 500 mg/kg<br>28 days
[216]	*Persea americana Mill.* Avocado extract	50, 100 or 200 mg/kg<br>9 days
[217]	Quercetin aglycone	QUE: 0.21%, QMQ: 0.36%

Author	Compound	Dose/time
		14 days
[218]	2-O-β-d-Glucopyranosyl-l-ascorbic acid, an ascorbic acid derivative isolated from the fruits of *Lycium Barbarum L*	300 mg/kg<br>8 days
[219]	Apocynin	400 mg/kg<br>7 days
[220]	Apocynin	100, 200 and 400 mg/kg<br>7 days.
[221]	Crocin	20 mg/kg<br>8 days before + 8 days after
[222]	Crocin	50 or 200 ppm<br>21 days
[223]	Crocina	20 mg/kg<br>14 days
[224]	Salidroside (salt)	20 and 40mg/kg<br>7 days
[225]	Freeze-dried fruit powder of *Actinidia arguta*	300 and 600 mg/kg<br>10 days
[226]	*Tagetes erecta L.* flowers hydroalcoholic extract	30, 100 and 300mg/kg<br>9 days
[227]	Daidzein	10mg/kg<br>7 days
[228]	*Ajuga chamaepitys* (L.) *Schreber subsp. Chia* (Schreber)	100 mg/kg<br>3 days
[229]	*Sorbus domestica*	100 mg/kg<br>3 days
[230]	*Bryophyllum pinnatum* (Lamarck) leaf extract	250 and 500 mg/kg<br>5 days
[231]	*Piper umbellatum L.* (Piperaceae)	30, 100 and 300 mg/kg<br>72, 48, 24 and 2 h before induction
[232]	Myristicin	200 mg/kg<br>6 hours before induction
[233]	*Bruguiera gymnorrhiza leaves*	25, 50 and 100 mg/kg<br>7 days
[234]	Arjunarishta	1.8; 0.9 and 0.45 mL/kg<br>28 days
[235]	*Antrocaryon micraster*	30, 100 and 300 mg/kg<br>3 days
[236]	Piperine	10, 20 and 40 mg/kg<br>14 days
[237]	Puerarin	10 or 50 mg/kg<br>7 days
[238]	*Rumex japonicus Houtt.*	100 mg/kg<br>14 days
[239]	*Gloeostereum incarnatum*	1.0, 2.0 and 4.0 g/kg<br>21 days
[240]	Sinapic acid	10 or 50 mg/kg<br>7 days
[241]	Nerolidol	50, 100 and 150 mg/kg<br>7 days
[242]	Nerolidol (NRD)	50 mg/kg<br>10 days

Author	Compound	Dose/time
[243]	*Jasonia glutinosa (L.)* DC. extract	5, 25 or 50 mg/kg<br>20 days
[244]	*Sanhuang shu'ai*	0.8 or 1.6 g/kg<br>7 days
[245]	*Flammuliana velutipes* polysaccharide	50, 100 and 200 mg/kg<br>14 days
[246]	of the *Syringa oblata Lindl* Iridoid glycosides	20, 40 and 80 mg/kg<br>14 days
[247]	Mucilage Garden cress	100 mg/kg<br>21 days
[248]	*Otostegia fruticosa leaves* crude extract	200 and 400 mg/kg<br>6 days
[249]	*Dracocephalum kotschyi* methanol extract	10, 25 and 50 mg/kg<br>8 days
[250]	Fruit of *Rosa odorata doce var.gigantea*	125, 250 and 500 mg kg<br>7 days
[251]	*Artemisia argyi* extract	200 mg/kg<br>10 days
[252]	*Inula viscosa* ethanolic extract	400 mg/kg<br>12 days
[253]	Cepharanthine	10 mg/kg<br>7 days
[254]	*Saposhnikovia divaricat*a	50, 100 and 200 mg/kg<br>9 days
[255]	*Trigonellafoenum-graecum L.* seeds aqueous extract	500 and 1000mg/kg<br>7 days
[256]	Quinic acid	10, 30, 60 and 100 mg/kg<br>5 days
[257]	*Echinacea purpurea* extract	3 g/kg<br>10 days
[258]	*Echinacea purpurea* polysaccharide	200 mg/kg<br>21 days
[259]	Daphnetin	16 mg/kg
[260]	Oxyberberin	12.5, 25 and 50 mg/kg<br>7 days
[261]	Curculigoside (CUR), from *Curculigo orchioides Gaertn*	10 and 20 mg/kg<br>8 days
[262]	*Scrophularia striata Boiss* aqueous and hydroalcoholic Extracts	150, 300 and 600 mg/kg<br>5 days
[263]	*Picralima nitida seeds* crude alkaloidal extract	30, 100 and 300 mg/kg<br>8 days
[264]	*Mesua assamica* (King&Prain) *kosterm. Bark* ethanolic extract	100 and 200 mg/kg<br>14 days
[265]	Higenamine	10 and 30 mg/kg<br>7 days
[266]	Carboxymethyl Poria Polysaccharides	300 mg/kg/day
[267]	1,8-cineol (eucaliptol)	100 and 200 mg/kg<br>8 days
[268]	*Polygonatum Cyrtonema Hua* Oligosaccharides	0.5, 2 and 5 mg/kg<br>5 days

Author	Compound	Dose/time
[269]	Polysaccharide from the fermented mycelium of *Inonotus obliquus*	100, 200 and 400 mg/kg<br>7 days
[270]	*Pinus eldarica* aqueous and hydroalcoholic extracts	100, 200 and 400 mg/kg<br>5 days
[271]	*Terminalia chebula* ethyl acetate extract	100 and 200 mg/kg<br>7 days
[272]	*Ecklonia cava* extract	50, 100, 200 mg/kg<br>21 days
[273]	*Salvia verbenaca* extract	10 and 25 mg/kg<br>8 days
[274]	*Commiphora leptophloeos* extract	300, 400 and 500 mg/kg<br>6 days
[275]	*Sagittaria sagittifolia L.* polysaccharides	100, 200 and 400 mg/kg<br>14 days
[276]	*Tetrastigma hemsleyanum* root extract	100 and 500 mg/kg<br>7 days
[277]	Gilaburu (*Viburnum opulus L.)* fruit extract	100 mg/kg<br>3 days
[278]	Acidic polysaccharide from *Selaginella uncinata (Desv.) Spring*	50 and 100 mg/kg<br>14 days
[279]	*Lizhong decoction*	1.82 g/kg and 5.46 g/kg<br>10 days
[280]	(-)-Syringaresinol	10, 20 and 40 mg/kg<br>10 days.
[281]	Aegeline	5, 10, 20 mg/kg<br>7 days
[282]	Fraxetin	10, 30 and 60 mg/kg<br>25 days
[283]	Honeysuckle	extract added to the feed at 0.15, 0.75 and 1.5 g/kg<br>15 days
[284]	*Passiflora edulis*	8 mg/mL in drinking water<br>5 days
[285]	Jinxiang garlic (*Allium sativum L.*)	200 or 400 mg/kg/day<br>14 days
[286]	Cinnamaldehyde and hesperetin	Cinnamaldehyde: 40 or 0 mg/kg or Hesperetin: 50 or 100mg/kg<br>12 days
[287]	*Anacardium occidentale L.*	100 mg/kg<br>4 days
[288]	Loganic acid	15 and 30 mg/kg
[289]	Four sanshools of *Zanthoxylum fruit*	2.5 mg/kg<br>7 days
[290]	*Sea buckthorn*	200 mg/kg<br>21 days
**Functional foods and nutrients**		
[291]	Oat β-glucan	500 and 1000mg/kg<br>7 days after induction
[292]	Blueberry	50 mg/kg<br>14 days

Author	Compound	Dose/time
[293]	Riboflavin	25 mg/kg<br>3 days
[294]	Goat whey	1, 2 or 4 g/kg<br>17 days
[295]	Garlic oil	25, 50 and 100mg/kg/day<br>7 days
[296]	Red bean	100 mg/kg<br>4 days
[297]	Lecithin	0.5mL/kg<br>2 days
[298]	Coenzime Q10	30 mg/kg<br>7 days
[299]	Coenzime Q10	10 or 30 and 100 mg/kg<br>8 days
[300]	Honey	0.5, 0.75 and 1g<br>21 days
[301]	Flaxseed extract	300 and 500mg/kg<br>7 days
[302]	B-glucans from Lentinus edodes	500 or 1000mg/kg<br>6 days
[303]	Selenium	0.5 ppm as sodium selenite<br>8 days
[304]	Selenocysteine and selenocystine	0.9 mg/kg<br>7 days
[305]	Selenium in biogenic nanoparticles	800ng/kg<br>5 days
[306]	Camellia oil	2 mL/kg<br>20 days
[307]	Walnut extract	10 and 20 mg/kg<br>8 days
[308]	Aqueous cinnamon extract	150mg/kg<br>10 days
[309]	Cinnamon (Cinnamomum japonicum)<br>subcritical water extract	100, 300 or 500 mg/kg<br>21 days
[310]	Tocotrienol (alpha-tocopherol)	150mg/kg<br>7 days
[311]	Omega 3	EPA: 5.7gm/Kg and<br>DHA: 3.8gm/Kg<br>30 days
[312]	Eicosapentaenoic acid (EPA)	300 and 1000 mg/kg<br>28 days
[313]	Noni juice-fortified yogurt	20 g/kg of freeze-dried yogurt diet<br>containing 7%<br>7 days
[314]	Isolated from fish skin gelatin<br>hydrolysate<br>(fsghf3)	100 and 200 mg kg<br>14 days
[315]	Mannoglucan<br>(Chinese yam.)	300 mg/kg per day<br>7 days
[316]	Alpha-tocopherylquinone	50 mg/kg/day<br>7 days

**Author**	**Compound**	**Dose/time**
[317]	B-carotene	50 mg/kg<br>7 days
[318]	Momordica charantia	100 µL<br>5 days
[319]	Virgin coconut oil	500, 750 and 1000 mg/kg<br>12 days
[320]	Pumpkin polysaccharides	50 and 100 mg/kg<br>7 days
[321]	Fermented yogurt	300 µL of yogurt fermented at 37°C or 22°C
**Probiotics**		
[322]	NTU 101; *L. rhamnosus* BCRC 16000; *L. paracasei subsp. paracasei* BCRC 14023	different doses and strains daily<br>25 days
[323]	*Lactobacillus plantarum* (CAU1054 OR CAU1055, OR CAU1064) OR *Lactobacillus salivarius* CAU1301	0.2 mL of Lactobacillus<br>21 days
[324]	MegaSporeBiotic TM (MSB) probiotic capsules and MegaMucosa TM (MM) powder	MSB 1 × 109 CFU and MM 70 mg/100 g<br>11 days
[325]	*Bifidobacterium bifidum* ATCC 29521	2×108 CFU/day<br>27 days
[326]	*Lactobacillus acidophilus* XY27	LA-XY27 (1 × 109 CFU/mL, 0.1 mL/10 g) and L . bulgaricus (1.0 × 109 CFU/mL, 0.1 mL/10 g)<br>21 days
[327]	Minas Frescal probiotic cheese containing L. lactis	0.5mL<br>7 days
[328]	Probiotic yeast<br><br>*Saccharomyces boulardii* (s. Boulardii)	105 CFU/mL or 10 7 CFU/mL<br>21 days
[329]	*Lactobacillus acidophilus* KDSL 1.0901, *Lactobacillus helveticus* KDSL 1.8701, *Lactobacillus plantarum*<br>KDSL 1.0318, and mixed lactobacilli	1 × 109 CFU mL−1<br>21 days
[330]	*Lactobacillus gasseri* 4M13	750mg kg<br>14 days
[331]	*L. pentosus* A14-6 and *L. pentosus* CMY46	L. pentosus A14-6, L. pentosus CMY46, L. pentosus A14-6 plus XOS and L. pentosus CMY46 plus GOS.<br>1 × 109 CFU/200 µL/day.<br>7 days
[332]	*Lactobacillus acidophilus* C4	1 × 109 CFU/ml<br>7 days
[333]	*Exopolysaccharide Ropy Bifidobacterium pseudocatenulatum* Bi-OTA128	2 × 109 CFU in 0.2 mL of saline<br>21 days
**OTHERS**		
[334]	Insect (cockroach) *Periplaneta americana*	200 mg/kg and 100 mg/kg<br>7 days
[335]	MicroRNAs	1×10 12 vg/mL of AAV2-miR-200a<br>7 days
[336]	Insect (cockroach) *Periplaneta americana*	80, 160 and 320 mg/kg<br>21 days

Author	Compound	Dose/time
[337]	*Aspergillus awamori*	100 mg, 50 mg and 25 mg/kg<br>7 days
[338]	Maggot extracts	1 g/kg<br>12 days
[339]	Meroterpene algae 11-hydroxy-1′-O—methylamadione	1, 10 and 20 mg/kg<br>7 days
[340]	Arthrospira (Spirulina) platensis	50, 100 and 250 mg/kg<br>5 days
[341]	hydroalcoholic extracts (HA) of cyanobacterium *Spirulina platensis*	100 or 200mg/kg<br>15 days
[342]	Chinese propolis	50, 100 and 200 mg/kg<br>7 days
[343]	*Saccharina japonica*	1, 2 and 4g/kg<br>14 days
[344]	*Aphanizomenon flos-aquae*	20, 50 or 100 mg/kg<br>14 days
[345]	Melanin from *Sepia pharaonis ink*	75, 150 and 300 mg/kg<br>9 days
[346]	Tuna bioactive peptides (TBP)	200, 500 mg/kg<br>7 days
[347]	Turtle peptide	500 mg/kg<br>7 days
[348]	Oxylipin-containing lyophilized biomass from a microalga	50, 100 and 200 mg/kg<br>23 days
[349]	Fermented Mekabu aqueous solution by *Lactobacillus plantarum Sanriku-SU7*	25% AES diet of mekabu and fermented mekabu<br>3 days before + 7 days after

Legend: CAT= catalase; COX2= cyclooxigenase type 2; DNA= deoxyribonucleic acid; ERON= reactive oxygen and nitrogen specie; GPx= glutathione peroxidase; GR= glutathione reductase; GSH= glutathione; GST= glutathione S-transferase; iNOS= inducible nitric oxide synthase; Iκ-Bα= nuclear factor of kappa light polypeptide gene enhancer in B-cells inhibitor, alpha; LP= lipid peroxidation; LPO= lipoxygenase; MDA= malondialdehyde; MPO= myeloperoxidase; NF-Kb= Nuclear Factor Kappa-light-chain-enhancer of activated B Cells; NO= nitric oxide; NOX= nicotinamide adenine dinucleotide phosphate-oxidase; Nrf2= nuclear factor erythroid 2; PTN= protein; RONS= reactive oxygen and nitrogen species; SOD= superoxide dismutase; TAC= total antioxidant capacity

References

26. Zhu, D.; Ma, Y.; Ding, S.; Jiang, H.; Fang, J. Effects of Melatonin on Intestinal Microbiota and Oxidative Stress in Colitis Mice. BioMed Res. Int. 2018, 2018, 2607679.

27. Cao, J.; Lu, M.; Yan, W.; Li, L.; Ma, H. Dehydroepiandrosterone alleviates intestinal inflammatory damage via GPR30-mediated Nrf2 activation and NLRP3 inflammasome inhibition in colitis mice. Free Radic. Biol. Med. 2021, 172, 386–402.

28. Elhassan, Y.H. Anti-inflammatory, anti-apoptotic, and antioxidant effects of obestatin on the colonic mucosa following acetic acid-induced colitis. Folia Morphol. 2023, 82, 641–655.

29. Zhao, Y.; Sun, Y.; Ding, Y.; Wang, X.; Zhou, Y.; Li, W.; Huang, S; Li, Z; Kong, L; Guo, Q; et al. GL-V9, a new synthetic flavonoid derivative, ameliorates DSS-induced colitis against oxidative stress by up-regulating Trx-1 expression via activation of AMPK/FOXO3a pathway. Oncotarget 2015, 6, 26291–26307.

30. Hong, C.-O.; Rhee, C.H.; Pyo, M.C.; Lee, K.-W. Anti-inflammatory effect of glucose-lysine Maillard reaction products on intestinal inflammation model in vivo. Int. Immunopharmacol. 2017, 52, 324–332.

31. Li, J.; Chen, H.; Wang, B.; Cai, C.; Yang, X.; Chai, Z.; Feng, W. ZnO nanoparticles act as supportive therapy in DSS-induced ulcerative colitis in mice by maintaining gut homeostasis and activating Nrf2 signaling. Sci. Rep. 2017, 7, 43126.

32. Nagarjun, S.; Dhadde, S.B.; Veerapur, V.P.; Thippeswamy, B.; Chandakavathe, B.N. Ameliorative effect of chromium-d-phenylalanine complex on indomethacin-induced inflammatory bowel disease in rats. Biomed. Pharmacother. 2017, 89, 1061–1066.

33. Shen, N.-Y.; Bi, J.-B.; Zhang, J.-Y.; Zhang, S.-M.; Gu, J.-X.; Qu, K.; Liu, C. Hydrogen-rich water protects against inflammatory bowel disease in mice by inhibiting endoplasmic reticulum stress and promoting heme oxygenase-1 expression. World J. Gastroenterol. 2017, 23, 1375–1386.

34. Zarzecki, M.S.; Bortolotto, V.C.; Poetini, M.R.; Araujo, S.M.; de Paula, M.T.; Roman, S.S.; Spiazzi, C; Cibin, F.W.; Rodrigues, O..E; Jesse, C.R.; et al. Anti-Inflammatory and Anti-Oxidant Effects of p-Chloro-phenyl-selenoesterol on TNBS-Induced Inflammatory Bowel Disease in Mice. J. Cell. Biochem. 2017, 118, 709–717.

35. Zhu, C.; Zhang, S.; Song, C.; Zhang, Y.; Ling, Q.; Hoffmann, P.R.; Li, J; Chen, T.; Zheng, W.; Huang, Z. Selenium nanoparticles decorated with Ulva lactuca polysaccharide potentially attenuate colitis by inhibiting NF-κB mediated hyper inflammation. J. Nanobiotechnol. 2017, 15, 20.

36. Ji, Y.; Dai, Z.; Sun, S.; Ma, X.; Yang, Y.; Tso, P.; Wu, G.; Wu, Z. Hydroxyproline Attenuates Dextran Sulfate Sodium-Induced Colitis in Mice: Involvment of the NF-κB Signaling and Oxidative Stress. Mol. Nutr. Food Res. 2018, 62, e1800494.

37. Gao, Y.; Bai, D.; Zhao, Y.; Zhu, Q.; Zhou, Y.; Li, Z.; Lu, N. LL202 ameliorates colitis against oxidative stress of macrophage by activation of the Nrf2/HO-1 pathway. J. Cell. Physiol. 2019, 234, 10625–10639.

38. Chen, Y.; Zheng, Z.; Li, C.; Pan, Y.; Tang, X.; Wang, X.J. Synthetic Imine Resveratrol Analog 2-Methoxyl-3,6-Dihydroxyl-IRA Ameliorates Colitis by Activating Protective Nrf2 Pathway and Inhibiting NLRP3 Expression. Oxidative Med. Cell. Longev. 2019, 2019, 7180284.

39. Mei, Y.; Wang, Z.; Zhang, Y.; Wan, T.; Xue, J.; He, W.; Luo, Y.; Xu, Y.; Bai, X,.; Wang, Q. FA-97, a New Synthetic Caffeic Acid Phenethyl Ester Derivative, Ameliorates DSS-Induced Colitis Against Oxidative Stress by Activating Nrf2/HO-1 Pathway. Front. Immunol. 2019;10, 2969.

40. Zheng, J.D.; He, Y.; Yu, H.Y.; Liu, Y.L.; Ge, Y.X.; Li, X.T.; et al. Unconjugated bilirubin alleviates experimental ulcerative colitis by regulating intestinal barrier function and immune inflammation. World J. Gastroenterol. 2019, 25, 1865–1878.

41. Ahmed, O.; Abdel-Halim, M.; Farid, A.; Elamir, A. Taurine loaded chitosan-pectin nanoparticle shows curative effect against acetic acid-induced colitis in rats. Chem. -Biol. Interact. 2022, 351, 109715.

42. Liu, C.; Yan, X.; Zhang, Y.; Yang, M.; Ma, Y.; Zhang, Y.; Xu, Q.; Tu, K.; Zhang, M. Oral administration of turmeric-derived exosome-like nanovesicles with anti-inflammatory and pro-resolving bioactions for murine colitis therapy. J. Nanobiotechnol. 2022, 20,206.

43. Saber, S.; Alamri, M.M.S.; Alfaifi, J.; Saleh, L.A.; Abdel-Ghany, S.; Aboregela, A.M.; Farrag, A.A.; Almaeen, A.H.; Adam, M.I.E.; AlQahtani, A.A.J.; et al. (R,R)-BD-AcAc2 Mitigates Chronic Colitis in Rats: A Promising Multi-Pronged Approach Modulating Inflammasome Activity, Autophagy, and Pyroptosis. Pharmaceuticals 2023, 16, 28.

44. Török, S.; Almási, N.; Veszelka, M.; Börzsei, D.; Szabó, R; Varga, C. Protective Effects of H2S Donor Treatment in Experimental Colitis: A Focus on Antioxidants. Antioxidants. 2023, 12, 1025.

45. Ye, R.; Guo, Q.; Huang, J.; Wang, Z.; Chen, Y.; Dong, Y. Eucommia ulmoides polysaccharide modified nano-selenium effectively alleviated DSS-induced colitis through enhancing intestinal mucosal barrier function and antioxidant capacity. J. Nanobiotechnol. 2023, 21, 222.

46. Zeeshan, M.; Ain, Q.U.; Weigmann, B.; Story, D.; Smith, B.R.; Ali, H. Dual pH and microbial-sensitive galactosylated polymeric nanocargoes for multi-level targeting to combat ulcerative colitis. Asian J. Pharm. Sci. 2023, 18, 100831.

47. Zhu, M.-Z.; Xu, H.-M.; Liang, Y.-J.; Xu, J.; Yue, N.-N.; Zhang, Y.; Tian, C.M.;, Yao, J.; Wang, L.S.; Nie, Y.Q.; et al. Edible exosome-like nanoparticles from portulaca oleracea L mitigate DSS-induced colitis via facilitating double-positive CD4+CD8+T cells expansion. J. Nanobiotechnol. 2023, 21, 19.

48. Sadar, S.S.; Vyawahare, N.S.; Bodhankar, S.L. Ferulic acid ameliorates TNBS-induced ulcerative colitis through modulation of cytokines, oxidative stress, iNOs, COX-2, and apoptosis in laboratory rats. EXCLI J. 2016, 15, 482–499.

49. Ghasemi-Dehnoo, M.; Amini-Khoei, H.; Lorigooini, Z.; AnjomShoa, M.; Rafieian-Kopaei, M. Ferulic acid ameliorates ulcerative colitis in a rat model via the inhibition of two LPS-TLR4-NF-κB and NF-κB-INOS-NO signaling pathways and thus alleviating the inflammatory, oxidative and apoptotic conditions in the colon tissue. Inflammopharmacology 2023, 31, 2587–2597.

50. Chen, T.; Chen, L.; Luo, F.; Xu, Y.; Wu, D.; Li, Y.; Zhao, R.; Hua, Z.; Hu, J. Efficient oral delivery of resveratrol-loaded cyclodextrin-metal organic framework for alleviation of ulcerative colitis. Int. J. Pharm. 2023, 646, 123496.

51. Moura, F.A.; de Andrade, K.Q.; de Araújo, O.R.P.; Nunes-Souza, V.; Santos, J.C.d.F.; Rabelo, L.A.; Goulart, M.O. Colonic and Hepatic Modulation by Lipoic Acid and/or N-Acetylcysteine Supplementation in Mild Ulcerative Colitis Induced by Dextran Sodium Sulfate in Rats. Oxidative Med. Cell. Longev. 2016, 2016, 4047362.

52. Zhu, C.; Ling, Q.; Cai, Z.; Wang, Y.; Zhang, Y.; Hoffmann, P.R.; Zheng, W.; Zhou, T.; Huang, Z. Selenium-Containing Phycocyanin from Se-Enriched Spirulina platensis Reduces Inflammation in Dextran Sulfate Sodium-Induced Colitis by Inhibiting NF-κB Activation. J. Agric. Food Chem. 2016, 64, 5060–5070.

53. Li, Y.; Pan, X.; Yin, M.; Li, C.; Han, L. Preventive Effect of Lycopene in Dextran Sulfate Sodium-Induced Ulcerative Colitis Mice through the Regulation of TLR4/TRIF/NF-κB Signaling Pathway and Tight Junctions. J. Agric. Food Chem. 2021, 69, 13500–13509.

54. Ahmedy, O.A.; Ibrahim, S.M.; Salem, H.H.; Kandil, E.A. Antiulcerogenic effect of melittin via mitigating TLR4/TRAF6 mediated NF-κB and p38MAPK pathways in acetic acid-induced ulcerative colitis in mice. Chem. -Biol. Interact. 2020, 331, 109276.

55. Xiang, X.; Jiang, Q.; Shao, W.; Li, J.; Zhou, Y.; Chen, L.; Deng, S.; Zheng, B.; Chen, Y.et al. Protective Effects of Shrimp Peptide on Dextran Sulfate Sodium-Induced Colitis in Mice. Front. Nutr. 2021, 8, 773064.

56. Zuo, K.; Tang, K.; Liang, Y.; Xu, Y.; Sheng, K.; Kong, X.; Wang, J.; Zhu, F.; Zha, X.; Wang, Y. Purification and antioxidant and anti-Inflammatory activity of extracellular polysaccharopeptide from sanghuang mushroom, Sanghuangporus lonicericola. J. Sci. Food Agric. 2021, 101, 1009–1020.

57. Guo, W.; Tang, X.; Zhang, Q.; Zhao, J.; Mao, B.; Zhang, H.; Cui, S. Mitigation of Dextran-Sodium-Sulfate-Induced Colitis in Mice through Oral Administration of Microbiome-Derived Inosine and Its Underlying Mechanisms. Int. J. Mol. Sci. 2023, 24, 13852.

58. Silveira, A.K.; Gomes, H.M.; Fröhlich, N.T.; Possa, L.; Santos, L.; Kessler, F.; Martins, A.; Rodrigues, M.S.; De Oliveira, J.; do Nascimento, N.D.; et al. Sodium Butyrate Protects Against Intestinal Oxidative

Damage and Neuroinflammation in the Prefrontal Cortex of Ulcerative Colitis Mice Model. Immunol. Investig. 2023, 52, 796–814.

59. Ullah, H.; Deng, T.; Ali, M.; Farooqui, N.A.; Alsholi, D.M.; Siddiqui, N.Z.; Rehman, A.U.; Ali, S,.;Ilyas, M.; Wang, L.; et al. Sea Conch Peptides Hydrolysate Alleviates DSS-Induced Colitis in Mice through Immune Modulation and Gut Microbiota Restoration. Molecules 2023, 28, 21.

60. Zhang, W.; Zhang, X.; Lv, X.; Qu, A.; Liang, W.; Wang, L.; Zhao, P.; Wu, Z. Oral Delivery of Astaxanthin via Carboxymethyl Chitosan-Modified Nanoparticles for Ulcerative Colitis Treatment. Molecules 2024, 29, 1291.

61. Sakai, S.; Nishida, A.; Ohno, M.; Inatomi, O.; Bamba, S.; Sugimoto, M.; Kawahara, M.; Andoh, A. Astaxanthin, a xanthophyll carotenoid, prevents development of dextran sulphate sodium-induced murine colitis. J. Clin. Biochem. Nutr. 2019, 64, 66–72.

62. Adjadj, M.; Boumerfeg, S.; Noureddine, C.; Baghiani, A.; Khennouf, S.; Arrar, L.; Mubarak, M.S. Protective Effect of Paronychia argentea L. on Acetic Acid Induced Ulcerative Colitis in Mice by Regulating Antioxidant Parameters and inflammatory Markers. Wulfenia J. 2015, 22, 148–172.

63. Costa, C.; Tanimoto, A.; Quaglio, A.; Almeida, L.; Severi, J.; Di Stasi, L.C. Anti-inflammatory effects of Brazilian ginseng (Pfaffia paniculata) on TNBS-induced intestinal inflammation: Experimental evidence. Int. Immunopharmacol. 2015, 28, 459–469.

64. Chaparala, A.; Tashkandi, H.; Chumanevich, A.A.; Witalison, E.E.; Windust, A.; Cui, T.; Nagarkatti, M; Nagarkatti, P; Hofseth, L.J. Molecules from American Ginseng Suppress Colitis through Nuclear Factor Erythroid-2-Related Factor 2. Nutrients 2020, 12, 1850.

65. Chaparala, A.; Poudyal, D.; Tashkandi, H.; Witalison, E.E.; Chumanevich, A.A.; Hofseth, J.L.; Nguyen, I.; Hardy, O; Pittman, D.L.; Wyatt, M.D.; et al. Panaxynol, a bioactive component of American ginseng, targets macrophages and suppresses colitis in mice. Oncotarget 2020, 11, 2026–2036.

66. de Melo, M.N.; Soares, L.A.L.; Porto, C.R.d.C.; de Araújo, A.A.; Almeida, M.d.G.; de Souza, T.P.; Petrovick , P.R; de Araújo, R.F. Jr; Guerra, G.C. Spray-dried extract of Phyllanthus niruri L. reduces mucosal damage in rats with intestinal inflammation. J. Pharm. Pharmacol. 2015, 67, 1107–1118.

67. Bastaki, S.M.A.; Al Ahmed, M.M.; Al Zaabi, A.; Amir, N.; Adeghate, E. Effect of turmeric on colon histology, body weight, ulcer, IL-23, MPO and glutathione in acetic-acid-induced inflammatory bowel disease in rats. BMC Complement. Altern. Med. 2016, 16, 72.

68. Boussenna, A.; Joubert-Zakeyh, J.; Fraisse, D.; Pereira, B.; Vasson, M.-P.; Texier, O.; Felgines, C. Dietary Supplementation with a Low Dose of Polyphenol-Rich Grape Pomace Extract Prevents Dextran Sulfate Sodium-Induced Colitis in Rats. J. Med. Food 2016, 19, 755–758.

69. Chu, L.; Zhang, S.; Wu, W.; Gong, Y.; Chen, Z.; Wen, Y.; Wang, Y.; Wang, L. Grape seed proanthocyanidin extract alleviates inflammation in experimental colitis mice by inhibiting NF-κB signaling pathway. Environ. Toxicol. 2024, 39, 2572–2582.

70. Sheng, K.; Zhang, G.; Sun, M.; He, S.; Kong, X.; Wang, J.; Zhu, F.; Zha, X; Wang, Y. Grape seed proanthocyanidin extract ameliorates dextran sulfate sodium-induced colitis through intestinal barrier improvement, oxidative stress reduction, and inflammatory cytokines and gut microbiota modulation. Food Funct. 2020, 11, 7817–7829.

71. Choi, Y.H.; Bae, J.-K.; Chae, H.-S.; Nhoek, P.; Choi, J.-S.; Nhoek, P; Choi, J.S; Chin, Y.W. Isoliquiritigenin ameliorates dextran sulfate sodium-induced colitis through the inhibition of MAPK pathway. Int. Immunopharmacol. 2016, 31, 223–232.

72. Cibiček, N.; Roubalová, L.; Vrba, J.; Zatloukalová, M.; Ehrmann, J.; Zapletalová, J.; Večeřa, R.; Křen, V.; Ulrichová, J. Protective effect of isoquercitrin against acute dextran sulfate sodium-induced rat colitis depends on the severity of tissue damage. Pharmacol. Rep. 2016, 68, 1197–1204.

73. Dönder, Y.; Arikan, T.B.; Baykan, M.; Akyüz, M.; Öz, A.B. Effects of quercitrin on bacterial translocation in a rat model of experimental colitis. Asian, J. Surg. 2018, 41, 543–550.

74. Denis, M.-C.; Roy, D.; Yeganeh, P.R.; Desjardins, Y.; Varin, T.; Haddad, N.; Amre, D.; Sané, A.T.; Garofalo, C.; Furtos, A.; et al. Apple peel polyphenols: A key player in the prevention and treatment of experimental inflammatory bowel disease Clin. Sci. 2016, 130, 2217–2237.

75. Pastrelo, M.M.; Ribeiro, C.C.D.; Duarte, J.W.; Gollucke, A.P.B.; Artigiani-Neto, R.; Ribeiro, D.A.; Miszputen, S.J.; Fujiyama Oshima, C.T.; Ribeiro Paiotti, A.P. Effect of concentrated apple extract on experimental colitis induced by acetic acid. Int. J. Mol. Cell. Med. 2017, 6, 38–49.

76. Li, Y.; Shen, L.; Luo, H. Luteolin ameliorates dextran sulfate sodium-induced colitis in mice possibly through activation of the Nrf2 signaling pathway. Int. Immunopharmacol. 2016, 40, 24–31.

77. Li, B.; Guo, Y.; Jia, X.; Cai, Y.; Zhang, Y.; Yang, Q. Luteolin alleviates ulcerative colitis in rats via regulating immune response, oxidative stress, and metabolic profiling. Open Med. 2023, 18, 20230785.

78. Sahu, B.D.; Kumar, J.M.; Sistla, R. Fisetin, a dietary flavonoid, ameliorates experimental colitis in mice: Relevance of NF-kappa B signaling. J. Nutr. Biochem. 2016, 28, 171–182.

79. Khosropour, P.; Sajjadi, S.E.; Talebi, A.; Minaiyan, M. Anti-inflammatory effect of Myrtus communis hydroalcoholic extract and essential oil on acetic acid-induced colitis in rats. J. Rep. Pharm. Sci. 2019, 8, 204–210.

80. Sen, A.; Yuksel, M.; Bulut, G.; Bitis, L.; Ercan, F.; Ozyilmaz-Yay, N.; Ozben, A.; Hamit, C.; Sevil, O.; Goksel, S. Therapeutic Potential of Myrtus communis Subsp.communis Extract Against Acetic ACID-Induced Colonic Inflammation in Rats. J. Food Biochem. 2017, 41, e12297.

81. Zhang, H.; Deng, A.; Zhang, Z.; Yu, Z.; Liu, Y.; Peng, S.; Wu, L.; Qin, H.; Wang, W. The protective effect of epicatechin on experimental ulcerative colitis in mice is mediated by increasing antioxidation and by the inhibition of NF-κB pathway. Pharmacol. Rep. 2016, 68, 514–520.

82. Anzoise, M.L.; Basso, A.R.; Del Mauro, J.S.; Carranza, A.; Ordieres, G.L.; Gorzalczany, S. Potential usefulness of methyl gallate in the treatment of experimental colitis. Inflammopharmacology 2018, 26, 839–849.

83. de Morais Lima, G.R.; Machado, F.D.; Périco, L.L.; de Faria, F.M.; Luiz-Ferreira, A.; Souza Brito, A.R.; Pellizzon, C.H.; Hiruma-Lima, C.A.; Tavares, J.F.; Barbosa Filho, J.M.; Batista, L.M. Anti-inflammatory intestinal activity of Combretum duarteanum Cambess. in trinitrobenzene sulfonic acid colitis model. World J. Gastroenterol. 2017, 23, 1353–1366.

84. de Santana Souza, M.T.; Teixeira, D.F.; de Oliveira, J.P.; Oliveira, A.S.; Quintans-Júnior, L.J.; Correa, C.B.; Camargo, E.A. Protective effect of carvacrol on acetic acid-induced colitis. Biomed. Pharmacother. 2017, 96, 313–319.

85. Dey, Y.N.; Sharma, G.; Wanjari, M.M.; Kumar, D.; Lomash, V.; Jadhav, A.D. Beneficial effect of Amorphophallus paeoniifolius tuber on experimental ulcerative colitis in rats. Pharm. Biol. 2017, 55, 53–62.

86. Jin, B.R.; Chung, K.S.; Cheon, S.Y.; Lee, M.; Hwang, S.; Noh Hwang, S.; Rhee, K.J; An, H.J. Rosmarinic acid suppresses colonic inflammation in dextran sulphate sodium (DSS)-induced mice via dual inhibition of NF-κB and STAT3 activation. Sci. Rep. 2017, 7, 46252.

87. Formiga, R.d.O.; Júnior, E.B.A.; Vasconcelos, R.C.; Guerra, G.C.B.; de Araújo, A.A.; de Carvalho, T.G.; Garcia, V.B.; de Araújo Junior, R.F.; Gadelha, F.A.A.F.; Vieira, G.C.; Sobral, M.V.; et al. p-Cymene and

Rosmarinic Acid Ameliorate TNBS-Induced Intestinal Inflammation Upkeeping ZO-1 and MUC-2: Role of Antioxidant System and Immunomodulation. Int. J. Mol. Sci. 2020, 21, 5870.

88. Marinho, S.; Illanes, M.; Ávila-Román, J.; Motilva, V.; Talero, E. Anti-Inflammatory Effects of Rosmarinic Acid-Loaded Nanovesicles in Acute Colitis through Modulation of NLRP3 Inflammasome. Biomolecules 2021, 11, 162.

89. Kim, Y.; Wu, A.G.; Jaja-Chimedza, A.; Graf, B.L.; Waterman, C.; Verzi, M.P.; Raskin, I. Isothiocyanate-enriched moringa seed extract alleviates ulcerative colitis symptoms in mice. PLoS ONE 2017, 12, e0184709.

90. Murugan, R.; Saravanan, S.; Parimelazhagan, T. Study of intestinal anti-inflammatory activity of Phoenix loureiroi Kunth (Arecaceae) fruit. Biomed. Pharmacother. 2017, 93, 156–164.

91. Patel, P.P.; Trivedi, N.D. Effect of karanjin on 2,4,6-trinitrobenzenesulfonic acid-induced colitis in Balb/c mice. Indian, J. Pharmacol. 2017, 49, 161–167.

92. Suluvoy, J.K.; Sakthivel, K.M.; Guruvayoorappan, C.; Grace, V.B. Protective effect of Averrhoa bilimbi L. fruit extract on ulcerative colitis in wistar rats via regulation of inflammatory mediators and cytokines. Biomed. Pharmacother. 2017, 91, 1113–1121.

93. Vezza, T.; Algieri, F.; Rodríguez-Nogales, A.; Garrido-Mesa, J.; Utrilla, M.P.; Talhaoui, N.; Gómez-Caravaca, A.M., Segura-Carretero, A.; Rodríguez-Cabezas, M.E.; Monteleone, G.; Gálvez, J. Immunomodulatory properties of Olea europaea leaf extract in intestinal inflammation. Mol. Nutr. Food Res. 2017, 61, 1601066.

94. Vochyánová, Z.; Pokorná, M.; Rotrekl, D.; Smékal, V.; Fictum, P.; Suchý, P.; Gajdziok, J.; Šmejkal, K.; Hošek, J. Prenylated flavonoid morusin protects against TNBS-induced colitis in rats. PLoS ONE 2017, 12, e0182464.

95. Wang, B.; Wu, C. Dietary soy isoflavones alleviate dextran sulfate sodium-induced inflammation and oxidative stress in mice. Exp. Ther. Med. 2017, 14, 276–282.

96. Xu, B.; Li, Y.L.; Xu, M.; Yu, C.C.; Lian, M.Q.; Tang, Z.Y.; Li, C.X.; Lin, Y. Geniposide ameliorates TNBS-induced experimental colitis in rats via reducing inflammatory cytokine release and restoring impaired intestinal barrier function. Acta Pharmacol. Sinica 2017, 38, 688–698.

97. Zhang, Z.; Li, Y.; Shen, P.; Li, S.; Lu, X.; Liu, J.; Cao, Y; Liu, B; Fu, Y.; Zhang, N. Administration of geniposide ameliorates dextran sulfate sodium-induced colitis in mice via inhibition of inflammation and mucosal damage. Int. Immunopharmacol. 2017, 49, 168–177.

98. Zhuge, X.; Jin, X.; Ji, T.; Li, R.; Xue, L.; Yu, W.; Quan, Z.; Tong, H.; Xu, F. Geniposide ameliorates dextran sulfate sodium-induced ulcerative colitis via KEAP1-Nrf2 signaling pathway. J. Ethnopharmacol. 2023, 314, 116626.

99. Yang, L.; Shen, L.; Li, Y.; Li, Y.; Yu, S.; Wang, S. Hyperoside attenuates dextran sulfate sodium-induced colitis in mice possibly via activation of the Nrf2 signalling pathway. J. Inflamm. 2017, 14, 25.

100. Alabi, Q.K.; Akomolafe, R.O.; Omole, J.G.; Adefisayo, M.A.; Ogundipe, O.L.; Aturamu, A.; Sanya, J.O. Polyphenol-rich extract of Ocimum gratissimum leaves ameliorates colitis via attenuating colonic mucosa injury and regulating pro-inflammatory cytokines production and oxidative stress. Biomed. Pharmacother. 2018, 103, 812–822.

101. Abiodun, O.O.; Nwadike, N.; Ogunleye, F.N.; Sosanya, A.S. Ocimum gratissimum Linn. (Lamiaceae) protects wistar rats against inflammation and oxidative stress in trinitrobenzene sulfonic acid-induced colitis. Thai J. Pharm. Sci. 2020, 44, 136–144.

102. Gupta, R.A.; Motiwala, M.N.; Mahajan, U.N.; Sabre, S.G. Protective effect of Sesbania grandiflora on acetic acid induced ulcerative colitis in mice by inhibition of TNF-α and IL-6J. Ethnopharmacol. 2018, 219, 222–232.

103. Kim Kim, K.J.; Park, J.M.; Lee, J.S.; Kim, Y.S.; Kangwan, N.; Han, Y.M.; Kang EA, An JM, Park YK, Hahm, K.B. Oligonol prevented the relapse of dextran sulfate sodium-ulcerative colitis through enhancing NRF2-mediated antioxidative defense mechanism. J. Physiol. Pharmacol. 2018, 69, 359–371.

104. Chamanara, M.; Abdollahi, A.; Rezayat, S.M.; Ghazi-Khansari, M.; Dehpour, A.; Nassireslami, E.; Rashidian, A. Thymol reduces acetic acid-induced inflammatory response through inhibition of NF-kB signaling pathway in rat colon tissue. Inflammopharmacology 2019, 27, 1275–1283.

105. Tahmasebi, P.; Froushani, S.M.A.; Ahangaran, N.A. Thymol has beneficial effects on the experimental model of ulcerative colitis. Avicenna J. Phytomed. 2019, 9, 538–550.

106. da Silva-Maia, J.K.; Batista, G.; Cazarin, C.B.B.; Soares, E.S.; Junior, S.B.; Leal, R.F.; et al. Aqueous Extract of Brazilian Berry (Myrciaria jaboticaba) Peel Improves Inflammatory Parameters and Modulates Lactobacillus and Bifidobacterium in Rats with Induced-Colitis. Nutrients 2019, 11, 2776.

107. Du, Y.; Ding, H.; Vanarsa, K.; Soomro, S.; Baig, S.; Hicks, J.; Mohan. C. Low dose Epigallocatechin Gallate Alleviates Experimental Colitis by Subduing Inflammatory Cells and Cytokines, and Improving Intestinal Permeability. Nutrients 2019, 11, 1743.

108. Bitzer, Z.T.; Elias, R.J.; Vijay-Kumar, M.; Lambert, J.D. (-)-Epigallocatechin-3-gallate decreases colonic inflammation and permeability in a mouse model of colitis, but reduces macronutrient digestion and exacerbates weight loss. Mol. Nutr. Food Res. 2016, 60, 2267–2274.

109. Bing, X.; Xuelei, L.; Wanwei, D.; Linlang, L.; Keyan, C. EGCG Maintains Th1/Th2 Balance and Mitigates Ulcerative Colitis Induced by Dextran Sulfate Sodium through TLR4/MyD88/NF-κB Signaling Pathway in Rats. Can. J. Gastroenterol. Hepatol. 2017, 2017, 3057268.

110. Firoozi, D.; Nekooeian, A.A.; Tanideh, N.; Mazloom, Z.; Mokhtari, M.; Sartang, M.M. The Healing Effects of Hydroalcoholic Extract of Carum Copticum, L. on Experimental Colitis in Rats. Iran. J. Med. Sci. 2019, 44, 501–510.

111. Guo, K.; Ren, J.; Gu, G.; Wang, G.; Gong, W.; Wu, X.W.; Ren, H.; Hong, Z; Li, J. Hesperidin Protects Against Intestinal Inflammation by Restoring Intestinal Barrier Function and Up-Regulating Treg Cells. Mol. Nutr. Food Res. 2019, 63, e1800975.

112. Guazelli, C.F.; Fattori, V.; Ferraz, C.R.; Borghi, S.M.; Casagrande, R.; Baracat, M.M.; Verri, W.A. J. Antioxidant and anti-inflammatory effects of hesperidin methyl chalcone in experimental ulcerative colitis. Chem. Interactions 2021, 333, 109315.

113. Shafik, N.M.; Gaber, R.A.; Mohamed, D.A.; Ebeid, A.M. Hesperidin modulates dextran sulfate sodium-induced ulcerative colitis in rats: Targeting sphingosine kinase-1-sphingosine 1 phosphate signaling pathway, mitochondrial biogenesis, inflammation, and apoptosis. J. Biochem. Mol. Toxicol. 2019, 33, e22312.

114. Stan, M.S.; Voicu, S.N.; Caruntu, S.; Nica, I.C.; Olah, N.-K.; Burtescu, R.; Balta, C.; Rosu, M.; Herman, H.; Hermenean, A.; Antioxidant and Anti-Inflammatory Properties of a Thuja occidentalis Mother Tincture for the Treatment of Ulcerative Colitis. Antioxidants 2019, 8, 416.

115. Zhang, Z.; Li, S.; Cao, H.; Shen, P.; Liu, J.; Fu, Y.; Cao, Y.; Zhang, N. The protective role of phloretin against dextran sulfate sodium-induced ulcerative colitis in mice. Food Funct. 2018, 10, 422–431.

116. Wu, M.; Li, P.; An, Y.; Ren, J.; Yan, D.; Cui, J.; Li, D.; Li, M.; Wang, M.; Zhang, G. Phloretin ameliorates dextran sulfate sodium-induced ulcerative colitis in mice by regulating the gut microbiota. Pharmacol. Res. 2019, 150, 104489.

117. Sheethal, S.; Ratheesh, M.; Jose, S.P.; Asha, S.; Krishnakumar, I.M.; Sandya, S.; Girishkumar, B.; Grace, J. Anti-Ulcerative Effect of Curcumin-Galactomannoside Complex on Acetic Acid-Induced Experimental Model by Inhibiting Inflammation and Oxidative Stress. Inflammation 2020, 43, 1411–1422.

118. Guo, X.; Xu, Y.; Geng, R.; Qiu, J.; He, X. Curcumin Alleviates Dextran Sulfate Sodium-Induced Colitis in Mice Through Regulating Gut Microbiota. Mol. Nutr. Food Res. 2022, 66, 2100943.

119. Huang, D.; Wang, Y.; Xu, C.; Zou, M.; Ming, Y.; Luo, F.; Xu, Z.; Miao, Y.; Wang, N.; Lin, Z.; Weng, Z. Colon-targeted hydroxyethyl starch-curcumin microspheres with high loading capacity ameliorate ulcerative colitis via alleviating oxidative stress, regulating inflammation, and modulating gut microbiota. Int. J. Biol. Macromol. 2024, 266, 131107.

120. Erarslan, A.S.; Ozmerdivenli, R.; Sirinyıldız, F.; Cevik, O.; Gumus, E.; Cesur, G. Therapeutic and prophylactic role of vitamin D and curcumin in acetic acid-induced acute ulcerative colitis model. Toxicol. Mech. Methods 2023, 33, 480–489.

121. Zhao, H.A.; Cheng, N.; Zhou, W.Q.; Chen, S.N.; Wang, Q.; Gao, H.; Xue, X.; Wu, L.; Cao, W. Honey Polyphenols Ameliorate DSS-Induced Ulcerative Colitis via Modulating Gut Microbiota in Rats. Mol. Nutr. Food Res. 2019, 63, 1900638.

122. Hossen, I.; Hua, W.; Mehmood, A.; Raka, R.N.; Jingyi, S.; Jian-Ming, J.; Min, X.; Shakoor, A.; Yanping, C.; Wang, C.; Glochidion ellipticum Wight extracts ameliorate dextran sulfate sodium-induced colitis in mice by modulating nuclear factor kappa-light-chain-enhancer of activated B cells signalling pathway. J. Pharm. Pharmacol. 2021, 73, 410–423.

123. Gerges, S.H.; Tolba, M.F.; Elsherbiny, D.A.; El-Demerdash, E. The natural flavonoid galangin ameliorates dextran sulphate sodium-induced ulcerative colitis in mice: Effect on Toll-like receptor 4, inflammation and oxidative stress. Basic Clin. Pharmacol. 2020, 127, 10–20.

124. Xuan, H.; Ou, A.; Hao, S.; Shi, J.; Jin, X.L. Galangin Protects against Symptoms of Dextran Sodium Sulfate-Induced Acute Colitis by Activating Autophagy and Modulating the Gut Microbiota. Nutrients 2020, 12, 347.

125. Hou, J.; Hu, M.; Zhang, L.; Gao, Y.; Ma, L.; Xu, Q. Dietary Taxifolin Protects Against Dextran Sulfate Sodium-Induced Colitis via NF-κB Signaling, Enhancing Intestinal Barrier and Modulating Gut Microbiota. Front. Immunol. 2020, 11, 631809.

126. Liu, D.; Yu, X.; Sun, H.; Zhang, W.; Liu, G.; Zhu, L. Flos lonicerae flavonoids attenuate experimental ulcerative colitis in rats via suppression of NF-κB signaling pathway. Naunyn-Schmiedeberg's Arch. Pharmacol. 2020, 393, 2481–2494.

127. Liu, Q.; Zuo, R.; Wang, K.; Nong, F.F.; Fu, Y.J.; Huang, S.W.; Pan, Z.F.; Zhang, X.L.; Deng, X.L; Zhang, X.X.; et al. Oroxindin inhibits macrophage NLRP3 inflammasome activation in DSS-induced ulcerative colitis in mice via suppressing TXNIP-dependent NF-κB pathway. Acta Pharmacol. Sinica 2020, 41, 771–781.

128. Ortiz, T.; Argüelles-Arias, F.; Illanes, M.; García-Montes, J.-M.; Talero, E.; Macías-García, L.; Alcudia, A.; Vázquez-Román, V.; Motilva, V.; De-Miguel, M. Polyphenolic Maqui Extract as a Potential Nutraceutical to Treat TNBS-Induced Crohn's Disease by the Regulation of Antioxidant and Anti-Inflammatory Pathways. Nutrients 2020, 12, 1752.

129. Ortiz-Cerda, T.; Argüelles-Arias, F.; Macías-García, L.; Vázquez-Román, V.; Tapia, G.; Xie, K.; García-García, M.D.; Merinero, M.; García-Montes, J.M.; Alcudia, A.; Effects of polyphenolic maqui (Aristotelia chilensis) extract on the inhibition of NLRP3 inflammasome and activation of mast cells in a mouse model of Crohn’s disease-like colitis. Front. Immunol. 2024, 14, 1229767.

130. Ren, J.; Yue, B.; Wang, H.; Zhang, B.; Luo, X.; Yu, Z.; Zhang, J.; Ren, Y.; Mani, S.; Wang, Z.; et al. Acacetin Ameliorates Experimental Colitis in Mice via Inhibiting Macrophage Inflammatory Response and Regulating the Composition of Gut Microbiota. Front. Physiol. 2021, 11, 577237.

131. Shanmugam, S.; Thangaraj, P.; Lima, B.d.S.; Trindade, G.G.; Narain, N.; Mara de Oliveira, E.S.A.; Santin, J.R.; Broering, M.F.; Serafini, M.R.; Quintans-Júnior, L.J.; et al. Protective effects of flavonoid composition rich P. subpeltata Ortega. on indomethacin induced experimental ulcerative colitis in rat models of inflammatory bowel diseases. J. Ethnopharmacol. 2020, 248, 112350.

132. Chen, S.; Wu, X.; Yu, Z. Juglone Suppresses Inflammation and Oxidative Stress in Colitis Mice. Front. Immunol. 2021, 12, 674341.

133. Elmaksoud, H.A.A.; Motawea, M.H.; Desoky, A.A.; Elharrif, M.G.; Ibrahimi, A. Hydroxytyrosol alleviate intestinal inflammation, oxidative stress and apoptosis resulted in ulcerative colitis. Biomed. Pharmacother. 2021, 142, 112073.

134. Jeon, Y.-D.; Lee, J.-H.; Kang, S.-H.; Myung, H.; Jin, J.-S. Lingonberry Fruit Ethanol Extract Ameliorates DSS-Induced Ulcerative Colitis In Vivo and In Vitro. Appl. Sci. 2021, 11, 7955.

135. Li, T.; Zou, Q.P.; Huang, F.; Cheng, G.G.; Mao, Z.W.; Wang, T.; Dong, F.W.; Li, B.J.; He, H.P.; Li, Y.P. Flower extract of Caragana sinica. ameliorates DSS-induced ulcerative colitis by affecting TLR4/NF-KB and TLR4/MAPK signaling pathway in a mouse model. Iran. J. Basic Med. Sci. 2021, 24, 595–603.

136. Naini, M.A.; Mehrvarzi, S.; Zargari-Samadnejadi, A.; Tanideh, N.; Ghorbani, M.; Dehghanian, A.; Hasanzarrini, M.; Banaee, F.; Koohi-Hosseinabadi, O.; Irajie, C.; et al. The Antioxidant and Anti-Inflammatory Effects of Quercus brantii Extract on TNBS-Induced Ulcerative Colitis in Rats. Evid. -Based Complement. Altern. Med. 2021, 2021, 3075973.

137. Oladele, J.O.; Anyim, J.C.; Oyeleke, O.M.; Olowookere, B.D.; Bamigboye, M.O.; Oladele, O.T.; Oladiji, A.T. Telfairia occidentalis mitigates dextran sodium sulfate-induced ulcerative colitis in rats via suppression of oxidative stress, lipid peroxidation, and inflammation. J. Food Biochem. 2021, 45, e13873.

138. de Oliveira, R.G.; Damazo, A.S.; Antonielli, L.F.; Miyajima, F.; Pavan, E.; Duckworth, C.A.; Lima, J.C.D.S.; Arunachalam, K.; Martins, D.T.O. Dilodendron bipinnatum Radlk. extract alleviates ulcerative colitis induced by TNBS in rats by reducing inflammatory cell infiltration, TNF-α and IL-1β concentrations, IL-17 and COX-2 expressions, supporting mucus production and promotes an antioxidant effect. J. Ethnopharmacol. 2021, 269, 113735.

139. Pavan, E.; Damazo, A.S.; Arunachalam, K.; Almeida, P.O.d.A.; Oliveira, D.M.; Venturini, C.L.; Figueiredo, F.F.; Cruz, T.C.D.D.; Silva, J.V.D.; Martins, D.T.O. Copaifera malmei Harms leaves infusion attenuates TNBS-ulcerative colitis through modulation of cytokines, oxidative stress and mucus in experimental rats. J. Ethnopharmacol. 2021, 267, 113499.

140. Qu, Y.F.; Li, X.Y.; Xu, F.Y.; Zhao, S.M.; Wu, X.M.; Wang, Y.Z.; Xie, J. Kaempferol Alleviates Murine Experimental Colitis by Restoring Gut Microbiota and Inhibiting the LPS-TLR4-NF-kappa B Axis. Front. Immunol. 2021, 12, 679897.

141. Rafeeq, M.; Murad, H.A.S.; Abdallah, H.M.; El-Halawany, A.M. Protective effect of 6-paradol in acetic acid-induced ulcerative colitis in rats. BMC Complement. Med. Ther. 2021, 21,28.

142. Tagne, M.A.F.; Tchoffo, A.; Noubissi, P.A.; Mazo, A.G.; Kom, B.; Mukam, J.N.; Sokeng Dongmo, S.; Kamgang, R. Effects of hydro-ethanolic extract of leaves of Maesa lanceolata (Mursinaceae) on acetic acid-induced ulcerative colitis in rats. Inflammopharmacology 2021, 29, 1211–1223.

143. Tatiya-Aphiradee, N.; Chatuphonprasert, W.; Jarukamjorn, K. Ethanolic Garcinia mangostana extract and α-mangostin improve dextran sulfate sodium-induced ulcerative colitis via the suppression of inflammatory and oxidative responses in ICR mice. J. Ethnopharmacol. 2021, 265, 113384.

144. Mundugaru, R.; Udaykumar, P.; Kumar, K.N.S.; Nayak, S.; Jacob, T.; Alfarhan, A.H.; Rajakrishnan, R. Protective effect of garcinia pedunculata fruit rind in acetic acid induced ulcerative colitis. Farmacia 2019, 67, 160–166.

145. Wang, X.; Gao, Y.; Wang, L.; Yang, D.; Bu, W.; Gou, L.; Huang, J.; Duan, X.; Pan, Y.; Cao, S.; Troxerutin Improves Dextran Sulfate Sodium-Induced Ulcerative Colitis in Mice. J. Agric. Food Chem. 2021, 69, 2729–2744.

146. Zahouani, Y.; Ben Rhouma, K.; Kacem, K.; Sebai, H.; Sakly, M. Aqueous Leaf Extract of Pistacia lentiscus Improves Acute Acetic Acid-Induced Colitis in Rats by Reducing Inflammation and Oxidative Stress. J. Med. Food 2021, 24, 697–708.

147. Zhou, Y.A.-O.; Dou, F.; Song, H.; Liu, T. Anti-ulcerative effects of wogonin on ulcerative colitis induced by dextran sulfate sodium via Nrf2/TLR4/NF-κB signaling pathway in BALB/c mice. Environ. Toxicol. 2022, 37, 954–963.

148. Chen, H.; Li, Y.; Wang, J.; Zheng, T.; Wu, C.; Cui, M.; Feng, Y.; Ye, H.; Dong, Z.;, Dang, Y. Plant Polyphenols Attenuate DSS-induced Ulcerative Colitis in Mice via Antioxidation, Anti-inflammation and Microbiota Regulation. Int. J. Mol. Sci. 2023, 24, 10828.

149. Pandurangan, A.K.; Mohebali, N.; Norhaizan, M.E.; Looi, C.Y. Gallic acid attenuates dextran sulfate sodium-induced experimental colitis in BALB/c mice. Drug Des. Dev. Ther. 2015, 9, 3923–3934.

150. Zhu, L.; Gu, P.; Shen, H. Gallic acid improved inflammation via NF-κB pathway in TNBS-induced ulcerative colitis. Int. Immunopharmacol. 2019, 67, 129–137.

151. Bayramoglu, G.; Senturk, H.; Kanbak, G.; Canbek, M.; Bayramoglu, A.; Dokumacioglu, E.; Engür, S. Gallic Acid Reduces Experimental Colitis in Rats by Downregulation of Cathepsin and Oxidative Stress. Erciyes Med. J. 2020, 42, 213–217.

152. Doğan, G.T.; Kepekçi, R.A.; Bostancıeri, N.; Tarakçıoğlu, M. Protective effect of Arum maculatum against dextran sulfate sodium induced colitis in rats. Biotech. Histochem. 2023, 98, 456–465.

153. Ekhtiar, M.; Ghasemi-Dehnoo, M.; Mirzaei, Y.; Azadegan-Dehkordi, F.; Amini-Khoei, H.; Lorigooini, Z.; Samiei-Sefat, A.; Bagheri, N. The coumaric acid and syringic acid ameliorate acetic acid-induced ulcerative colitis in rats via modulator of Nrf2/HO-1 and pro-inflammatory cytokines. Int. Immunopharmacol. 2023, 120, 110309.

154. Ghasemi-Dehnoo, M.; Amini-Khoei, H.; Lorigooini, Z.; AnjomShoa, M.; Bijad, E.; Rafieian-Kopaei, M. Inhibition of TLR4, NF-κB, and INOS pathways mediates ameliorative effect of syringic acid in experimental ulcerative colitis in rats. Inflammopharmacology 2023, 32, 795–808.

155. Fang, W.; Zhu, S.; Niu, Z.; Yin, Y. The protective effect of syringic acid on dextran sulfate sodium-induced experimental colitis in BALB/c mice. Drug Dev. Res. 2019, 80, 731–740.

156. Shibrya, E.E.; Rashed, R.R.; El Fattah, M.A.A.; El-Ghazaly, M.A.; Kenawy, S.A. Apigenin and Exposure to Low Dose Gamma Radiation Ameliorate Acetic Acid-Induced Ulcerative Colitis in Rats. Dose-Response 2023, 21, 15593258231155787.

157. Lertnimitphun, P.; Jiang, Y.; Kim, N.; Fu, W.; Zheng, C.; Tan, H.; Zhou, H.; Zhang, X.; Pei, W.; Lu, Y.; Safranal Alleviates Dextran Sulfate Sodium-Induced Colitis and Suppresses Macrophage-Mediated Inflammation. Front. Pharmacol. 2019, 10, 1281.

158. Xu, X.; Ocansey, D.K.W.; Pei, B.; Zhang, Y.; Wang, N.; Wang, Z.; Mao F. Resveratrol alleviates DSS-induced IBD in mice by regulating the intestinal microbiota-macrophage-arginine metabolism axis. Eur. J. Med Res. 2023, 28, 319.

159. Yang, J.; Lin, J.; Zhang, W.; Shen, M.; Wang, Y.; Xie, J. Resveratrol-loaded pH-responsive Mesona chinensis polysaccharides-zein nanoparticles for effective treatment of ulcerative colitis. J. Sci. Food Agric. 2024, 104, 3992–4003.

160. Yeom, J.; Ma, S.; Kim, J.-K.; Lim, Y.-H. Oxyresveratrol Ameliorates Dextran Sulfate Sodium-Induced Colitis in Rats by Suppressing Inflammation. Molecules 2021, 26, 2630.

161. Gao, R.; Ren, Y.; Xue, P.; Sheng, Y.; Yang, Q.; Dai, Y.; Zhang, X.; Lin, Z.; Liu, T.; Geng, Y.; Protective Effect of the Polyphenol Ligustroside on Colitis Induced with Dextran Sulfate Sodium in Mice. Nutrients 2024, 16, 522.

162. Goodarzi, S.; Abdolghaffari, A.H.; Najafi, B.; Hamedani, M.P.; Tavakoli, S.; Marvi, M.; Isoimperatorin alleviates acetic acid-induced colitis in rats. Asian Pac. J. Trop. Biomed. 2024, 14, 147–153.

163. Lv, W.; Jin, W.; Lin, J.; Wang, Z.; Ma, Y.; Zhang, W.; Zhu, Y.; Hu, Y.; Qu, Q.; Guo, S. Forsythia suspensa polyphenols regulate macrophage M1 polarization to alleviate intestinal inflammation in mice. Phytomedicine 2024, 125, 155336.

164. Nong, K.; Qin, X.; Liu, Z.; Wang, Z.; Wu, Y.; Zhang, B..; Chen, W.; Fang, X.; Liu, Y.; Wang, X.; et al. Potential effects and mechanism of flavonoids extract of Callicarpa nudiflora Hook on DSS-induced colitis in mice. Phytomedicine 2024, 128, 155523.

165. Rehman, I.U.; Saleem, M.; Raza, S.A.; Bashir, S.; Muhammad, T.; Asghar, S.; Qamar, M.U.; Shah, T.A.; Bin Jardan, Y.A.; Mekonnen, A.B.; et al. Anti-ulcerative colitis effects of chemically characterized extracts from Calliandra haematocephala in acetic acid-induced ulcerative colitis. Front. Chem. 2024, 12, 1291230.

166. Salem, M.B.; El-Lakkany, N.M.; El-Din, S.H.S.; Hammam, O.A.; Samir, S. Diosmin alleviates ulcerative colitis in mice by increasing Akkermansia muciniphila abundance, improving intestinal barrier function, and modulating the NF-κB and Nrf2 pathways. Heliyon 2024, 10, e27527.

167. Wei, X.; Ma, N.; Yang, W.; Tian, J.; Liu, H.; Fang, H. Polyphenol Extracts from Ziziphus jujuba Mill. "Junzao" Attenuates Ulcerative Colitis by Inhibiting the NLRP3 and MAPKs Signaling Pathways and Regulating Gut Microbiota Homeostasis in Mice. Mol. Nutr. Food Res. 2024, 68, e2300643.

168. Medicherla, K.; Sahu, B.D.; Kuncha, M.; Kumar, J.M.; Sudhakar, G.; Sistla, R. Oral administration of geraniol ameliorates acute experimental murine colitis by inhibiting pro-inflammatory cytokines and NF-κB signaling. Food Funct. 2015, 6, 2984–2995.

169. Abiodun, O.O.; Rodríguez-Nogales, A.; Algieri, F.; Gomez-Caravaca, A.M.; Segura-Carretero, A.; Utrilla, M.P.; Rodriguez-Cabezas, M.E.; Galvez, J. Antiinflammatory and immunomodulatory activity of an ethanolic extract from the stem bark of Terminalia catappa L. (Combretaceae): In vitro and in vivo evidences. J. Ethnopharmacol. 2016, 192, 309–319.

170. Algieri, F.; Rodriguez-Nogales, A.; Vezza, T.; Garrido-Mesa, J.; Garrido-Mesa, N.; Utrilla, M.P.; González-Tejero, M.R.; Casares-Porcel, M.; Molero-Mesa, J.; Del Mar Contreras, M.; et al. Anti-inflammatory activity of hydroalcoholic extracts of Lavandula dentata L. and Lavandula stoechas L. J. Ethnopharmacol. 2016, 190, 142–158.

171. Chen, Y.Y.; Li, R.Y.; Shi, M.J.; Zhao, Y.X.; Yan, Y.; Xu, X.X.; Zhang, M.; Zhao, X.T; Zhang, Y.B. Demethyleneberberine alleviates inflammatory bowel disease in mice through regulating NF-κB signaling and T-helper cell homeostasis. Inflamm. Res. 2017, 66, 187–196.

172. Choi, K.-C.; Cho, S.-W.; Kook, S.-H.; Chun, S.-R.; Bhattarai, G.; Poudel, S.B.; Kim, M.K.; Lee, K.Y.; Lee, J.C. Intestinal anti-inflammatory activity of the seeds of Raphanus sativus L. in experimental ulcerative colitis models. J. Ethnopharmacol. 2016, 179, 55–65.

173. Fatani, A.J.; Alrojayee, F.S.; Parmar, M.Y.; Abuohashish, H.M.; Ahmed, M.M.; Al-Rejaie, S.S. Myrrh attenuates oxidative and inflammatory processes in acetic acid-induced ulcerative colitis. Exp. Ther. Med. 2016, 12, 730–738.

174. Li, R.; Chen, Y.; Shi, M.; Xu, X.; Zhao, Y.; Wu, X.; Zhang Y. Gegen Qinlian decoction alleviates experimental colitis via suppressing TLR4/NF-κB signaling and enhancing antioxidant effect. Phytomedicine 2016, 23, 1012–1020.

175. Liu, B.; Piao, X.; Guo, L.; Liu, S.; Chai, F.; Gao, L. Ursolic acid protects against ulcerative colitis via anti-inflammatory and antioxidant effects in mice. Mol. Med. Rep. 2016, 13, 4779–4785.

176. Qin, M.; Geng, Y.; Lu, Z.; Xu, H.; Shi, J.S.; Xu, X.; Xu, Z.H. Anti-Inflammatory Effects of Ethanol Extract of Lion's Mane Medicinal Mushroom, Hericium erinaceus (Agaricomycetes), in Mice with Ulcerative Colitis. Int. J. Med. Mushrooms 2016, 18, 227–234.

177. Wang, D.; Zhang, Y.; Yang, S.; Zhao, D.; Wang, M. A polysaccharide from cultured mycelium of Hericium erinaceus relieves ulcerative colitis by counteracting oxidative stress and improving mitochondrial function. Int. J. Biol. Macromol. 2019, 125, 572–579.

178. Li, H.; Feng, J.; Liu, C.; Hou, S.; Meng, J.; Liu, J.-Y.; Zilong, S.; Chang, M.C. Polysaccharides from an edible mushroom, Hericium erinaceus, alleviate ulcerative colitis in mice by inhibiting the NLRP3 inflammasomes and reestablish intestinal homeostasis. Int. J. Biol. Macromol. 2024, 267, 131251.

179. Rodriguez-Canales, M.; Jimenez-Rivas, R.; Canales-Martinez, M.M.; Garcia-Lopez, A.J.; Rivera-Yañez, N.; Nieto-Yañez, O.; Ledesma-Soto, Y; Sanchez-Torres, L.E; Rodriguez-Sosa, M.; Terrazas, L.I.;. Protective Effect of Amphipterygium adstringens Extract on Dextran Sulphate Sodium-Induced Ulcerative Colitis in Mice. Mediat. Inflamm. 2016, 2016, 8543561.

180. Somani, S.; Zambad, S.; Modi, K. Mangiferin attenuates DSS colitis in mice: Molecular docking and in┬ávivo approach. Chemico-Biol. Interact. 2016, 253, 18–26.

181. Yang, X.; Yan, Y.; Li, J.; Tang, Z.; Sun, J.; Zhang, H.; Hao, S.; Wen, A.; Liu, L. Protective effects of ethanol extract from Portulaca oleracea L on dextran sulphate sodium-induced mice ulcerative colitis involving anti-inflammatory and antioxidant. Am. J. Transl. Res. 2016, 8, 2138–2148.

182. Ning, K.; Shi, C.; Chi, Y.-Y.; Zhou, Y.-F.; Zheng, W.; Duan, Y.; Tong, W.; Xie, Q.; Xiang, H. Portulaca oleracea L. polysaccharide alleviates dextran sulfate sodium-induced ulcerative colitis by regulating intestinal homeostasis. Int. J. Biol. Macromol. 2024, 256, 128375.

183. Yang, Y.; Yan, H.; Jing, M.; Zhang, Z.; Zhang, G.; Sun, Y.; Shan, L.; Yu, P.; Wang, Y.; Xu, L. Andrographolide derivative AL-1 ameliorates TNBS-induced colitis in mice: Involvement of NF-κB and PPAR-γsignaling pathways. Sci. Rep. 2016, 6, 29716.

184. Abiodun, O.O.; Oshinloye, A.O. Carpolobia lutea G. Don (Polygalaceae) Inhibits Inflammation and Oxidative Stress in an Acetic Acid Induced Model of Rat Colitis. Drug Res. 2017, 67, 20–24.

185. Chaudhary, G.; Mahajan, U.B.; Goyal, S.N.; Ojha, S.; Patil, C.R.; Subramanya, S.B. Protective effect of Lagerstroemia speciosa against dextran sulfate sodium induced ulcerative colitis in C57BL/6 mice. Am. J. Transl. Res. 2017, 9, 1792–1800.

186. Han, K.H.; Park, J.M.; Jeong, M.; Han, Y.M.; Go, E.J.; Park, J.; Kim, H.; Han, J.G.; Kwon, O.; Hahm, K.B. Heme Oxygenase-1 Induction and Anti-inflammatory Actions of Atractylodes macrocephala and Taraxacum herba Extracts Prevented Colitis and Was More Effective than Sulfasalazine in Preventing Relapse. Gut Liver 2017, 11, 655–666.

187. Pei, R.; Liu, J.; Martin, D.A.; Valdez, J.C.; Jeffety, J.; Barrett-Wilt, G.A.; Liu, Z.; Bolling, B.W. Aronia Berry Supplementation Mitigates Inflammation in T Cell Transfer-Induced Colitis by Decreasing Oxidative Stress. Nutrients 2019, 11, 1316,.

188. Kang, S.-H.; Jeon, Y.-D.; Moon, K.-H.; Lee, J.-H.; Kim, D.-G.; Kim, W.; Myung, H.; Kim, J.S.; Kim, H.J;, Bang, K.S.; Aronia Berry Extract Ameliorates the Severity of Dextran Sodium Sulfate-Induced Ulcerative Colitis in Mice. J. Med. Food 2017, 20, 667–67.

189. Li, Y.; Tsopmejio, I.S.N.; Diao, Z.; Xiao, H.; Wang, X.; Jin, Z.; Song, H. Aronia melanocarpa (Michx.) Elliott. attenuates dextran sulfate sodium-induced Inflammatory Bowel Disease via regulation of inflammation-related signaling pathways and modulation of the gut microbiota. J. Ethnopharmacol. 2022, 292, 115190.

190. Oh, S.R.; Ok, S.; Jung, T.S.; Jeon, S.O.; Park, J.M.; Jung, J.W.; Ryu, D.S. Protective effect of decursin and decursinol angelate-rich Angelica gigas Nakai extract on dextran sulfate sodium-induced murine ulcerative colitis. Asian Pac. J. Trop. Med. 2017, 10, 864–870.

191. Park, D.D.; Yum, H.W.; Zhong, X.; Kim, S.H.; Kim, S.H.; Kim, D.H.; Kim, D.H.; Kim, S.J.; Na, H.K.; Sato, A.; et al. Perilla frutescens Extracts Protects against Dextran Sulfate Sodium-Induced Murine Colitis: NF-κB, STAT3, and Nrf2 as Putative Targets. Front. Pharmacol. 2017, 8, 482.

192. Seo, H.; Oh, J.; Hahn, D.; Kwon, C.-S.; Lee, J.S.; Kim, J.-S. Protective Effect of Glyceollins in a Mouse Model of Dextran Sulfate Sodium-Induced Colitis. J. Med. Food 2017, 20, 1055–1062.

193. Xiong, Y.; Shi, L.; Wang, L.; Zhou, Z.; Wang, C.; Lin, Y.; Luo, D.; Qiu, J.; Chen, D. Activation of sirtuin 1 by catalpol-induced down-regulation of microRNA-132 attenuates endoplasmic reticulum stress in colitis. Pharmacol. Res. 2017, 123, 73–82.

194. Yu, L.; Yan, J.; Sun, Z. D-limonene exhibits anti-inflammatory and antioxidant properties in an ulcerative colitis rat model via regulation of iNOS, COX-2, PGE2 and ERK signaling pathways. Mol. Med. Rep. 2017, 15, 2339–2346.

195. Zhang, Z.; Yang, L.; Wang, B.; Zhang, L.; Zhang, Q.; Li, D.; Zhang, S.; Gao, H.; Wang, X. Protective role of liriodendrin in mice with dextran sulphate sodium-induced ulcerative colitis. Int. Immunopharmacol. 2017, 52, 203–210.

196. Abe, H.; Ishioka, M.; Fujita, Y.; Umeno, A.; Yasunaga, M.; Sato, A.; Ohnishi, S.; Suzuki, S.; Ishida, N.; Shichiri, M.; et al. Yuzu (Citrus junos Tanaka) Peel Attenuates Dextran Sulfate Sodium-induced Murine Experimental Colitis. J. Oleo Sci. 2018, 67, 335–344.

197. Akanda, M.R.; Nam, H.H.; Tian, W.; Islam, A,.; Choo, B.K.; Park, B.Y. Regulation of JAK2/STAT3 and NF-κB signal transduction pathways; Veronica polita alleviates dextran sulfate sodium-induced murine colitis. Biomed. Pharmacother. 2018, 100, 296–303.

198. Almeer, R.S.; Mahmoud, S.M.; Amin, H.K.; Moneim, A.E.A. Ziziphus spina-christi fruit extract suppresses oxidative stress and p38 MAPK expression in ulcerative colitis in rats via induction of Nrf2 and HO-1 expression. Food Chem. Toxicol. 2018, 115, 49–62, .

199. Bibi, S.; Kang, Y.; Du, M.; Zhu, M.-J. Dietary red raspberries attenuate dextran sulfate sodium-induced acute colitis. J. Nutr. Biochem. 2017, 51, 40–46.

200. Boeing, T.; de Souza, P.; Bonomini, T.J.; Mariano, L.N.B.; Somensi, L.B.; Lucinda, R.M.; Malheiros, A.; da Silva, L.M.; Andrade, S.F. Antioxidant and anti-inflammatory effect of plumieride in dextran sulfate sodium-induced colitis in mice. Biomed. Pharmacother. 2018, 99, 697–703.

201. da Silva, V.C.; de Araújo, A.A.; de Souza Araújo, D.F.; Souza Lima, M.C.J.; Vasconcelos, R.C.; de Araújo Júnior, R.F.; Langasnner, S.M.Z.; de Freitas Fernandes Pedrosa, M.; de Medeiros, C.A.C.X.; Guerra, G.C.B. Intestinal Anti-Inflammatory Activity of the Aqueous Extract from Ipomoea asarifolia in DNBS-Induced Colitis in Rats. Int. J. Mol. Sci. 2018, 19, 4016.

202. Khairy, H.; Saleh, H.; Badr, A.M.; Marie, M.-A.S. Therapeutic efficacy of osthole against dinitrobenzene sulphonic acid induced-colitis in rats. Biomed. Pharmacother. 2018, 100, 42–51.

203. Liu, B.; Li, S.; Sui, X.; Guo, L.; Liu, X.; Li, H.; Gao, L.; Cai, S.; Li, Y.; Wang, T.; et al. Root Extract of Polygonum cuspidatum Siebold & Zucc. Ameliorates DSS-Induced Ulcerative Colitis by Affecting NF-kappaB Signaling Pathway in a Mouse Model via Synergistic Effects of Polydatin, Resveratrol, and Emodin. Front. Pharmacol. 2018, 9, 347.

204. Rajendiran, V.; Natarajan, V.; Devaraj, S.N. Anti-inflammatory activity of Alpinia officinarum hance on rat colon inflammation and tissue damage in DSS induced acute and chronic colitis models. Food Sci. Hum. Wellness 2018, 7, 273–281.

205. Shen, P.; Zhang, Z.; He, Y.; Gu, C.; Zhu, K.; Li, S.; Li, Y.; Lu, X., Liu, J.; Zhang, N.; et al. Magnolol treatment attenuates dextran sulphate sodium-induced murine experimental colitis by regulating inflammation and mucosal damage. Life Sci. 2018, 196, 69–76.

206. Fan, X.; Zhang, Z.; Gao, W.; Pan, Q.; Luo, K.; He, B.; Pu, Y. An Engineered Butyrate-Derived Polymer Nanoplatform as a Mucosa-Healing Enhancer Potentiates the Therapeutic Effect of Magnolol in Inflammatory Bowel Disease. ACS Nano 2024, 18, 229–244.

207. Vicentini, F.A.; Barbosa, M.M.C.; Fortunato, M.C.; Amado, C.A.B.; Comar, J.F.; Longhini, R.; de Mello, J.C.P.; Natali, M.R.M. Treatment with Trichilia catigua ethyl-acetate fraction improves healing and reduces oxidative stress in TNBS-induced colitis in rats. Biomed. Pharmacother. 2018, 107, 194–202.

208. Witaicenis, A.; de Oliveira, E.C.S.; Tanimoto, A.; Zorzella-Pezavento, S.F.G.; de Oliveira, S.L.; Sartori, A.; Di Stasi, L.C. 4-methylesculetin, a coumarin derivative, ameliorates dextran sulfate sodium-induced intestinal inflammation. Chem. -Biol. Interact. 2018, 280, 59–63.

209. Tanimoto, A.; Witaicenis, A.; Caruso, P.; Piva, H.M.; Araujo, G.C.; Moraes, F.R.; Fossey, M.C.; Cornélio, M.L.; Souza, F.P.; Di Stasi, L.C. 4-Methylesculetin, a natural coumarin with intestinal anti-inflammatory activity, elicits a glutathione antioxidant response by different mechanisms. Chem. -Biol. Interact. 2020, 315, 108876.

210. Yue, B.; Ren, Y.-J.; Zhang, J.-J.; Luo, X.-P.; Yu, Z.-L.; Ren, G.-Y.; Sun, A.N.; Deng, C.; Wang, Z.T.; Dou, W. Anti-Inflammatory Effects of Fargesin on Chemically Induced Inflammatory Bowel Disease in Mice. Molecules 2018, 23, 1380.

211. Zhou, Y.; Liu, H.; Song, J.; Cao, L.; Tang, L.; Qi, C. Sinomenine alleviates dextran sulfate sodium-induced colitis via the Nrf2/NQO-1 signaling pathway. Mol. Med. Rep. 2018, 18, 3691–3698.

212. Niu, Z.; Li, X.; Yang, X.; Sun, Z. Protective effects of sinomenine against dextran sulfate sodium-induced ulcerative colitis in rats via alteration of HO-1/Nrf2 and inflammatory pathway. Inflammopharmacology 2024, 32, 2007–2022.

213. Alavala, S.; Sangaraju, R.; Nalban, N.; Sahu, B.D.; Jerald, M.K.; Kilari, E.K.; Sistla, R. Stevioside, a diterpenoid glycoside, shows anti-inflammatory property against Dextran Sulphate Sodium-induced ulcerative colitis in mice. Eur. J. Pharmacol. 2019, 855, 192–201.

214. Bai, X.; Gou, X.; Cai, P.; Xu, C.; Cao, L.; Zhao, Z.; Huang, M.; Jin, J. Sesamin Enhances Nrf2-Mediated Protective Defense against Oxidative Stress and Inflammation in Colitis via AKT and ERK Activation. Oxidative Med. Cell. Longev. 2019, 2019, 2432416.

215. Cota, D.; Mishra, S.; Shengule, S. Beneficial role of Terminalia arjuna hydro-alcoholic extract in colitis and its possible mechanism. J. Ethnopharmacol. 2019, 230, 117–125.

216. Hong, J.Y.; Chung, K.S.; Shin, J.S.; Park, G.; Jang, Y.P.; Lee, K.T. Anti-Colitic Effects of Ethanol Extract of Persea americana Mill. through Suppression of Pro-Inflammatory Mediators via NF-κB/STAT3 Inactivation in Dextran Sulfate Sodium-Induced Colitis Mice. Int. J. Mol. Sci. 2019, 20, 177.

217. Hong, Z.; Piao, M. Effect of Quercetin Monoglycosides on Oxidative Stress and Gut Microbiota Diversity in Mice with Dextran Sodium Sulphate-Induced Colitis. BioMed Res. Int. 2018, 2018, 8343052.

218. Huang, K.; Dong, W.; Liu, W.; Yan, Y.; Wan, P.; Peng, Y.; Xu, Y.; Zeng, X.; Cao, Y. 2-O -β-d-Glucopyranosyl-l-ascorbic Acid, an Ascorbic Acid Derivative Isolated from the Fruits of Lycium Barbarum L., Modulates Gut Microbiota and Palliates Colitis in Dextran Sodium Sulfate-Induced Colitis in Mice. J. Agric. Food Chem. 2019, 67, 11408–11419.

219. Hwang, Y.-J.; Nam, S.-J.; Chun, W.; Kim, S.I.; Park, S.C.; Kang, C.D.; Lee, S.J Anti-inflammatory effects of apocynin on dextran sulfate sodium-induced mouse colitis model. PLoS ONE 2019, 14, e0217642.

220. Kouki, A.; Ferjani, W.; Dang, P.M.-C.; Ghanem-Boughanmi, N.; Souli, A.; Ben-Attia, M.; El-Benna, J. Preventive Anti-inflammatory Effects of Apocynin on Acetic Acid-Induced Colitis in Rats. Inflammation 2024, 47, 438–453.

221. Khodir, A.E.; Said, E.; Atif, H.; ElKashef, H.A.; Salem,. HA. Targeting Nrf2/HO-1 signaling by crocin: Role in attenuation of AA-induced ulcerative colitis in rats. Biomed. Pharmacother. 2019, 110, 389–399.

222. Rezaei, N.; Avan, A.; Pashirzad, M.; Rahmani, F.; Moradi Marjaneh, R.; Behnam-Rassouli, R.; Shafiee, M.; Ryzhikov, M.; Hashemzehi, M.; Ariakia, F.; et al. Crocin as a novel therapeutic agent against colitis. Drug Chem. Toxicol. 2020, 43, 514–521.

223. Albalawi, G.A.; Albalawi, M.Z.; Alsubaie, K.T.; Albalawi, A.Z.; Elewa, M.A.; Hashem, K.S.; et Al-Gayyar, M.M.H. Curative effects of crocin in ulcerative colitis via modulating apoptosis and inflammation. Int. Immunopharmacol. 2023, 118, 110138.

224. Li, H.; Shen, L.; Lv, T.; Wang, R.; Zhang, N.; Peng, H.; Diao, W. Salidroside attenuates dextran sulfate sodium-induced colitis in mice via SIRT1/FoxOs signaling pathway. Eur. J. Pharmacol. 2019, 861, 172591.

225. Lian, L.; Zhang, S.; Yu, Z.; Ge, H.; Qi, S.; Zhang, X.; Long, L.; Xiong, X.; Chu, D.; Ma, X.; The dietary freeze-dried fruit powder of Actinidia arguta ameliorates dextran sulphate sodium-induced ulcerative colitis in mice by inhibiting the activation of MAPKs. Food Funct. 2019, 10, 5768–5778.

226. Meurer, M.C.; Mees, M.; Mariano, L.N.B.; Boeing, T.; Somensi, L.B.; Mariott, M.; da Silva, R.C.M.V.A.F.; Dos Santos, A.C.; Longo, B.; Santos França, T.C.; et al. Hydroalcoholic extract of Tagetes erecta L. flowers, rich in the carotenoid lutein, attenuates inflammatory cytokine secretion and improves the oxidative stress in an animal model of ulcerative colitis. Nutr. Res. 2019, 66, 95–106.

227. Shen, J.; Li, N.; Zhang, X. Daidzein Ameliorates Dextran Sulfate Sodium-Induced Experimental Colitis in Mice by Regulating NF-κB Signaling. J. Environ. Pathol. Toxicol. Oncol. 2019, 38, 29–39.

228. Akkol, E.K.; Ilhan, M.; Karpuz, B.; Taştan, H.; Sobarzo-Sánchez, E.; Khan, H. Beneficial effects of Ajuga chamaepitys (L.) Schreber subsp. chia (Schreber) and its iridoids on the colitis model: Histopathological and biochemical evidence. Food Chem. Toxicol. 2020, 144, 111589.

229. Akkol, E.K.; Dereli, F.T.G.; Taştan, H.; Sobarzo-Sánchez, E.; Khan, H. Effect of Sorbus domestica and its active constituents in an experimental model of colitis rats induced by acetic acid. J. Ethnopharmacol. 2019, 251, 112521.

230. Andrade, A.W.L.; Guerra, G.C.B.; Araújo, D.F.d.S.; Júnior, R.F.d.A.; de Araújo, A.A.; de Carvalho, T.G.; Fernandes, J.M.; Diez-Echave, P.; Hidalgo-García, L.; Rodriguez-Cabezas, M.E.; et al. Anti-Inflammatory and Chemopreventive Effects of Bryophyllum pinnatum (Lamarck) Leaf Extract in Experimental Colitis Models in Rodents. Front. Pharmacol. 2020, 11, 998.

231. Arunachalam, K.; Damazo, A.S.; Macho, A.; Lima, J.C.d.S.; Pavan, E.; Figueiredo, F.d.F.; Oliveira, D.M.; Cechinel-Filho, V.; Wagner, T.M.; Martins, D.T.O. Piper umbellatum L. (Piperaceae): Phytochemical profiles of the hydroethanolic leaf extract and intestinal anti-inflammatory mechanisms on 2,4,6 trinitrobenzene sulfonic acid induced ulcerative colitis in rats. J. Ethnopharmacol. 2020, 254, 112707.

232. Badr, G.; Elsawy, H.; Amalki, M.A.; Alfwuaires, M.; El-Gerbed, M.S.A.; Abdel-Moneim, A.M. Protective effects of myristicin against ulcerative colitis induced by acetic acid in male mice. Food Agric. Immunol. 2020, 31, 435–446.

233. Chen, J.F.; Luo, D.D.; Lin, Y.S.; Liu, Y.H.; Wu, J.Z.; Yi, X.Q.; Wu, Y.; Zhang, Q.;, Gao, C.J.; Cai, J.; et al. Aqueous extract of Bruguiera gymnorrhiza leaves protects against dextran sulfate sodium induced ulcerative colitis in mice via suppressing NF-κB activation and modulating intestinal microbiota. J. Ethnopharmacol. 2020, 251, 112554.

234. Cota, D.; Mishra, S.; Shengule, S. Arjunarishta alleviates experimental colitis via suppressing proinflammatory cytokine expression, modulating gut microbiota and enhancing antioxidant effect. Mol. Biol. Rep. 2020, 47, 7049–7059.

235. Osafo, N.; Essel, L.; Obiri, D.; Antwi, A.; Duduyemi, M.B. Ulcerative colitis induced with acetic acid is ameliorated by Antrocaryon micraster through reduced serum levels of tumor necrosis factor alpha and interleukin-6 in sprague dawley rats. Pharmacogn. Res. 2020, 12, 85–91.

236. Guo, G.; Shi, F.; Zhu, J.; Shao, Y.; Gong, W.; Zhou, G.; Wu, H.; She, J.; Shi, W. Piperine, a functional food alkaloid, exhibits inhibitory potential against TNBS-induced colitis via the inhibition of IκB-α/NF-κB and induces tight junction protein (claudin-1, occludin, and ZO-1) signaling pathway in experimental mice. Hum. Exp. Toxicol. 2020, 39, 477–491.

237. Jeon, Y.-D.; Lee, J.-H.; Lee, Y.-M.; Kim, D.-K. Puerarin inhibits inflammation and oxidative stress in dextran sulfate sodium-induced colitis mice model. Biomed. Pharmacother. 2020, 124, 109847.

238. Kim, H.-Y.; Jeon, H.; Bae, C.H.; Lee, Y.; Kim, H.; Kim, S. Rumex japonicus Houtt. alleviates dextran sulfate sodium-induced colitis by protecting tight junctions in mice. Integr. Med. Res. 2020, 9, 100398.

239. Li, X.; Liu, X.; Zhang, Y.; Zhang, Y.; Liu, S.; Zhang, N.; Wang, D. Protective effect of Gloeostereum incarnatum on ulcerative colitis via modulation of Nrf2/NF-κB signaling in C57BL/6 mice. Mol. Med. Rep. 2020, 22, 3418–3428.

240. Qian, B.; Wang, C.; Zeng, Z.; Ren, Y.; Li, D.; Song, J.-L. Ameliorative Effect of Sinapic Acid on Dextran Sodium Sulfate- (DSS-) Induced Ulcerative Colitis in Kunming (KM) Mice. Oxidative Med. Cell. Longev. 2020, 2020, 8393504.

241. Raj, V.; Venkataraman, B.; Almarzooqi, S.; Chandran, S.; Ojha, S.K.; Attoub, S.; Adrian, T.E.; Subramanya, S.B. Nerolidol Mitigates Colonic Inflammation: An Experimental Study Using both In Vivo and In Vitro Models. Nutrients 2020, 12, 2032.

242. Bastaki, S.M.A.; Amir, N.; Adeghate, E.; Ojha, S. Nerolidol, a sesquiterpene, attenuates oxidative stress and inflammation in acetic acid-induced colitis in rats. Mol. Cell. Biochem. 2021, 476, 3497–3512.

243. Valero, M.S.; González, M.; Ramón-Gimenez, M.; Andrade, P.B.; Moreo, E.; Les, F.; Fernandes, F.; Gómez-Rincón, C.; Berzosa, C.; García de Jalón, J.A.; Arruebo, M.P.; et al. Jasonia glutinosa (L.) DC.; a traditional herbal medicine, reduces inflammation, oxidative stress and protects the intestinal barrier in a murine model of colitis. Inflammopharmacology 2020, 28, 1717–1734.

244. Wu, Z.-C.; Zhao, Z.-L.; Deng, J.-P.; Huang, J.-T.; Wang, Y.-F.; Wang, Z.-P. Sanhuang Shu'ai decoction alleviates DSS-induced ulcerative colitis via regulation of gut microbiota, inflammatory mediators and cytokines. Biomed. Pharmacother. 2020, 125, 109934.

245. Zhang, R.; Yuan, S.; Ye, J.; Wang, X.; Zhang, X.; Shen, J.; Yuan, M.;, Liao, W. Polysaccharide from flammuliana velutipes improves colitis via regulation of colonic microbial dysbiosis and inflammatory responses. Int. J. Biol. Macromol. 2020, 149, 1252–1261.

246. Zhang, Y.; Han, D.; Yu, S.; An, C.; Liu, X.; Zhong, H.; Xu, Y.; Jiang, L.; Wang, Z. Protective Effect of Iridoid Glycosides of the Leaves of Syringa oblata Lindl. on Dextran Sulfate Sodium-Induced Ulcerative Colitis by Inhibition of the TLR2/4/MyD88/NF-κB Signaling Pathway. BioMed Res. Int. 2020, 2020, 7650123.

247. Akl, E.M.; Taha, F.S.; Mohamed, S.S.; Mohamed, R.S. Characterization of Garden Cress Mucilage and its Prophylactic Effect Against Indomethacin-Induced Enter-Colitis in Rats. Biointerface Res. Appl. Chem. 2021, 11, 13911–13923.

248. Ansari, M.N.; Rehman, N.U.; Karim, A.; Soliman, G.A.; Ganaie, M.A.; Raish, M.; Hamad, A.M. Role of Oxidative Stress and Inflammatory Cytokines (TNF-alpha and IL-6) in Acetic Acid-Induced Ulcerative Colitis in Rats: Ameliorated by Otostegia fruticosa. Life 2021, 11, 195.

249. Keshavarzi, Z.; Safari, F.; Alipour, B.; Khoshniat, A.; Azizi, R.; Vatanchian, M.; Maghool, F. Antioxidant Effects of Methanol Extract of Dracocephalum kotschyi in Acetic Acid Induced Rat Colitis Model. J. Adv. Med. Biomed. Res. 2022, 30, 39–46.

250. Liu, X.; Quan, S.; Han, Q.; Li, J.; Gao, X.; Zhang, J.; Liu, D. Effectiveness of the fruit of Rosa odorata sweet var. gigantea (Coll. et Hemsl.) Rehd. et Wils in the protection and the healing of ethanol-induced rat gastric mucosa ulcer based on Nrf2/NF-κB pathway regulation. J. Ethnopharmacol. 2022, 282, 114626.

251. Shin, J.M.; Son, Y.-J.; Ha, I.J.; Erdenebileg, S.; Jung, D.S.; Song, D.-G.; Kim, Y.S.; Kim, S.M.; Nho, CW. Artemisia argyi extract alleviates inflammation in a DSS-induced colitis mouse model and enhances immunomodulatory effects in lymphoid tissues. BMC Complement. Med. Ther. 2022, 22, 64.

252. Cellat, M.; Tekeli, O.; Türk, E.; Aydin, T.; Uyar, A.; İşler, C.T.; Gökçek, İ.; Etyemez, M.; Güvenç, M. Inula viscosa ameliorates acetic acid induced ulcerative colitis in rats. Biotech. Histochem. 2023, 98, 255–266.

253. Chen, G.; Wen, D.; Shen, L.; Feng, Y.; Xiong, Q.; Li, P.; Zhao, Z. Cepharanthine Exerts Antioxidant and Anti-Inflammatory Effects in Lipopolysaccharide (LPS)-Induced Macrophages and DSS-Induced Colitis Mice. Molecules 2023, 28, 6070.

254. Erdenebileg, S.; Son, Y.J.; Kim, M.; Oidovsambuu, S.; Cha, K.H.; Kwon, J.; Jung, D.S.; Nho, C.W. Saposhnikovia divaricata root and its major components ameliorate inflammation and altered gut microbial diversity and compositions in DSS-induced colitis. Integr. Med. Res. 2023, 12, 100998.

255. Fathima, A.; Gangachannaiah, S.; Bose, U.; Kabekkodu, S.P.; Chakraborty, R.; Praveen Kumar, S.E.; Padmanabha, U.; Rachagolla, S.; Prathap, Y.; Vidya, M. Effect of aqueous extract of Trigonellafoenum-graecum L. seeds on Acetic acid- induced Ulcerative colitis in rats. Res. J. Pharm. Technol. 2023, 16, 2161–2168.

256. Ghasemi-Dehnoo, M.; Lorigooini, Z.; Amini-Khoei, H.; Sabzevary-Ghahfarokhi, M.; Rafieian-Kopaei, M. Quinic acid ameliorates ulcerative colitis in rats, through the inhibition of two TLR4-NF-κB and NF-κB-INOS-NO signaling pathways. Immun. Inflamm. Dis. 2023, 11, e926.

257. Gu, D.; Wang, H.; Yan, M.; Li, Y.; Yang, S.; Shi, D.; Wu, L.; Liu, C. Echinacea purpurea (L.) Moench extract suppresses inflammation by inhibition of C3a/C3aR signaling pathway in TNBS-induced ulcerative colitis rats. J. Ethnopharmacol. 2023, 307, 116221.

258. Wei, F.H.; Xie, W.Y.; Zhao, P.S.; Gao, W.; Gao, F. Echinacea purpurea Polysaccharide Ameliorates Dextran Sulfate Sodium-Induced Colitis by Restoring the Intestinal Microbiota and Inhibiting the TLR4-NF-κB Axis. Nutrients 2024, 16, 1305.

259. He, Z.; Liu, J.; Liu, Y. Daphnetin attenuates intestinal inflammation, oxidative stress, and apoptosis in ulcerative colitis via inhibiting REG3A-dependent JAK2/STAT3 signaling pathway. Environ. Toxicol. 2023, 38, 2132–2142.

260. Li, C.; Liu, M.; Deng, L.; Luo, D.; Ma, R.; Lu, Q. Oxyberberine ameliorates TNBS-induced colitis in rats through suppressing inflammation and oxidative stress via Keap1/Nrf2/NF-κB signaling pathways. Phytomedicine 2023, 116, 154899.

261. Li, F.; Huang, H.; Zhao, P.; Jiang, J.; Ding, X.; Lu, D.; Ji, L. Curculigoside mitigates dextran sulfate sodium-induced colitis by activation of KEAP1-NRF2 interaction to inhibit oxidative damage and autophagy of intestinal epithelium barrier. Int. J. Mol. Med. 2023, 52, 107.

262. Minaiyan, M.; Pasandideh-Fetrat, P.; Sadeghi-Dinani, M.; Talebi, A. Ameliorative Effect of Aqueous and Hydroalcoholic Extracts of Scrophularia striata Boiss. on Murine Model of Experimental Colitis. Adv. Biomed. Res. 2023, 12, 105.

263. Otu-Boakye, S.A.; Yeboah, K.O.; Boakye-Gyasi, E.; Oppong-Kyekyeku, J.; Okyere, P.D.; Osafo, N. Acetic acid-induced colitis modulating potential of total crude alkaloidal extract of Picralima nitida seeds in rats. Immunity Inflamm. Dis. 2023, 11, e855.

264. Puppala, E.R.; Yalamarthi, S.S.; Aochenlar, S.L.; Prasad, N.; Syamprasad, N.P.; Singh, M.; Nanjappan, S.K.; Ravichandiran, V.; Tripathi, D.M.; Gangasani, J.K. Mesua assamica (King&Prain) kosterm. Bark ethanolic extract attenuates chronic restraint stress aggravated DSS-induced ulcerative colitis in mice via inhibition of NF-κB/STAT3 and activation of HO-1/Nrf2/SIRT1 signaling pathways. J. Ethnopharmacol. 2023, 301, 115765.

265. Shao, X.X.; Xu, Y.; Xiao, H.Y.; Hu, Y.; Jiang, Y. Higenamine improves DSS-induced ulcerative colitis in mice through the Galectin-3/TLR4/NF-κB pathway. Tissue Cell 2023, 82, 102111.

266. Tan, Z.; Zhang, Q.; Zhao, R.; Huang, T.; Tian, Y.; Lin, Y. A Comparative Study on the Effects of Different Sources of Carboxymethyl Poria Polysaccharides on the Repair of DSS-Induced Colitis in Mice. Int. J. Mol. Sci. 2023, 24, 9034.

267. Venkataraman, B.; Almarzooqi, S.; Raj, V.; Bhongade, B.A.; Patil, R.B.; Subramanian, V.S.; Attoub, S.; Rizvi, T.A.; Adrian, T.E.; Subramanya, S.B. Molecular Docking Identifies 1,8-Cineole (Eucalyptol) as A Novel PPARγ Agonist That Alleviates Colon Inflammation. Int. J. Mol. Sci. 2023, 24, 6160.

268. Xu, J.; Tang, C.; Din, A.U.; Lu, Y.; Ma, X.; Zhang, T.; Wu, J.; Zuoqin, D.; Luo, P.; Wu, J. Oligosaccharides of Polygonatum Cyrtonema Hua ameliorates dextran sulfate sodium-induced colitis and regulates the gut microbiota. Biomed. Pharmacother. 2023, 161, 114562.

269. Zhang, J.; Cheng, S.; Liang, J.; Qu, J. Polysaccharide from fermented mycelium of Inonotus obliquus attenuates the ulcerative colitis and adjusts the gut microbiota in mice. Microb. Pathog. 2023, 177, 105990.

270. Minaiyan, M.; Dastanian, Z.; Zolfaghari, B.; Talebi, A. Assessing the Anti-Colitis Properties of Aqueous and Hydroalcoholic Extracts of Pinus eldarica in Rats with Acetic Acid-Induced Colitis. Res. J. Pharmacogn. 2024, 11, 61–70.

271. Dong, W.-R.; Li, Y.-Y.; Liu, T.-T.; Zhou, G.; Chen, Y.-X. Ethyl acetate extract of Terminalia chebula alleviates DSS-induced ulcerative colitis in C57BL/6 mice. Front. Pharmacol. 2023, 14, 1229772.

272. Kim, Y.-M.; Kim, H.-Y.; Jang, J.-T.; Hong, S. Preventive Effect of Ecklonia cava Extract on DSS-Induced Colitis by Elevating Intestinal Barrier Function and Improving Pathogenic Inflammation. Molecules 2023, 28, 8099.

273. Vezza, T.; Molina-Tijeras, J.A.; Rodríguez-Nogales, A.; Garrido-Mesa, J.; Cádiz-Gurrea, M.d.l.L.; Segura-Carretero, A.; González-Tejero, M.R.; Rodríguez-Cabezas, M.E.;, Gálvez, J.; Algieri, F. The Antioxidant Properties of Salvia verbenaca Extract Contribute to Its Intestinal Antiinflammatory Effects in Experimental Colitis in Rats. Antioxidants 2023, 12, 2071.

274. Da Silva, V.C.; Guerra, G.C.B.; Araújo, D.F.D.S.; De Araújo, E.R.; De Araújo, A.A.; Dantas-Medeiros, R.; Zanatta, A.C.; Da Silva, I.L.G.; De Araújo Júnior, R.F.; et al. Chemopreventive and immunomodulatory

effects of phenolic-rich extract of Commiphora leptophloeos against inflammatory bowel disease: Preclinical evidence. J. Ethnopharmacol. 2024, 328, 118025.

275. Feng, Y.; Chen, S.; Song, Y.; Liu, S.; Duan, Y.; Cai, M.; Kong, T.; Zhang, H. A novel Sagittaria sagittifolia L. polysaccharides mitigate DSS-induced colitis via modulation of gut microbiota and MAPK/NF-κB signaling pathways. Int. J. Biol. Macromol. 2024, 254, 127835.

276. Feng, Z.; Ye, W.; Feng, L. Bioactives and metabolites of Tetrastigma hemsleyanum root extract alleviate DSS-induced ulcerative colitis by targeting the SYK protein in the B cell receptor signaling pathway. J. Ethnopharmacol. 2024, 322, 117563.

277. Gülada, B..; Cam, M.E.; Yüksel, M.; Akakın, D.; Taşkın, T.; Emre, G.; Şener, G.; Karakoyun, B. Gilaburu (Viburnum opulus L.) fruit extract has potential therapeutic and prophylactic role in a rat model of acetic acid-induced oxidant colonic damage. J. Ethnopharmacol. 2024, 322, 117624.

278. Hui, H.; Wang, Z.; Zhao, X.; Xu, L.; Yin, L.; Wang, F.; Qu, L.; Peng, J. Gut microbiome-based thiamine metabolism contributes to the protective effect of one acidic polysaccharide from Selaginella uncinata (Desv.) Spring against inflammatory bowel disease. J. Pharm. Anal. 2024, 14, 177–195.

279. Li, W.; Wang, Y.; Zhang, Y.; Fan, Y.; Liu, J.; Zhu, K.; Jiang, S.; Duan, J. Lizhong decoction ameliorates ulcerative colitis by inhibiting ferroptosis of enterocytes via the Nrf2/SLC7A11/GPX4 pathway. J. Ethnopharmacol. 2024, 326, 117966.

280. Liu, Y.; Wu, J.; Tan, L.; Li, Z.; Gao, P.; He, S.; Wang, Q.; Tang, D.; Wang, C.; Wang, F.; et al. (-)-Syringaresinol attenuates ulcerative colitis by improving intestinal epithelial barrier function and inhibiting inflammatory responses. Phytomedicine 2024, 124, 155292.

281. Shah, B.; Solanki, N. Aegeline attenuates TNBS-induced colitis by suppressing the -NF-κB -mediated NLRP3 inflammasome pathway in mice. Inflammopharmacology 2024, 32, 2589–2599.

282. Sun, X.; Jin, X.; Wang, L.; Lin, Z.; Feng, H.; Zhan, C.; Liu, X.; Cheng, G. Fraxetin ameliorates symptoms of dextran sulphate sodium-induced colitis in mice. Heliyon 2024, 10, e23295.

283. Zang, Z.; Li, L.; Yang, M.; Zhang, H.; Naeem, A.; Wu, Z.; Zheng, Q.; Song, Y.; Tao, L.; Wan, Z.; et al. Study on the ameliorative effect of honeysuckle on DSS-induced ulcerative colitis in mice. J. Ethnopharmacol. 2024, 325, 117776.

284. Cazarin, C.B.B.; Rodriguez-Nogales, A.; Algieri, F.; Utrilla, M.P.; Rodríguez-Cabezas, M.E.; Garrido-Mesa, J.; Guerra-Hernández, E.; Braga, P.A.C.; Reys, F.G.R.; et al. Intestinal anti-inflammatory effects of Passiflora edulis peel in the dextran sodium sulphate model of mouse colitis. J. Funct. Foods 2016, 26, 565–576.

285. Shao, X.; Sun, C.; Tang, X.; Zhang, X.; Han, D.; Liang, S.; Qu, R.; Hui. X.; Shan, Y.; Hu, L.; et al. Anti-Inflammatory and Intestinal Microbiota Modulation Properties of Jinxiang Garlic (Allium sativum L.) Polysaccharides toward Dextran Sodium Sulfate-Induced Colitis. J. Agric. Food Chem. 2020, 68, 12295–12309.

286. Elhennawy, M.G.; Abdelaleem, E.A.; Zaki, A.A.; Mohamed, W.R. Cinnamaldehyde and hesperetin attenuate TNBS-induced ulcerative colitis in rats through modulation of the JAk2/STAT3/SOCS3 pathway. J. Biochem. Mol. Toxicol. 2021, 35, e22730.

287. Siracusa, R.; Fusco, R.; Peritore, A.F.; Cordaro, M.; D'Amico, R.; Genovese, T.; Gugliandolo. E.; Crupi, R.; Smeriglio, A.; Mandalari, G.; et al. The Antioxidant and Anti-Inflammatory Properties of Anacardium occidentale L. Cashew Nuts in a Mouse Model of Colitis. Nutrients 2020, 12, 834.

288. Prakash, A.N.; Prasad, N.; Puppala, E.R.; Panda, S.R.; Jain, S.; Ravichandiran, V.; Singh, M.; Naidu, V.G.M. Loganic acid protects against ulcerative colitis by inhibiting TLR4/NF-κB mediated inflammation

and activating the SIRT1/Nrf2 anti-oxidant responses in-vitro and in-vivo. Int. Immunopharmacol. 2023, 122, 110585

289. Chen, Z.; Wang, H.; Tan, L.; Liu, X. Protective Effects of Four Structurally Distinct Sanshools Ameliorate Dextran Sodium Sulfate-Induced Ulcerative Colitis by Restoring Intestinal Barrier Function and Modulating the Gut Microbiota. Antioxidants 2024, 13, 153.

290. Ouyang, Q.; Li, X.; Liang, Y.; Liu, R. Sea Buckthorn Polysaccharide Ameliorates Colitis. Nutrients 2024, 16, 1280.

291. Liu, B.; Lin, Q.; Yang, T.; Zeng, L.; Shi, L.; Chen, Y.; Luo, F. Oat β-glucan ameliorates dextran sulfate sodium (DSS)-induced ulcerative colitis in mice. Food Funct. 2015, 6, 3454–3463.

292. Pervin, M.; Hasnat, A.; Lim, J.-H.; Lee, Y.-M.; Kim, E.O.; Um, B.-H.; Lim, B.O. Preventive and therapeutic effects of blueberry (Vaccinium corymbosum) extract against DSS-induced ulcerative colitis by regulation of antioxidant and inflammatory mediators. J. Nutr. Biochem. 2016, 28, 103–113.

293. Karakoyun, B.; Ertaş, B.; Yüksel, M.; Akakın, D.; Çevik, Ú.; Şener, G. Ameliorative effects of riboflavin on acetic acid-induced colonic injury in rats. Clin. Exp. Pharmacol. Physiol. 2017, 45, 563–572.

294. Araújo, D.F.D.S.; Guerra, G.C.B.; Júnior, R.F.D.A.; Antune,s de Araújo, A.; Antonino de Assis, P.O.; Nunes de Medeiros, A.; et al. Goat whey ameliorates intestinal inflammation on acetic acid-induced colitis in rats. J. Dairy Sci. 2016, 99, 9383–9394.

295. Balaha, M.; Kandeel, S.; Elwan, W. Garlic oil inhibits dextran sodium sulfate-induced ulcerative colitis in rats. Life Sci. 2016, 146, 40–51.

296. Choi, K.-C.; Cho, S.-W.; Lee, J.-C. Red bean extracts protect rats against intestinal inflammatory damage. Food Sci. Biotechnol. 2016, 25, 349–353.

297. Colares, J.R.; Schemitt, E.G.; Hartmann, R.M.; Moura, R.M.; Morgan-Martins, M.I.; Fillmann, H.S.; Fillmann, L.; & Marroni, N. P. Effect of lecithin on oxidative stress in an experimental model of rats colitis induced by acetic acid. J. Coloproctology 2016, 36, 97–103.

298. Ewees, M.G.; Messiha, B.A.S.; Abo-Saif, A.A.; El-Latif, H.A. Is Coenzyme Q10 Effective in Protection against Ulcerative Colitis? An Experimental Study in Rats. Biol. Pharm. Bull. 2016, 39, 159–166.

299. Khodir, A.E.; Atef, H.; Said, E.; ElKashef, H.A.; Salem, H.A. Implication of Nrf2/HO-1 pathway in the coloprotective effect of coenzyme Q10 against experimentally induced ulcerative colitis. Inflammopharmacology 2017, 25, 119–135.

300. Nooh, H.Z.; Nour-Eldien, N.M. The dual anti-inflammatory and antioxidant activities of natural honey promote cell proliferation and neural regeneration in a rat model of colitis. Acta Histochem. 2016, 118, 588–595.

301. Palla, A.H.; Iqbal, N.T.; Minhas, K.; Gilani, A.-H. Flaxseed extract exhibits mucosal protective effect in acetic acid induced colitis in mice by modulating cytokines, antioxidant and antiinflammatory mechanisms. Int. Immunopharmacol. 2016, 38, 153–166.

302. Shi, L.; Lin, Q.; Yang, T.; Nie, Y.; Li, X.; Liu, B.; Shen, J.; Liang, Y.; Tang, Y.; Luo, F. Oral administration of Lentinus edodes β-glucans ameliorates DSS-induced ulcerative colitis in mice via MAPK-Elk-1 and MAPK-PPARγpathways. Food Function 2016, 7, 4614–4627.

303. Kaur, R.; Thakur, S.; Rastogi, P.; Kaushal, N. Resolution of Cox mediated inflammation by Se supplementation in mouse experimental model of colitis. PLoS ONE 2018, 13, e0201356.

304. Shi, C.; Yue, F.; Shi, F.; Qin, Q.; Wang, L.; Wang, G.; Mu, L.; Liu, D.; Li, Y.; Yu.; et al. Selenium-Containing Amino Acids Protect Dextran Sulfate Sodium-Induced Colitis via Ameliorating Oxidative Stress and Intestinal Inflammation. J. Inflamm. Res. 2021, 14, 85–95.

305. Li, T.; Zhu, K.; Wang, L.; Dong, Y.; Huang, J. Stabilization by Chaperone GroEL in Biogenic Selenium Nanoparticles Produced from Bifidobacterium animalis H15 for the Treatment of DSS-Induced Colitis. ACS Appl. Mater. Interfaces 2024, 16, 13439–13452.

306. Lee, W.T.; Tung, Y.T.; Wu, C.C.; Tu, P.S.; Yen, G.C. Camellia Oil (Camellia oleifera Abel.) Modifies the Composition of Gut Microbiota and Alleviates Acetic Acid-Induced Colitis in Rats. J. Agric. Food Chem. 2018, 66, 7384–92.

307. Maghool, F.; Keshavarzi, Z.; Nurmohammadi, F.; Majlesi, S.; Maghool, F. Protective effects of walnut extract against oxidative damage in acetic acid-induced experimental colitis rats. Physiol. Pharmacol. 2019, 36, 2096–103.

308. Salamatian, M.; Mohammadi, V.; Froushani, S.M.A. Ameliorative effects of aqueous cinnamon extract on ulcerative colitis in rats. Physiol. Pharmacol. 2019, 23, 140–149.

309. Kim, M.S.; Kim, Y.D.; Kang, S.; Kwon, O.; Shin, J.-H.; Kim, J.Y. Cinnamon(Cinnamomum japonicum) subcritical water extract suppresses gut damage induced by dextran sodium sulfate in mouse colitis model. J. Funct. Foods 2021, 87, 104775.

310. Saw, T.Y.; Malik, N.A.; Lim, K.P.; Teo, C.W.L.; Wong, E.S.M.; Kong, S.C.; Fong, C.W.; Petkov, J.; Yap, W.N. Oral Supplementation of Tocotrienol-Rich Fraction Alleviates Severity of Ulcerative Colitis in Mice. J. Nutr. Sci. Vitaminol. 2019, 65, 318–327.

311. Sharma, M.; Kaur, R.; Kaushik, K.; Kaushal, N. Redox modulatory protective effects of omega-3 fatty acids rich fish oil against experimental colitis. Toxicol. Mech. Methods 2019, 29, 244–254.

312. El Mahdy, R.N.; Nader, M.A.; Helal, M.G.; Abu-Risha, S.E.; Abdelmageed, M.E. Eicosapentaenoic acid mitigates ulcerative colitis-induced by acetic acid through modulation of NF-κB and TGF-α/ EGFR signaling pathways. Life Sci. 2023, 327, 121820.

313. Kwon, S.-H.; Kothari, D.; Jung, H.-I.; Lim, J.-M.; Kim, W.-L.; Kwon, H.-C.; et al. Noni juice-fortified yogurt mitigates dextran sodium sulfate-induced colitis in mice through the modulation of inflammatory cytokines. . J. Funct. Foods 2021, 86, 104652.

314. Deng, Z.; Cui, C.; Wang, Y.; Ni, J.; Zheng, L.; Wei, H.K.; Peng, J. FSGHF3 and peptides, prepared from fish skin gelatin, exert a protective effect on DSS-induced colitis via the Nrf2 pathway. Food Funct. 2020, 11, 414–423.

315. Li, P.; Xiao, N.; Zeng, L.; Xiao, J.; Huang, J.; Xu, Y.; Chen, Y.; Ren, Y.; Du, B. Structural characteristics of a mannoglucan isolated from Chinese yam and its treatment effects against gut microbiota dysbiosis and DSS-induced colitis in mice. Carbohydr. Polym. 2020, 250, 116958.

316. Ganapathy, A.S.; Saha, K.; Wang, A.; Arumugam, P.; Dharmaprakash, V.; Yochum, G.; Koltun, W.; Nighot, M.; Perdew, G.; Thompson, T.A.;. Alpha-tocopherylquinone differentially modulates claudins to enhance intestinal epithelial tight junction barrier via AhR and Nrf2 pathways. Cell Rep. 2023, 42, 112705–112705.

317. Zhu, L.; Song, Y.; Liu, H.; Wu, M.; Gong, H.; Lan, H.; Zheng, X. Gut microbiota regulation and anti-inflammatory effect of β-carotene in dextran sulfate sodium-stimulated ulcerative colitis in rats. J. Food Sci. 2021, 86, 2118–2130.

318. Wang, F.; Yuan, M.; Shao, C.; Ji, N.; Zhang, H.; Li, C. Momordica charantia-Derived Extracellular Vesicles Provide Antioxidant Protection in Ulcerative Colitis. Molecules 2023, 28, 6182.

319. Prabha, S.; Tamoli, S.; Raghavamenon, A.C.; Manu, K.A. Virgin Coconut Oil Alleviates Dextran Sulphate-Induced Inflammatory Bowel Disease and Modulates Inflammation and Immune Response in Mice. J. Am. Nutr. Assoc. 2024, 43, 61–71.

320. Wu, M.; Wang, Q.; Li, X.; Yu, S.; Zhao, F.; Wu, X.; Fan, L.; Liu, X.; Zhao, Q.; He, X.; Li, W.; et al. Gut microbiota-derived 5-hydroxyindoleacetic acid from pumpkin polysaccharides supplementation alleviates colitis via MAPKs-PPARγ/NF-κB inhibition. Int. J. Biol. Macromol. 2024, 264, 130385.

321. Yoon, J.W.; Ahn, S.I.; Jhoo, J.W.; Kim, G.Y. Antioxidant Activity of Yogurt Fermented at Low Temperature and Its Anti-inflammatory Effect on DSS-induced Colitis in Mice. Food Sci. Anim. Resour. 2019, 39, 162–176.

322. Chen, C.-L.; Hsu, P.-Y.; Pan, T.-M. Therapeutic effects of Lactobacillus paracasei subsp. paracasei NTU 101 powder on dextran sulfate sodium-induced colitis in mice. J. Food Drug Anal. 2018, 27, 83–92.

323. Choi, S.-H.; Lee, S.-H.; Kim, M.G.; Lee, H.J.; Kim, G.-B. Lactobacillus plantarum CAU1055 ameliorates inflammation in lipopolysaccharide-induced RAW264.7 cells and a dextran sulfate sodium-induced colitis animal model. J. Dairy Sci. 2019, 102, 6718–6725.

324. Catinean, A.; Neag, M.A.; Krishnan, K.; Muntean, D.M.; Bocsan, C.I.; Pop, R.M.; Mitre, A.O.; Melincovici, C.S.; Buzoianu, A.D. Probiotic Bacillus Spores Together with Amino Acids and Immunoglobulins Exert Protective Effects on a Rat Model of Ulcerative Colitis. Nutrients 2020, 12, 3607.

325. Din, A.U.; Hassan, A.; Zhu, Y.; Zhang, K.; Wang, Y.; Li, T.; Wang, Y.; Wang, G. Inhibitory effect of Bifidobacterium bifidum ATCC 29521 on colitis and its mechanism. J. Nutr. Biochem. 2020, 79, 108353.

326. Hu, T.; Wang, H.; Xiang, C.; Mu, J.; Zhao, X. Preventive Effect of Lactobacillus acidophilus XY27 on DSS-Induced Ulcerative Colitis in Mice. Drug Des. Dev. Ther. 2020, 14, 5645–5657.

327. Cordeiro, B.F.; Alves, J.L.; Belo, G.A.; Oliveira, E.R.; Braga, M.P.; da Silva, S.H.; Lemos, L.; Guimarães, J.T.; Silva, R.; Rocha, R.S.; et al. Therapeutic Effects of Probiotic Minas Frescal Cheese on the Attenuation of Ulcerative Colitis in a Murine Model. Front. Microbiol. 2021, 12, 623920.

328. Gao, H.; Li, Y.; Sun, J.; Xu, H.; Wang, M.; Zuo, X.; Fu, Q.; Guo, Y.; Chen Z, Zhang P. Saccharomyces boulardii Ameliorates Dextran Sulfate Sodium-Induced Ulcerative Colitis in Mice by Regulating NF-κB and Nrf2 Signaling Pathways. Oxidative Med. Cell. Longev. 2021, 2021, 1622375.

329. Shi, J.; Xie, Q.; Yue, Y.; Chen, Q.; Zhao, L.; Evivie, S.E.; Li, B.; Huo, G. Gut microbiota modulation and anti-inflammatory properties of mixed lactobacilli in dextran sodium sulfate-induced colitis in mice. Food Funct. 2021, 12, 5130–5143.

330. Jeong, Y.; Kim, D.H.; Lee, K.W. Homeostasis effects of fermented Maillard reaction products by Lactobacillus gasseri 4M13 in dextran sulfate sodium-induced colitis mice. J. Sci. Food Agric. 2022, 102, 434–444.

331. Kangwan, N.; Kongkarnka, S.; Boonkerd, N.; Unban, K.; Shetty, K.; Khanongnuch, C. Protective Effect of Probiotics Isolated from Traditional Fermented Tea Leaves (Miang) from Northern Thailand and Role of Synbiotics in Ameliorating Experimental Ulcerative Colitis in Mice. Nutrients 2022, 14, 227.

332. Liu, Q.; Jian, W.; Wang, L.; Yang, S.; Niu, Y.; Xie, S.J.; Hayer, K.; Chen, K.; Zhang, Y.; Guo, Y.; et al. Alleviation of DSS-induced colitis in mice by a new-isolated Lactobacillus acidophilus C4. Front. Microbiol. 2023, 14, 11.

333. Wang, H.; Zhang, X.; Kou, X.; Zhai, Z.; Hao, Y. A Ropy Exopolysaccharide-Producing Strain Bifidobacterium pseudocatenulatum Bi-OTA128 Alleviates Dextran Sulfate Sodium-Induced Colitis in Mice. Nutrients 2023, 15, 4993.

334. Ma, X.W.; Hu, Y.C.; Li, X.; Zheng, X.T.; Wang, Y.T.; Zhang, J.M.; Fu, C.; Geng, F. Periplaneta americana Ameliorates Dextran Sulfate Sodium-Induced Ulcerative Colitis in Rats by Keap1/Nrf-2 Activation, Intestinal Barrier Function, and Gut Microbiota Regulation. Front. Pharmacol. 2018, 9, 944.

335. Peng, S.; Shen, L.; Yu, X.; Wu, J.; Zha, L.; Xia, Y.; Luo, H. miR-200a attenuated oxidative stress, inflammation, and apoptosis in dextran sulfate sodium-induced colitis through activation of Nrf2. Front. Immunol. 2023, 14, 1196065.

336. Wu, J.; Zhang, Z.; Wu, Q.; Zhang, L.; Chen, Z.; Zhao, H.; Wu, X.; Zhao, Y.; Zhang, C.; Ge, J. Antioxidative effect of Periplaneta americana extract on dextran sulfate sodium-induced ulcerative colitis through activation of the Nrf2 signal. Pharm. Biol. 2023, 61, 949–962.

337. Abd-Ellatieff, H.A.; Georg, K.; Abourawash, A.-R.A.; Ghazy, E.W.; Samak, D.H.; Goda, W.M. Aspergillus awamori: Potential antioxidant, anti-inflammatory, and anti-apoptotic activities in acetic acid-induced ulcerative colitis in rats. Inflammopharmacology 2024, 32, 2541–2553.

338. Wang, R.; Luo, Y.; Lu, Y.; Wang, D.; Wang, T.; Pu, W.; Wang, Y. Maggot Extracts Alleviate Inflammation and Oxidative Stress in Acute Experimental Colitis via the Activation of Nrf2. Oxidative Med. Cell. Longev. 2019, 2019, 4703253.

339. Zbakh, H.; Talero, E.; Avila, J.; Alcaide, A.; de los Reyes, C.; Zubia, E.; Motilva, V. The Algal Meroterpene 11-Hydroxy-1 '-O-Methylamentadione Ameloriates Dextran Sulfate Sodium-Induced Colitis in Mice. Mar. Drugs 2016, 14, 149.

340. Garcia, F.A.O.; Sales-Campos, H.; Yuen, V.G.; Machado, J.R.; Viana, G.S.B.; Oliveira, C.J.F.; McNeill, J.H. Arthrospira (Spirulina) platensis Attenuates Dextran Sulfate Sodium-induced Colitis in Mice by Suppressing Key Pro-inflammatory Cytokines. Korean, J. Gastroenterol. 2020, 76, 150–158.

341. Morsy, M.A.; Gupta, S.; Nair, A.B.; Venugopala, K.N.; Greish, K.; El-Daly, M. Protective Effect of Spirulina platensis Extract against Dextran-Sulfate-Sodium-Induced Ulcerative Colitis in Rats. Nutrients 2019, 11, 2309.

342. Hua, Z.; Hui, L.; Haihua, W. Potential protective effects of the water-soluble Chinese propolis on experimental ulcerative colitis. J. Tradit. Chin. Med. 2023, 43, 925–933.

343. Lu, K.; Liu, L.; Lin, P.; Dong, X.; Ni, L.; Che, H.; Xie, W. Saccharina japonica Ethanol Extract Ameliorates Dextran Sulfate Sodium-Induced Colitis via Reshaping Intestinal Microenvironment and Alleviating Inflammatory Response. Foods 2023, 12, 1671.

344. Zizzo, M.G.; Caldara, G.; Bellanca, A.; Nuzzo, D.; Di Carlo, M.; Scoglio, S.; Serio, R. AphaMax®, an Aphanizomenon Flos-Aquae Aqueous Extract, Exerts Intestinal Protective Effects in Experimental Colitis in Rats. Nutrients 2020, 12, 3635.

345. Xie, J.; Liu, L.; Li, H.; Che, H.; Xie, W.. Ink melanin from Sepiapharaonis ameliorates colitis in mice via reducing oxidative stress, andprotecting the intestinal mucosal barrier. Food Res. Int. 2022, 151, 110888.

346. Xiang, X.-W.; Zhou, X.-L.; Wang, R.; Shu, C.-H.; Zhou, Y.-F.; Ying, X.-G.; Zheng, B. Protective Effect of Tuna Bioactive Peptide on Dextran Sulfate Sodium-Induced Colitis in Mice. Mar. Drugs 2021, 19, 127.

347. Guo, H.-X.; Wang, B.-B.; Wu, H.-Y.; Feng, H.-Y.; Zhang, H.-Y.; Gao, W.; Yuan, B. Turtle peptide and its derivative peptide ameliorated DSS-induced ulcerative colitis by inhibiting inflammation and modulating the composition of the gut microbiota. Int. Immunopharmacol. 2024, 132, 112024.

348. Ávila-Román, J.; Talero, E.; Rodríguez-Luna, A.; García-Mauriño, S.; Motilva, V. Anti-inflammatory effects of an oxylipin-containing lyophilised biomass from a microalga in a murine recurrent colitis model. Br. J. Nutr. 2016, 116, 2044–2052.

349. Nemoto, M.; Kuda, T.; Eda, M.; Yamakawa, H.; Takahashi, H.; Kimura, B.. Protective Effects of Mekabu Aqueous Solution Fermented by Lactobacillus plantarum Sanriku-SU7 on Human Enterocyte-Like HT-29-luc Cells and DSS-Induced Murine IBD Model. Probiotics Antimicrob. Proteins 2017, 9, 48–55.
